# Auxiliary screening COVID-19 by computed tomography

**DOI:** 10.3389/fpubh.2023.974542

**Published:** 2023-06-05

**Authors:** Xiongfeng Pan, Yuyao Chen, Atipatsa C. Kaminga, Shi Wu Wen, Hongying Liu, Peng Jia, Aizhong Liu

**Affiliations:** ^1^Department of Epidemiology and Health Statistics, Xiangya School of Public Health, Central South University, Changsha, China; ^2^Department of Mathematics and Statistics, Mzuzu University, Mzuzu, Malawi; ^3^Obstetrics & Maternal Newborn Investigations Research Group, Ottawa Hospital Research Institute, Ottawa, ON, Canada; ^4^Department of Obstetrics and Gynaecology, Faculty of Medicine, University of Ottawa, Ottawa, ON, Canada; ^5^Faculty of Medicine, School of Epidemiology and Public Health, University of Ottawa, Ottawa, ON, Canada; ^6^School of Resource and Environmental Sciences, Wuhan University, Wuhan, China; ^7^Hubei Luojia Laboratory, Wuhan, China; ^8^School of Public Health, Wuhan University, Wuhan, China; ^9^International Institute of Spatial Lifecourse Health (ISLE), Wuhan University, Wuhan, China

**Keywords:** cross-disciplinary methods, nucleic acid detection, computed tomography, novel coronavirus, COVID-19, real world

## Abstract

**Background:**

The 2019 novel coronavirus (COVID-19) pandemic remains rampant in many countries/regions. Improving the positive detection rate of COVID-19 infection is an important measure for the control and prevention of this pandemic. This meta-analysis aims to systematically summarize the current characteristics of the computed tomography (CT) auxiliary screening methods for COVID-19 infection in the real world.

**Methods:**

Web of Science, Cochrane Library, Embase, PubMed, CNKI, and Wanfang databases were searched for relevant articles published prior to 1 September 2022. Data on specificity, sensitivity, positive/negative likelihood ratio, area under curve (AUC), and diagnostic odds ratio (dOR) were calculated purposefully.

**Results:**

One hundred and fifteen studies were included with 51,500 participants in the meta-analysis. Among these studies, the pooled estimates for AUC of CT in confirmed cases, and CT in suspected cases to predict COVID-19 diagnosis were 0.76 and 0.85, respectively. The CT in confirmed cases dOR was 5.51 (95% CI: 3.78–8.02). The CT in suspected cases dOR was 13.12 (95% CI: 11.07–15.55).

**Conclusion:**

Our findings support that CT detection may be the main auxiliary screening method for COVID-19 infection in the real world.

## 1. Introduction

Three unprecedented outbreaks of human coronavirus (HCoV) at the beginning of the twenty-first century, indicated coronavirus as a major public health problem worldwide (1, 2). Less than a decade after the last human disease outbreak, caused by the Middle East Respiratory Syndrome Coronavirus (MERS-CoV) in 2012, a new outbreak of the severe acute respiratory syndrome, caused by coronavirus 2 (SARS-CoV-2), is spreading around the world (2). This pandemic is now defined as the 2019 novel coronavirus (COVID-19). The virus was primarily spread by COVID-19 infected individuals. Therefore, the main way to control the spread of COVID-19 disease is to isolate the source of infection. In this regard, the differential screening of COVID-19 highlights the necessity for readily available, accurate and fast screening testing methods (3).

The current gold standard for etiological diagnosis of COVID-19 infection is real-time reverse transcription polymerase chain reaction (RT-PCR) in respiratory specimens (4). However, some studies have shown some problems in using this method to detect COVID-19 infection, such as low sensitivity and specificity (5). These studies suggested that causes of these problems may include performing screening outside the diagnostic window, virus mutation and recombination, using insufficiently validated tests, and instrument failure (5). Therefore, more and more studies are taking cross-disciplinary methods to better understand screening and diagnosis of COVID-19 infection in order to improve diagnostic accuracy, for example, by combining clinical evidence, chest computed tomography (CT), and interpreting RT-PCR results on the basis of epidemiological, clinical, and CT evidence (6). Chest CT could accurately and rapidly evaluate the extent of lung lesions caused by COVID-19, which plays an important role in early detection and is valuable for the diagnosis of COVID-19 pneumonia (7, 8). At the same time, CT scans whose sensitivity can reach 67–100% can compensate for the problem of moderate sensitivity (53–88%) of RT-PCR and have extra value in the diagnosis of COVID-19 (9).

However, none of the current meta-analyses have systematically summarized these strategies (10–12). Although the association between CT and SARS-CoV-2 infection has been discussed in some recently published meta-analyses, the studies included in these studies are relatively limited, the number of studies included ranged from 9 to 15 (13–15).

This study aims to 1) systematically summarize the sensitivity and specificity of the major screening methods for COVID-19, 2) analyze the possible causes of false negatives or false positives as regards the efficacy of the screening methods, and 3) explore how to further improve the sensitivity and specificity of the screening methods. The study will also discuss epidemiological methods that could be used to further help identify more patients with latent infections by investigating their exposure history. It was speculated that, in a public health emergency, a combination of multidisciplinary approaches may be of some help in the control and prevention of COVID-19.

## 2. Methods

### 2.1. Search strategy and selection criteria

Standard procedures for meta-analyses according to the Cochrane Handbook; and the Preferred Reporting Items for Systematic Reviews and Meta-Analyses (PRISMA) guidelines were used to conduct this study (16). Thus, using these procedures, two independent reviewers (XP and AK) systematically searched in the electronic databases, Web of Science, Cochrane Library, Embase, PubMed, CNKI, and Wanfang, for relevant studies published on 1 September 2022, or earlier. Then, from the search outcomes, they selected eligible studies according to the purpose of this study, using predefined selection criteria. Noteworthy, China has accumulated valuable experience in screening and diagnosing COVID-19, but very much literature related to China on this subject has been published in Chinese language. Therefore, to avoid publication bias, gray literature, and studies published in Chinese were included in this study. That is, studies published in English or Chinese were included in this study. In order to retrieve as much literature as possible, the search strategy, among other important terms, included the professional name, COVID-19, and its variant names. Based on both historical and current COVID-19 names, Boolean operators, truncation, and wildcards were used appropriately to include all other variant names for COVID-19. The complete search strategy is shown in the Appendix 1. Experienced librarians designed the search strategy and adjusted it to meet the requirements of each of the databases specified above.

### 2.2. Study selection

Selection of studies for this meta-analysis was based on the following inclusion criteria: (1) diagnostic and screening studies; (2) studies reported methods for diagnosing COVID-19; and (3) original studies. Studies were excluded based on the following criteria: (1) conference papers, case reports, letters, or reviews; and (2) studies not on humans. The details about the excluded studies are shown in the Appendix 1.

### 2.3. Data extraction

Two reviewers (YC and AK) translated the Chinese articles into English, and entered all the data and removed duplicates in EndNote (version x9.1), Then used custom data extraction tool, EpiData (version 3.0) grids, and extracted (17, 18). The key characteristics of interest were extracted from each eligible study in the EpiData (version 3.0) grids, including first author, year of publication, study area, number of subjects, sensitivity, and specificity. Any differences between the two reviewers were resolved by consensus involving the third reviewer (AL).

### 2.4. Quality evaluation

The Quality Assessment of Diagnostic Accuracy Studies-2 checklist (QUADAS-2) was used to assess the quality of the eligible studies. The selected studies were grouped based on their score into high (6–7 points), moderate (4–5 points), and low (0–3 points) quality categories (19, 20).

### 2.5. Patient and public involvement

There were no patients involved in our study.

### 2.6. Statistical analysis

In this study, meta-analyses were carried out using MetaDiSc (version 1.4) and R software (version R i386 3.4.2). For the diagnostic meta-analysis, the number of subjects with a true-positive (TP), false-positive (FP), true-negative (TN), and false-negative (FN) values for each study unit was extracted to calculate the pooled sensitivity [TP/(TP + FN)], specificity [TN/(TN + FP)], positive likelihood ratio (PLR) [(sensitivity/(1–sensitivity)], negative likelihood ratio (NLR), [(1–specificity)/specificity)], diagnostic odds ratio (dOR) [PLR/NLR], and their corresponding 95% confidence intervals (CIs) using a bivariate random-effect meta-analysis model (21). The summary receiver operator characteristic (SROC) curve was plotted, and the area under the SROC Area Under Curve (AUC) was calculated to evaluate the pooled diagnostic performance of CT in confirmed cases, CT in suspected cases for predicting COVID-19 diagnosis (22, 23). Heterogeneity between enrolled studies was quantified by the *I*^*2*^ statistic and assessed by the Cochran's Q-statistic. *I*^*2*^ = 0% indicated no heterogeneity, and *I*^*2*^ = 100% indicated maximal heterogeneity (24, 25). The assumption of heterogeneity was deemed valid for *I*^*2*^ > 50% and *P* < 0.10. Finally, in all analyses, the level of significance for the effect size estimation was set at 5%, and all tests were two-sided.

## 3. Results

### 3.1. Study selection

**Figure 1:**
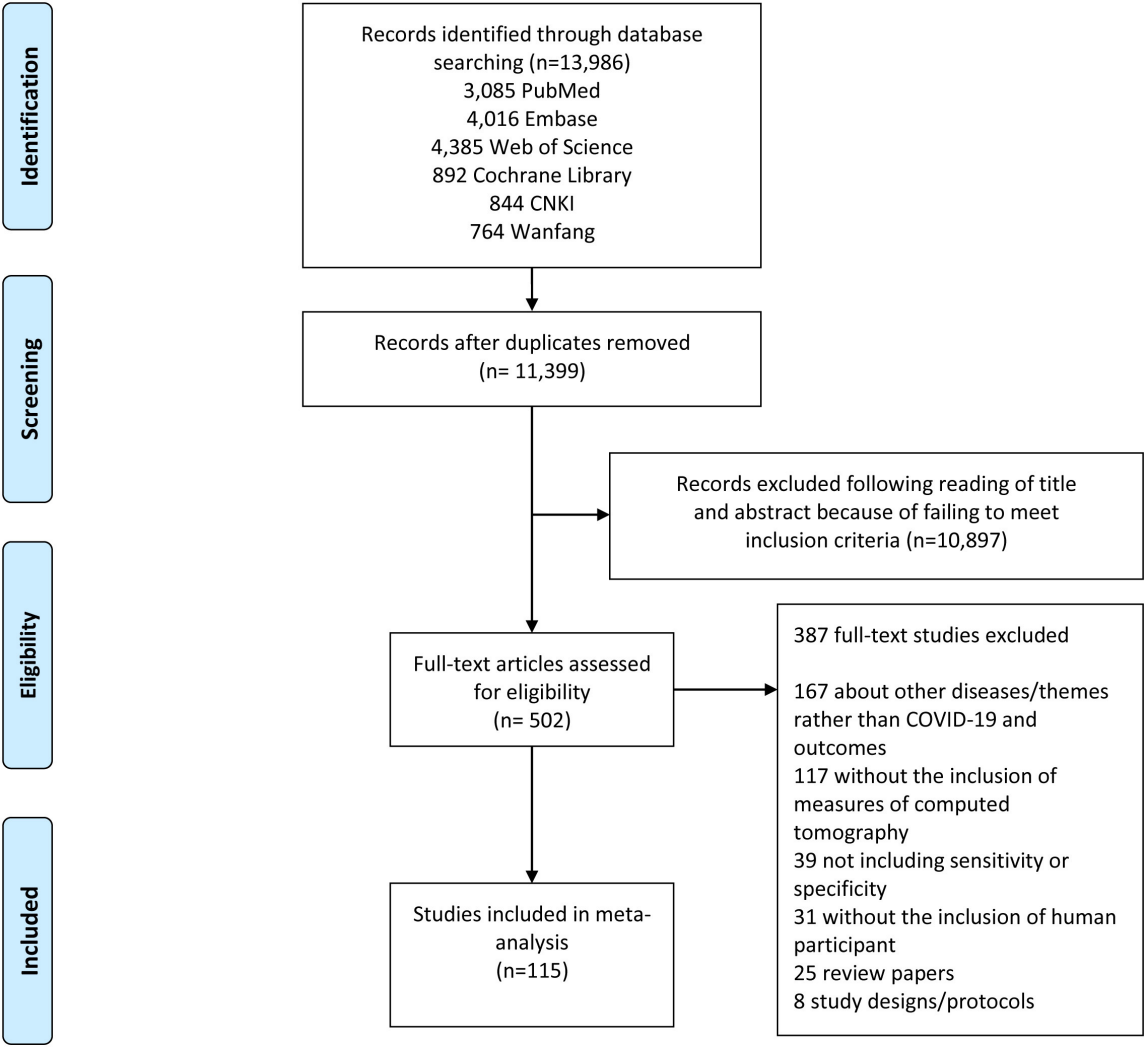
Study selection flow chart. A Preferred Reporting Items for Systematic Reviews and Meta-Analyses (PRISMA) flow chart demonstrating the selection process of articles included in the analysis as well as in the qualitative summary.

The search strategy retrieved a total of 13,986 studies, of which 3,085 were from PubMed, 4,016 were from Embase, 4,385 were from Web of Science, 892 were from Cochrane Library, 844 were from CNKI, and 764 were from Wanfang. Following a full review of 502 of these studies, 115 met the inclusion criteria for this meta-analysis. The PRISMA flowchart in Figure 1 shows the number of studies and the selection process.

### 3.2. Characteristics of eligible studies

Characteristics of the eligible studies are shown in Appendix 2. Further, focusing on test method, 115 studies reported on CT. The QUADAS-2 score of these studies varied between 1 and 7, with 42 studies of high quality, 58 of moderate quality, and 15 studies of low quality.

### 3.3. Main outcomes

**Figure 2:**
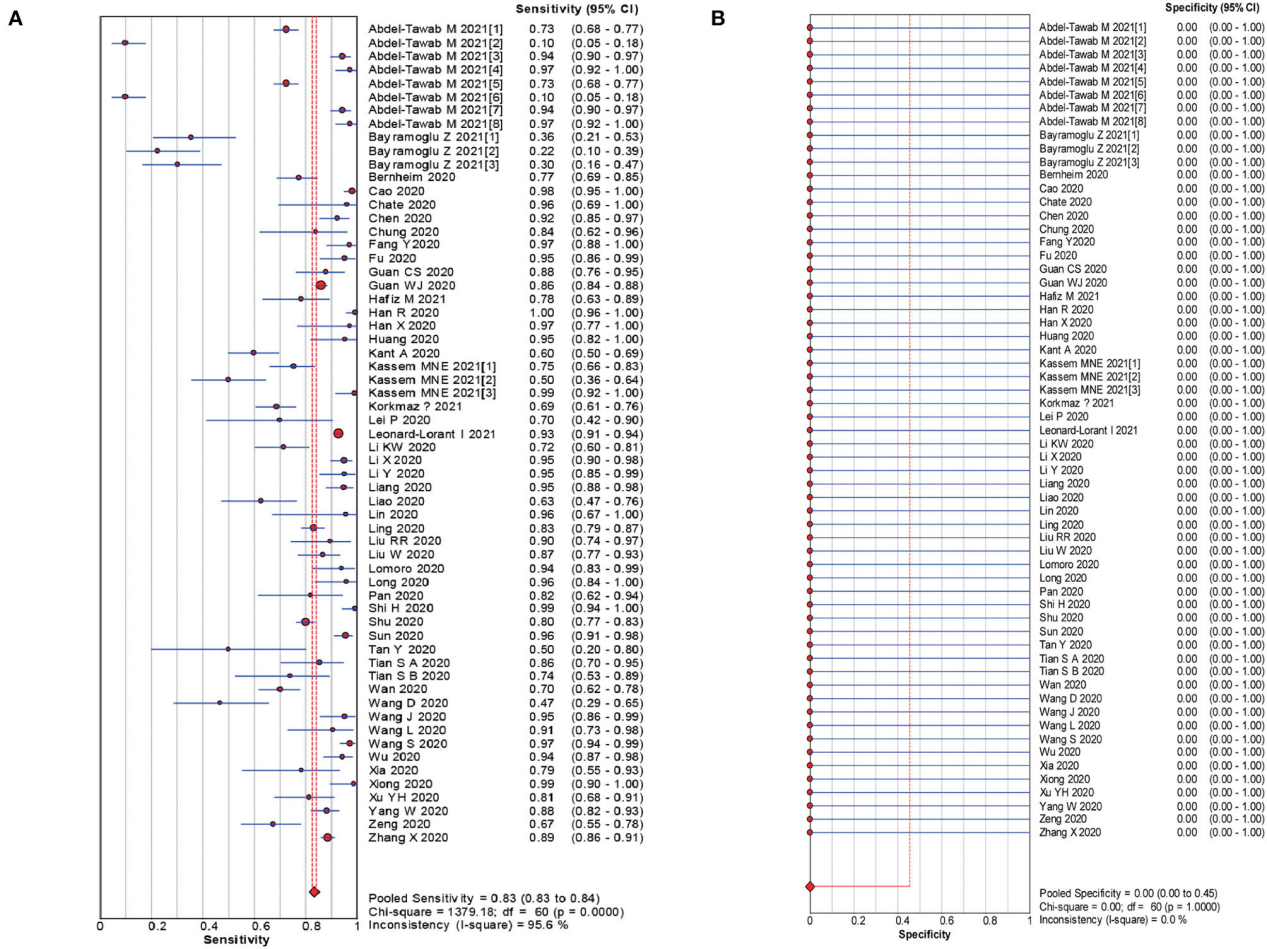
Forest plot of sensitivities **(A)** and specificities **(B)** of CT in confirmed cases for predicting COVID-19 diagnosis.

The pooled estimates for sensitivity and specificity of CT in confirmed cases to predicting COVID-19 diagnosis were 0.83 (95% CI: 0.83–0.84) and 0.00 (95% CI: 0.00–0.45), respectively (Figure 2), corresponding to a PLR of 1.50 (95% CI: 1.25–1.79) (Appendix 3) and an NLR of 0.28 (95% CI: 0.22–0.37) (Appendix 4). The overall AUC was 0.76 (Figure 4) and the dOR was 5.51 (95% CI: 3.78–8.02) (Figure 5).

**Figure 3:**
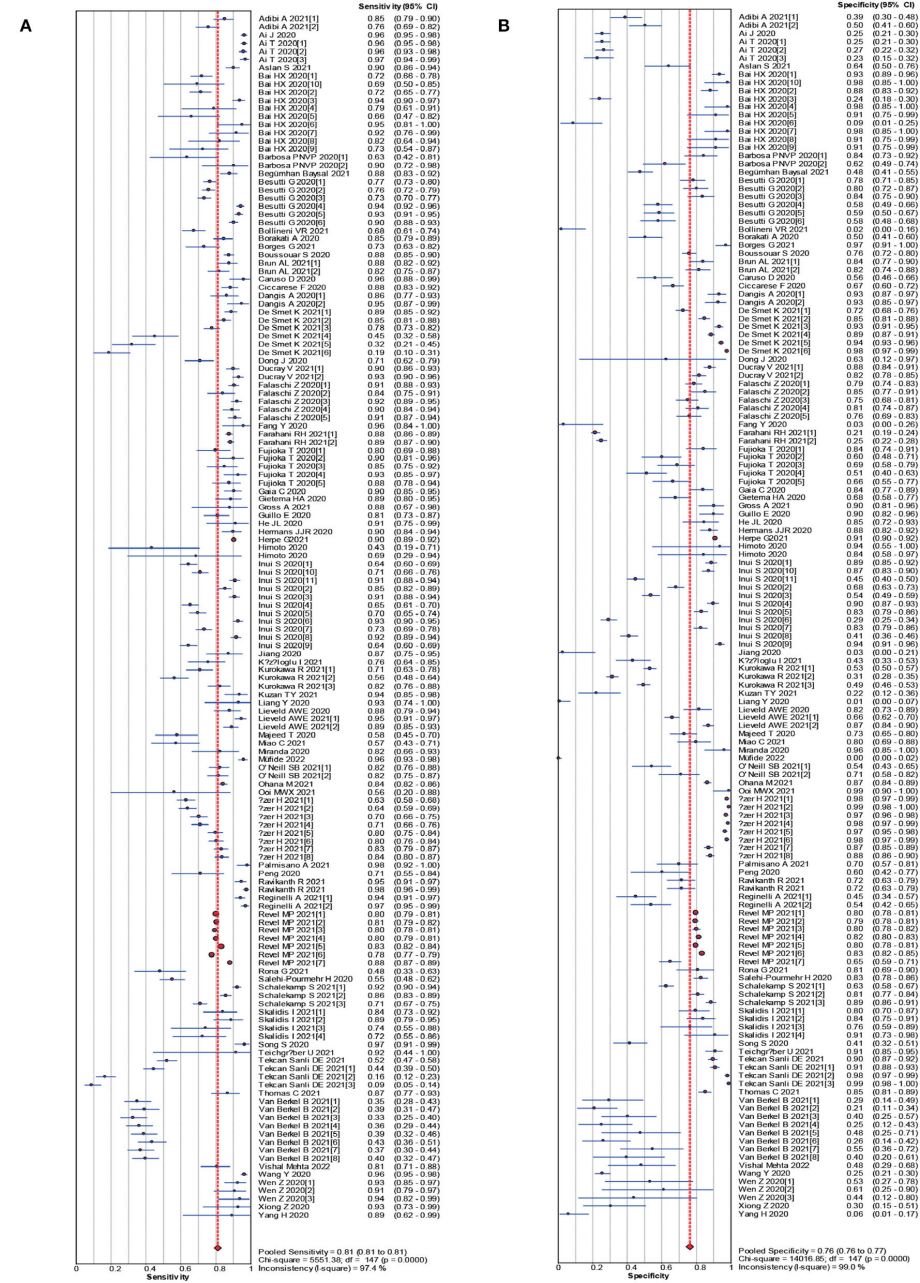
Forest plot of sensitivities **(A)** and specificities **(B)** of CT in suspected cases for predicting COVID-19 diagnosis.

**Figure 4:**
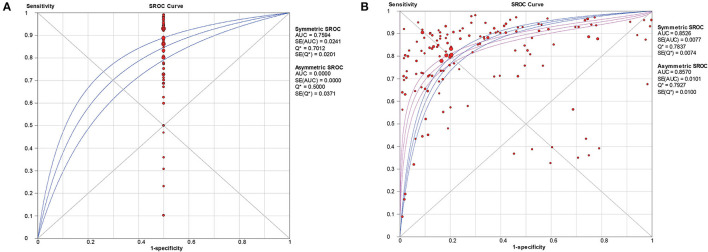
SROC curve with pooled estimates of sensitivity, specificity, and the AUC for all included studies of CT in confirmed cases **(A)**, CT in suspected cases **(B)** for predicting COVID-19 diagnosis. AUC, area under the SROC curve; SROC, summary receiver operator characteristic.

**Figure 5:**
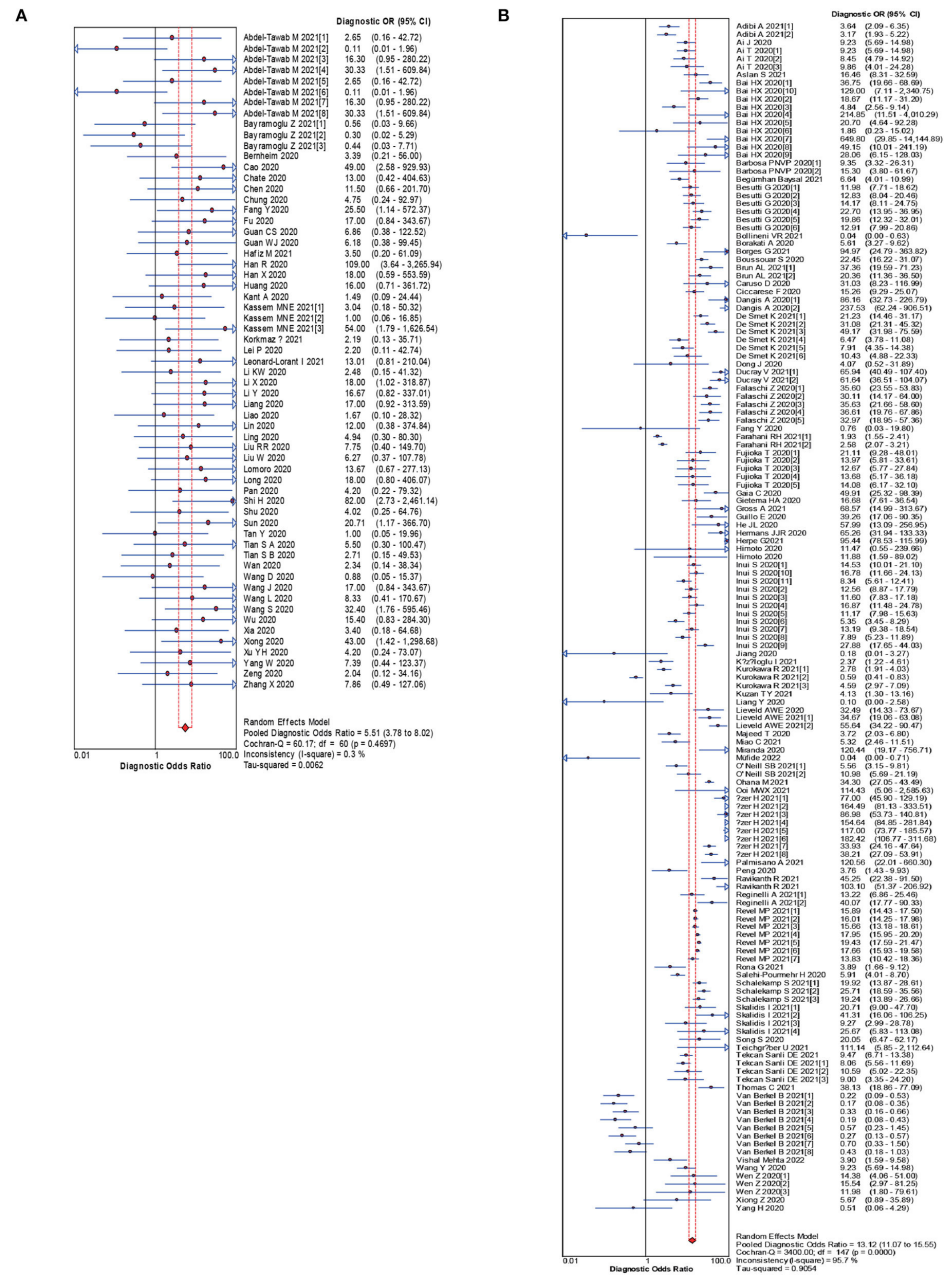
Forest plot of the pooled dOR of CT in confirmed cases **(A)**, CT in suspected cases **(B)** for predicting COVID-19 diagnosis.

The pooled estimates for sensitivity and specificity of CT in suspected cases to predicting COVID-19 diagnosis were 0.81 (95% CI: 0.81–0.81) and 0.76 (95% CI: 0.76–0.77), respectively (Figure 3), corresponding to a PLR of 3.28 (95% CI: 2.80–3.84) (Appendix 5) and an NLR of 0.27(95% CI: 0.24–0.30) (Appendix 6). The overall AUC was 0.85 (Figure 4) and the dOR was 13.12 (95% CI: 11.07–15.55) (Figure 5).

## 4. Discussion

There are several meta-analyses that discussed the relationship between CT and SARS-CoV-2 infection. But the number of studies included in previous meta-analyses was relatively limited. One hundred and fifteen studies were included with 51,500 participants in our meta-analysis. This is the maximum sample size, comprehensive, and updated review of the latest advances in the CT auxiliary screening of COVID-19 (10, 12, 26). In view of the relatively strong infectivity of COVID-19, early detection, reporting, isolation and treatment are of great significance for the prevention and control of the spread of the infection. Following their encouraging accuracy as shown in the reviewed studies, RT-PCR nucleic acid detection is recommended as the main screening criteria (27).

Current studies have shown that nucleic acid detection technology has the characteristics of early diagnosis, high sensitivity and specificity (28). However, in practice, this technology often yields false negatives. Therefore, the potential methods to reduce false negative results from the aspects of sample collection time, sample collection site and nucleic acid extraction process are worth discussing (10). First, mutations in the primers design area of the viral RNA may result in false negative test results. Thus, based on the preceding possibility, we recommend the following: collecting nasopharyngeal swabs within 3–7 days of onset (29), testing sputum samples in patients with negative RT-PCR results from pharyngeal swabs, and suspecting or confirming a high probability of infection (30).

**Figure 6:**
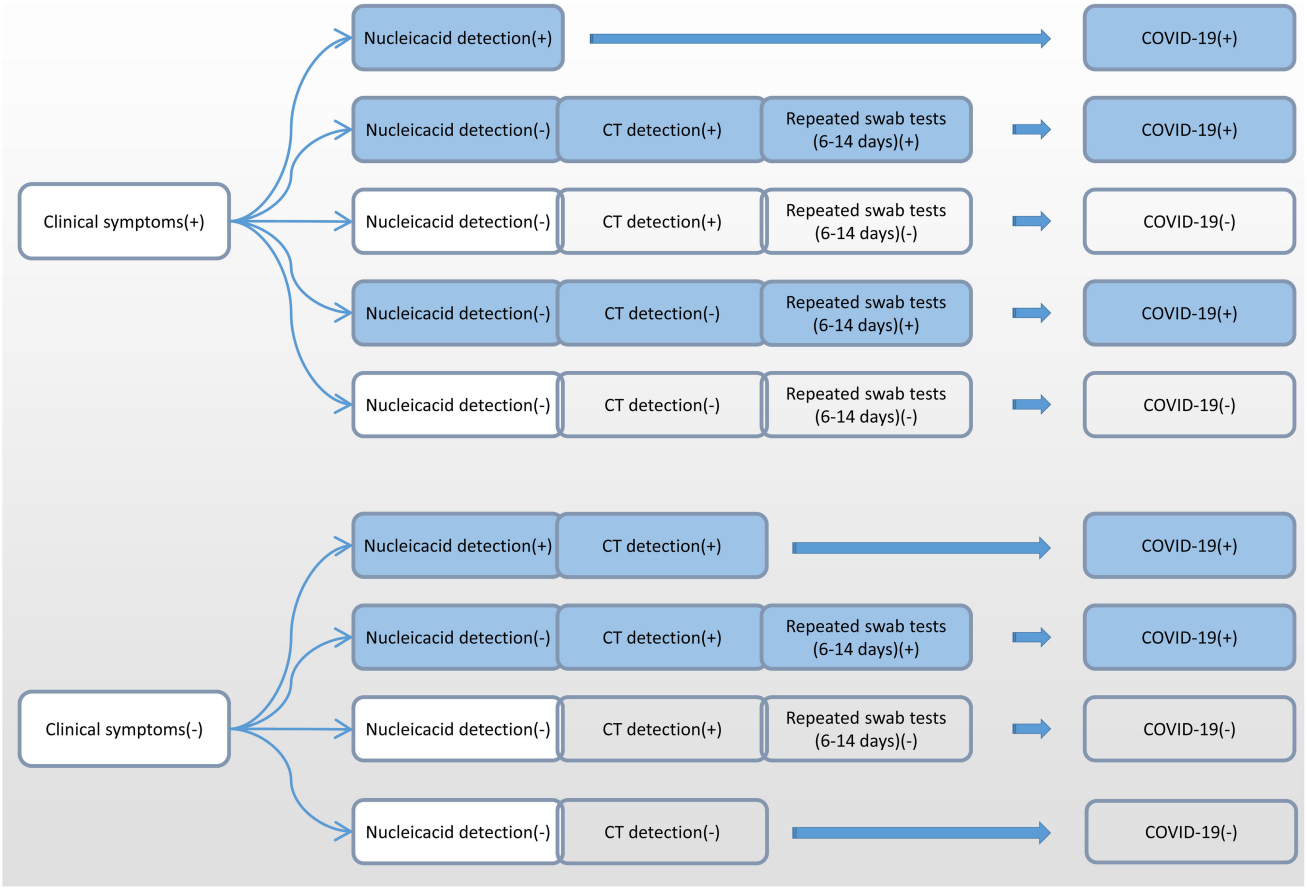
Flow chart of the selection of different detection methods for SARS-CoV-2 at different infection periods. SARS-CoV-2, severe acute respiratory syndrome coronavirus 2.

Due to the differences in the incubation period of individuals, we recommend multiple nucleic acid tests for clinically symptomatic patients in order to improve the detection rate (31). Alveolar lavage fluid sampling is not recommended because of the risk of trauma and cross-infection. According to the accurate selection of appropriate detection methods at different infection periods, we recommend nucleic acid detection and CT detection immediately after showing clinical symptoms (Figure 6). Six to fourteen days after the onset of clinical symptoms, repeated swab tests may be helpful for individuals with positive CT screening but negative RT-PCR screening.

Furthermore, the results suggested that CT scan can be used as a reliable method for screening COVID-19, especially in patients with a history of COVID-19 exposure, to facilitate early diagnosis and isolation of cases (32). The CT findings of COVID-19 and non-COVID-19 patients were significantly different with high specificity (33). Most of the patients with COVID-19 had bilateral pneumonia, and the CT findings were multiple spots and ground hyaline shadows (7). About 10 days after the initial onset of symptoms, chest CT showed the most severe lung abnormalities, and about 14 days after the initial onset of symptoms, chest CT began to improve (34). However, there are contradictions in the sensitivity of CT scan. Some studies have shown that chest CT has a high sensitivity to the screening of COVID-19 (32), while other studies have shown that chest CT has a moderate sensitivity to the screening of COVID-19 (35).

This study also has some limitations. First, there were heterogeneities in our meta-analyses, which might be originated from basic characteristics of our included studies, such as race, sex, age, smoking, and obesity. Due to the included studies were from different countries and above confounders could not be adjusted for, so our study was highly heterogeneous. Additionally, many patients may have no symptoms in the early stages of COVID-19 infection, these patients may lack CT data and the existing data may be scattered (36, 37). Finally, in rural areas of some developing countries, they are expensive because they are based on CT expensive equipment, hence their popularity is not high.

## 5. Conclusions

Multidisciplinary cooperation can improve the diagnostic efficiency of COVID-19. We recommend the use of RT-PCR nucleic acid as the main screening criteria. Through standard sampling, sample processing, combined CT and accurate selection of appropriate detection methods in different infection stages of laboratory methods, sensitivity of detection can be improved.

## Data availability statement

The original contributions presented in the study are included in the article/Supplementary material, further inquiries can be directed to the corresponding author.

## Author contributions

XP, PJ, and AL contributed to the study design, while YC and AK contributed to the data collection. Interpretation of results was performed by AL and YC, whereas XP, AL, and SW drafted the manuscript and edited the language. All authors participated in the critical revisions and approved the final version of the manuscript.
