# Supplementary material for: Auxiliary screening COVID-19 by computed tomography
Source: Front Public Health. 2023 Jun 5;11:974542. doi: 10.3389/fpubh.2023.974542 (PMC10278544; doi:10.3389/fpubh.2023.974542)

**Supplementary appendix**

**Contents**

**Appendix 1.** Search strategy and search results

**Appendix 2.** Characteristics and data of included studies for CT

**Appendix 3.** Forest plot of likelihood ratios for positive test results of CT in confirmed cases for predicting COVID-19 diagnosis

**Appendix 4.** Forest plot of likelihood ratios for negative test results of CT in confirmed cases for predicting COVID-19 diagnosis

**Appendix 5.** Forest plot of likelihood ratios for positive test results of CT in suspected cases for predicting COVID-19 diagnosis

**Appendix 6.** Forest plot of likelihood ratios for negative test results of CT in suspected cases for predicting COVID-19 diagnosis

**Appendix 1. Search strategy and search results**

The search strategy includes all possible combinations of keywords in the title/abstract from the following two groups:

(#1)“COVID-19” or “2019 novel coronavirus infection” or “COVID19” or “coronavirus disease 2019” or “coronavirus disease-19” or “2019-nCoV disease” or “2019 novel coronavirus disease” or “2019-nCoV infection” or “coronavirus disease 2019 virus” or “SARS-CoV-2” or “SARS2” or “2019-nCoV” or “2019 novel coronavirus” or “severe acute respiratory syndrome coronavirus 2” or “COVID Asymptomatic Infections”

(#2) “Nucleic Acid Detection” or “Nucleic Acid Probes” or “Nucleic Acid Probes” or “Reagent Kits, Diagnostic” or “Reagent Strips” or “polymerase chain reaction” or “PCR*”

(#3) “computed tomography” or “CT”

(#1 AND #2 AND #3)

**The search results as following:**

Cochrane library

| #1 | “COVID 19” or “2019 novel corona virus infection” or “COVID19” or “corona virus disease 2019” or “corona virus disease 19” or “2019 nCoV disease” or “2019 novel corona virus disease” or “2019 nCoV infection” or “corona virus disease 2019 virus” or “SARS CoV 2” or “SARS2” or “2019 nCoV” or “2019 novel corona virus” or “severe acute respiratory syndrome corona virus 2” or “COVID, Asymptomatic Infections” |
| --- | --- |
| #2 | “Nucleic Acid Detection” or “Nucleic Acid Probes” or “Molecular Probes” or “Nucleic Acid Probes” or “Reagent Kits, Diagnostic” or “Reagent Strips” or “polymerase chain reaction” or “PCR*” |
| #3 | “computed tomography” or “CT” |
|  | #1 AND #2 AND #3 |

Web of science

| #1 | TS= (“COVID 19” or “2019 novel corona virus infection” or “COVID19” or “corona virus disease 2019” or “corona virus disease 19” or “2019 nCoV disease” or “2019 novel corona virus disease” or “2019 nCoV infection” or “corona virus disease 2019 virus” or “SARS CoV 2” or “SARS2” or “2019 nCoV” or “2019 novel corona virus” or “severe acute respiratory syndrome corona virus 2” or “COVID, Asymptomatic Infections”) |
| --- | --- |
| #2 | TS= (“Nucleic Acid Detection” or “Nucleic Acid Probes” or “Molecular Probes” or “Nucleic Acid Probes” or “Reagent Kits, Diagnostic” or “Reagent Strips” or “polymerase chain reaction” or “PCR*” |
| #3 | TS= (“computed tomography” or “CT” ) |
|  | #1 AND #2 AND #3 |

**Pubmed**

| #1 | “COVID-19”[Title/Abstract] OR “2019 novel coronavirus infection”[Title/Abstract] OR “COVID19”[Title/Abstract] OR “coronavirus disease 2019”[Title/Abstract] OR “coronavirus disease-19”[Title/Abstract] OR “2019-nCoV disease”[Title/Abstract] OR “2019 novel coronavirus disease”[Title/Abstract] OR “2019-nCoV infection”[Title/Abstract] OR “coronavirus disease 2019 virus”[Title/Abstract] OR “SARS-CoV-2”[Title/Abstract] OR “SARS2”[Title/Abstract] OR “2019-nCoV”[Title/Abstract] OR “2019 novel coronavirus”[Title/Abstract] OR “severe acute respiratory syndrome coronavirus 2”[Title/Abstract] OR “COVID Asymptomatic Infections”[Title/Abstract] |
| --- | --- |
| #2 | “Nucleic Acid Detection”[Title/Abstract] OR “Nucleic Acid Probes”[Title/Abstract] OR “Molecular Probes”[Title/Abstract] OR “Nucleic Acid Probes”[Title/Abstract] OR “Reagent Kits, Diagnostic”[Title/Abstract] OR “Reagent Strips”[Title/Abstract] OR “polymerase chain reaction”[Title/Abstract] OR “PCR*”[Title/Abstract] |
| #3 | “computed tomography”[Title/Abstract] OR “CT”[Title/Abstract] |
|  | #1 AND #2 AND #3 |

**Embase**

| #1 | “COVID 19” or “2019 novel corona virus infection” or “COVID19” or “corona virus disease 2019” or “corona virus disease 19” or “2019 nCoV disease” or “2019 novel corona virus disease” or “2019 nCoV infection” or “corona virus disease 2019 virus” or “SARS CoV 2” or “SARS2” or “2019 nCoV” or “2019 novel corona virus” or “severe acute respiratory syndrome corona virus 2” or “COVID, Asymptomatic Infections” |
| --- | --- |
| #2 | “Nucleic Acid Detection” or “Nucleic Acid Probes” or “Acid Probes Nucleic” or “Probes Nucleic Acid” or “Molecular Probes” or “Nucleic Acid Probes” or “Reagent Kits, Diagnostic” or “Reagent Strips” or “polymerase chain reaction” or “PCR” |
| #3 | “computed tomography” or “CT” |
|  | #1 AND #2 AND #3 |

CNKI

| #1 | SU =“COVID-19” + “2019新型冠状病毒” + “COVID 19” + “2019冠状病毒” + “冠状病毒病-19” + “2019 nCoV病” + “2019 nCoV感染” + “2019冠状病毒病病毒” + “SARS-CoV-2” + “SARS2” + “2019 nCoV” + “2019新型冠状病毒” + “严重急性呼吸综合征冠状病毒2” + “COVID 19, 无症状感染者” + “COVID 19, 隐匿感染者” + “COVID 19, 隐藏感染者” + “COVID 19, 隐性感染者” |
| --- | --- |
| #2 | SU = "核酸检测" + "核酸探针" +"分子探针" + "诊断试剂盒" + "聚合酶链式反应" + "PCR" |
| #3 | SU = "计算机断层扫描" + "CT" |
|  | #1 AND #2 AND #3 |

Wanfang

| #1 | 主题: “COVID-19”or“2019新型冠状病毒”or“COVID 19”or“2019冠状病毒”or“冠状病毒病-19”or“2019 nCoV病”or “2019 nCoV感染”or “2019冠状病毒病病毒”or“SARS-CoV-2”or“SARS2”or“2019 nCoV”or“2019新型冠状病毒”or“严重急性呼吸综合征冠状病毒2” or“COVID 19, 无症状感染者” or“COVID 19, 隐匿感染者”or“COVID 19, 隐藏感染者”or“COVID 19, 隐性感染者” |
| --- | --- |
| #2 | 主题: "核酸检测" or "核酸探针" or "分子探针" or "诊断试剂盒" or "聚合酶链式反应" or "PCR" |
| #3 | 主题: "计算机断层扫描" or "CT" |
|  | #1 AND #2 AND #3 |

387 full-text studies excluded

167 about other diseases/themes rather than COVID-19 and outcomes【^1-167^】

117 without the inclusion of measures of computed tomography【^168-284^】

39 not including sensitivity or specificity【^285-323^】

31 without the inclusion of human participant【^324-354^】

25 review papers【^355-379^】

8 study designs/protocols【^380-387^】

**Appendix 2. Characteristics and data of included studies for CT**

| **Ref** | **Study** | **Test Method** | **TP** | **FP** | **FN** | **TN** | **QUADAS-2** |
| --- | --- | --- | --- | --- | --- | --- | --- |
| 388 | Abdel-Tawab M 2021[1] | CT for confirmed cases | 261 | 0 | 98 | 0 | 5 |
| 388 | Abdel-Tawab M 2021[2] | CT for confirmed cases | 9 | 0 | 87 | 0 | 5 |
| 388 | Abdel-Tawab M 2021[3] | CT for confirmed cases | 162 | 0 | 9 | 0 | 5 |
| 388 | Abdel-Tawab M 2021[4] | CT for confirmed cases | 90 | 0 | 2 | 0 | 5 |
| 388 | Abdel-Tawab M 2021[5] | CT for confirmed cases | 261 | 0 | 98 | 0 | 5 |
| 388 | Abdel-Tawab M 2021[6] | CT for confirmed cases | 9 | 0 | 87 | 0 | 5 |
| 388 | Abdel-Tawab M 2021[7] | CT for confirmed cases | 162 | 0 | 9 | 0 | 5 |
| 388 | Abdel-Tawab M 2021[8] | CT for confirmed cases | 90 | 0 | 2 | 0 | 5 |
| 389 | Bayramoglu Z 2021[1] | CT for confirmed cases | 13 | 0 | 24 | 0 | 3 |
| 389 | Bayramoglu Z 2021[2] | CT for confirmed cases | 8 | 0 | 29 | 0 | 3 |
| 389 | Bayramoglu Z 2021[3] | CT for confirmed cases | 11 | 0 | 26 | 0 | 3 |
| 390 | Bernheim 2020 | CT for confirmed cases | 94 | 0 | 27 | 0 | 7 |
| 391 | Cao 2020 | CT for confirmed cases | 195 | 0 | 3 | 0 | 4 |
| 392 | Chate 2020 | CT for confirmed cases | 12 | 0 | 0 | 0 | 4 |
| 393 | Chen 2020 | CT for confirmed cases | 91 | 0 | 7 | 0 | 4 |
| 394 | Chung 2020 | CT for confirmed cases | 18 | 0 | 3 | 0 | 4 |
| 395 | Fang Y2020 | CT for confirmed cases | 50 | 0 | 1 | 0 | 4 |
| 396 | Fu 2020 | CT for confirmed cases | 50 | 0 | 2 | 0 | 4 |
| 397 | Guan CS 2020 | CT for confirmed cases | 47 | 0 | 6 | 0 | 7 |
| 398 | Guan WJ 2020 | CT for confirmed cases | 840 | 0 | 135 | 0 | 4 |
| 399 | Hafiz M 2021 | CT for confirmed cases | 34 | 0 | 9 | 0 | 3 |
| 400 | Han R 2020 | CT for confirmed cases | 108 | 0 | 0 | 0 | 4 |
| 401 | Han X 2020 | CT for confirmed cases | 17 | 0 | 0 | 0 | 4 |
| 402 | Himoto 2020 | CT for suspected cases | 6 | 0 | 8 | 7 | 4 |
| 403 | Huang 2020 | CT for confirmed cases | 31 | 0 | 1 | 0 | 6 |
| 404 | Kant A 2020 | CT for confirmed cases | 63 | 0 | 42 | 0 | 2 |
| 405 | Kassem MNE 2021[1] | CT for confirmed cases | 78 | 0 | 25 | 0 | 3 |
| 405 | Kassem MNE 2021[2] | CT for confirmed cases | 25 | 0 | 25 | 0 | 3 |
| 405 | Kassem MNE 2021[3] | CT for confirmed cases | 53 | 0 | 0 | 0 | 3 |
| 406 | Korkmaz İ 2021 | CT for confirmed cases | 104 | 0 | 47 | 0 | 4 |
| 407 | Lei P 2020 | CT for confirmed cases | 10 | 0 | 4 | 0 | 4 |
| 408 | Leonard-Lorant I 2021 | CT for confirmed cases | 1014 | 0 | 77 | 0 | 4 |
| 409 | Li KW 2020 | CT for confirmed cases | 56 | 0 | 22 | 0 | 4 |
| 410 | Li X 2020 | CT for confirmed cases | 125 | 0 | 6 | 0 | 6 |
| 411 | Li Y 2020 | CT for confirmed cases | 49 | 0 | 2 | 0 | 4 |
| 412 | Liang 2020 | CT for confirmed cases | 84 | 0 | 4 | 0 | 4 |
| 413 | Liao 2020 | CT for confirmed cases | 29 | 0 | 17 | 0 | 6 |
| 414 | Lin 2020 | CT for confirmed cases | 11 | 0 | 0 | 0 | 4 |
| 415 | Ling 2020 | CT for confirmed cases | 246 | 0 | 49 | 0 | 6 |
| 416 | Liu RR 2020 | CT for confirmed cases | 30 | 0 | 3 | 0 | 6 |
| 417 | Liu W 2020 | CT for confirmed cases | 68 | 0 | 10 | 0 | 4 |
| 418 | Lomoro 2020 | CT for confirmed cases | 40 | 0 | 2 | 0 | 4 |
| 419 | Long 2020 | CT for confirmed cases | 35 | 0 | 1 | 0 | 4 |
| 420 | Pan 2020 | CT for confirmed cases | 20 | 0 | 4 | 0 | 4 |
| 421 | Ravikanth R 2021 | CT for suspected cases | 206 | 37 | 11 | 94 | 5 |
| 422 | Shi H 2020 | CT for confirmed cases | 81 | 0 | 0 | 0 | 4 |
| 423 | Shu 2020 | CT for confirmed cases | 437 | 0 | 108 | 0 | 6 |
| 424 | Sun 2020 | CT for confirmed cases | 144 | 0 | 6 | 0 | 4 |
| 425 | Tan Y 2020 | CT for confirmed cases | 5 | 0 | 5 | 0 | 4 |
| 426 | Tekcan Sanli DE 2021[1] | CT for suspected cases | 127 | 50 | 159 | 509 | 3 |
| 426 | Tekcan Sanli DE 2021[2] | CT for suspected cases | 31 | 9 | 159 | 509 | 3 |
| 426 | Tekcan Sanli DE 2021[3] | CT for suspected cases | 15 | 5 | 159 | 509 | 3 |
| 427 | Tian S A 2020 | CT for confirmed cases | 32 | 0 | 5 | 0 | 4 |
| 428 | Tian S B 2020 | CT for confirmed cases | 18 | 0 | 6 | 0 | 5 |
| 429 | Wan 2020 | CT for confirmed cases | 95 | 0 | 40 | 0 | 6 |
| 430 | Wang D 2020 | CT for confirmed cases | 14 | 0 | 16 | 0 | 5 |
| 431 | Wang J 2020 | CT for confirmed cases | 50 | 0 | 2 | 0 | 6 |
| 432 | Wang L 2020 | CT for confirmed cases | 24 | 0 | 2 | 0 | 5 |
| 433 | Wang S 2020 | CT for confirmed cases | 161 | 0 | 4 | 0 | 5 |
| 434 | Wu 2020 | CT for confirmed cases | 76 | 0 | 4 | 0 | 5 |
| 435 | Xia 2020 | CT for confirmed cases | 16 | 0 | 4 | 0 | 5 |
| 436 | Xiong 2020 | CT for confirmed cases | 42 | 0 | 0 | 0 | 5 |
| 437 | Xu YH 2020 | CT for confirmed cases | 41 | 0 | 9 | 0 | 5 |
| 438 | Yang W 2020 | CT for confirmed cases | 132 | 0 | 17 | 0 | 5 |
| 439 | Zeng 2020 | CT for confirmed cases | 46 | 0 | 22 | 0 | 6 |
| 440 | Zhang X 2020 | CT for confirmed cases | 573 | 0 | 72 | 0 | 5 |
| 441 | Adibi A 2021[1] | CT for suspected cases | 145 | 74 | 25 | 47 | 6 |
| 441 | Adibi A 2021[2] | CT for suspected cases | 129 | 60 | 41 | 61 | 6 |
| 442 | Ai J 2020 | CT for suspected cases | 580 | 308 | 21 | 105 | 7 |
| 443 | Ai T 2020[1] | CT for suspected cases | 580 | 308 | 21 | 105 | 4 |
| 443 | Ai T 2020[2] | CT for suspected cases | 362 | 225 | 15 | 81 | 4 |
| 443 | Ai T 2020[3] | CT for suspected cases | 218 | 83 | 6 | 24 | 4 |
| 444 | Aslan S 2021 | CT for suspected cases | 226 | 20 | 24 | 36 | 4 |
| 445 | Bai HX 2020[1] | CT for suspected cases | 158 | 13 | 61 | 192 | 6 |
| 445 | Bai HX 2020[2] | CT for suspected cases | 157 | 24 | 62 | 181 | 6 |
| 445 | Bai HX 2020[3] | CT for suspected cases | 206 | 156 | 13 | 49 | 6 |
| 445 | Bai HX 2020[4] | CT for suspected cases | 24 | 0 | 6 | 28 | 6 |
| 445 | Bai HX 2020[5] | CT for suspected cases | 20 | 2 | 10 | 26 | 6 |
| 445 | Bai HX 2020[6] | CT for suspected cases | 29 | 26 | 1 | 2 | 6 |
| 445 | Bai HX 2020[7] | CT for suspected cases | 28 | 0 | 2 | 28 | 6 |
| 445 | Bai HX 2020[8] | CT for suspected cases | 25 | 2 | 5 | 26 | 6 |
| 445 | Bai HX 2020[9] | CT for suspected cases | 22 | 2 | 8 | 26 | 6 |
| 445 | Bai HX 2020[10] | CT for suspected cases | 21 | 0 | 9 | 28 | 6 |
| 446 | Barbosa PNVP 2020[1] | CT for suspected cases | 16 | 10 | 9 | 56 | 5 |
| 446 | Barbosa PNVP 2020[2] | CT for suspected cases | 23 | 25 | 2 | 41 | 5 |
| 447 | Begümhan Baysal 2021 | CT for suspected cases | 179 | 106 | 24 | 96 | 4 |
| 448 | Besutti G 2020[1] | CT for suspected cases | 423 | 31 | 128 | 114 | 7 |
| 448 | Besutti G 2020[2] | CT for suspected cases | 428 | 26 | 135 | 107 | 7 |
| 448 | Besutti G 2020[3] | CT for suspected cases | 438 | 16 | 158 | 84 | 7 |
| 448 | Besutti G 2020[4] | CT for suspected cases | 520 | 61 | 31 | 84 | 7 |
| 448 | Besutti G 2020[5] | CT for suspected cases | 526 | 55 | 37 | 78 | 7 |
| 448 | Besutti G 2020[6] | CT for suspected cases | 539 | 42 | 57 | 58 | 7 |
| 449 | Bollineni VR 2021 | CT for suspected cases | 144 | 27 | 69 | 0 | 7 |
| 450 | Borakati A 2020 | CT for suspected cases | 162 | 55 | 29 | 56 | 6 |
| 451 | Borges G 2021 | CT for suspected cases | 64 | 2 | 23 | 86 | 4 |
| 452 | Boussouar S 2020 | CT for suspected cases | 479 | 124 | 67 | 393 | 4 |
| 453 | Brun AL 2021[1] | CT for suspected cases | 153 | 21 | 21 | 112 | 3 |
| 453 | Brun AL 2021[2] | CT for suspected cases | 143 | 24 | 31 | 109 | 3 |
| 454 | Caruso D 2020 | CT for suspected cases | 60 | 42 | 2 | 54 | 6 |
| 455 | Ciccarese F 2020 | CT for suspected cases | 187 | 83 | 24 | 166 | 6 |
| 456 | Dangis A 2020[1] | CT for suspected cases | 72 | 7 | 11 | 102 | 6 |
| 456 | Dangis A 2020[2] | CT for suspected cases | 65 | 6 | 3 | 82 | 6 |
| 457 | De Smet K 2021[1] | CT for suspected cases | 319 | 138 | 39 | 363 | 7 |
| 457 | De Smet K 2021[2] | CT for suspected cases | 304 | 76 | 54 | 425 | 7 |
| 457 | De Smet K 2021[3] | CT for suspected cases | 279 | 33 | 79 | 468 | 7 |
| 457 | De Smet K 2021[4] | CT for suspected cases | 27 | 121 | 33 | 957 | 7 |
| 457 | De Smet K 2021[5] | CT for suspected cases | 19 | 60 | 41 | 1018 | 7 |
| 457 | De Smet K 2021[6] | CT for suspected cases | 11 | 23 | 49 | 1055 | 7 |
| 458 | Dong J 2020 | CT for suspected cases | 91 | 1 | 37 | 2 | 7 |
| 459 | Ducray V 2021[1] | CT for suspected cases | 259 | 49 | 28 | 358 | 6 |
| 459 | Ducray V 2021[2] | CT for suspected cases | 268 | 74 | 19 | 333 | 6 |
| 460 | Falaschi Z 2020[1] | CT for suspected cases | 419 | 66 | 43 | 245 | 6 |
| 460 | Falaschi Z 2020[2] | CT for suspected cases | 81 | 16 | 15 | 94 | 6 |
| 460 | Falaschi Z 2020[3] | CT for suspected cases | 338 | 50 | 28 | 151 | 6 |
| 460 | Falaschi Z 2020[4] | CT for suspected cases | 166 | 29 | 19 | 126 | 6 |
| 460 | Falaschi Z 2020[5] | CT for suspected cases | 253 | 37 | 24 | 119 | 6 |
| 395 | Fang Y 2020 | CT for suspected cases | 35 | 15 | 1 | 0 | 4 |
| 461 | Farahani RH 2021[1] | CT for suspected cases | 1077 | 882 | 152 | 241 | 3 |
| 461 | Farahani RH 2021[2] | CT for suspected cases | 1276 | 683 | 165 | 228 | 3 |
| 462 | Fujioka T 2020[1] | CT for suspected cases | 61 | 12 | 15 | 66 | 4 |
| 462 | Fujioka T 2020[2] | CT for suspected cases | 69 | 31 | 7 | 47 | 4 |
| 462 | Fujioka T 2020[3] | CT for suspected cases | 65 | 24 | 11 | 54 | 4 |
| 462 | Fujioka T 2020[4] | CT for suspected cases | 71 | 38 | 5 | 40 | 4 |
| 462 | Fujioka T 2020[5] | CT for suspected cases | 67 | 26 | 9 | 52 | 4 |
| 463 | Gaia C 2020 | CT for suspected cases | 147 | 24 | 15 | 128 | 6 |
| 464 | Gietema HA 2020 | CT for suspected cases | 74 | 35 | 9 | 75 | 7 |
| 465 | Gross A 2021 | CT for suspected cases | 18 | 7 | 2 | 69 | 5 |
| 466 | Guillo E 2020 | CT for suspected cases | 105 | 8 | 24 | 77 | 6 |
| 467 | He JL 2020 | CT for suspected cases | 26 | 8 | 2 | 46 | 6 |
| 468 | Hermans JJR 2020 | CT for suspected cases | 120 | 22 | 13 | 164 | 3 |
| 469 | Herpe G2021 | CT for suspected cases | 2320 | 204 | 244 | 2056 | 6 |
| 402 | Himoto 2020 | CT for suspected cases | 5 | 2 | 2 | 13 | 4 |
| 470 | Inui S 2020[1] | CT for suspected cases | 258 | 44 | 142 | 356 | 6 |
| 470 | Inui S 2020[2] | CT for suspected cases | 342 | 127 | 58 | 273 | 6 |
| 470 | Inui S 2020[3] | CT for suspected cases | 364 | 185 | 36 | 215 | 6 |
| 470 | Inui S 2020[4] | CT for suspected cases | 262 | 40 | 138 | 360 | 6 |
| 470 | Inui S 2020[5] | CT for suspected cases | 279 | 68 | 121 | 332 | 6 |
| 470 | Inui S 2020[6] | CT for suspected cases | 372 | 284 | 28 | 116 | 6 |
| 470 | Inui S 2020[7] | CT for suspected cases | 294 | 69 | 106 | 331 | 6 |
| 470 | Inui S 2020[8] | CT for suspected cases | 368 | 236 | 32 | 164 | 6 |
| 470 | Inui S 2020[9] | CT for suspected cases | 258 | 24 | 142 | 376 | 6 |
| 470 | Inui S 2020[10] | CT for suspected cases | 285 | 51 | 115 | 349 | 6 |
| 470 | Inui S 2020[11] | CT for suspected cases | 365 | 221 | 35 | 179 | 6 |
| 471 | Jiang 2020 | CT for suspected cases | 44 | 19 | 6 | 0 | 4 |
| 472 | Kızıloglu I 2021 | CT for suspected cases | 54 | 58 | 17 | 44 | 3 |
| 473 | Kurokawa R 2021[1] | CT for suspected cases | 111 | 360 | 45 | 408 | 5 |
| 473 | Kurokawa R 2021[2] | CT for suspected cases | 88 | 528 | 68 | 240 | 5 |
| 473 | Kurokawa R 2021[3] | CT for suspected cases | 129 | 389 | 27 | 379 | 5 |
| 474 | Kuzan TY 2021 | CT for suspected cases | 65 | 40 | 4 | 11 | 6 |
| 412 | Liang Y 2020 | CT for suspected cases | 20 | 67 | 1 | 0 | 4 |
| 475 | Lieveld AWE 2020 | CT for suspected cases | 76 | 18 | 10 | 82 | 5 |
| 476 | Lieveld AWE 2021[1] | CT for suspected cases | 223 | 172 | 12 | 334 | 3 |
| 476 | Lieveld AWE 2021[2] | CT for suspected cases | 210 | 65 | 25 | 441 | 3 |
| 477 | Majeed T 2020 | CT for suspected cases | 40 | 37 | 29 | 101 | 3 |
| 478 | Miao C 2021 | CT for suspected cases | 31 | 15 | 23 | 61 | 3 |
| 479 | Miranda 2020 | CT for suspected cases | 30 | 1 | 6 | 38 | 6 |
| 480 | Müfide 2022 | CT for suspected cases | 273 | 285 | 11 | 0 | 5 |
| 481 | O' Neill SB 2021[1] | CT for suspected cases | 157 | 38 | 33 | 45 | 6 |
| 481 | O' Neill SB 2021[2] | CT for suspected cases | 149 | 18 | 33 | 45 | 6 |
| 482 | Ohana M 2021 | CT for suspected cases | 919 | 148 | 172 | 955 | 4 |
| 483 | Ooi MWX 2021 | CT for suspected cases | 4 | 0 | 3 | 44 | 1 |
| 484 | Özer H 2021[1] | CT for suspected cases | 239 | 17 | 140 | 790 | 6 |
| 484 | Özer H 2021[2] | CT for suspected cases | 248 | 8 | 140 | 790 | 6 |
| 484 | Özer H 2021[3] | CT for suspected cases | 267 | 21 | 112 | 786 | 6 |
| 484 | Özer H 2021[4] | CT for suspected cases | 276 | 12 | 112 | 786 | 6 |
| 484 | Özer H 2021[5] | CT for suspected cases | 303 | 26 | 76 | 781 | 6 |
| 484 | Özer H 2021[6] | CT for suspected cases | 312 | 17 | 76 | 781 | 6 |
| 484 | Özer H 2021[7] | CT for suspected cases | 316 | 103 | 63 | 704 | 6 |
| 484 | Özer H 2021[8] | CT for suspected cases | 325 | 94 | 63 | 704 | 6 |
| 485 | Palmisano A 2021 | CT for suspected cases | 77 | 19 | 1 | 45 | 4 |
| 486 | Peng 2020 | CT for suspected cases | 28 | 13 | 11 | 20 | 6 |
| 487 | Ravikanth R 2021 | CT for suspected cases | 470 | 37 | 11 | 94 | 5 |
| 488 | Reginelli A 2021[1] | CT for suspected cases | 283 | 43 | 17 | 35 | 5 |
| 488 | Reginelli A 2021[2] | CT for suspected cases | 292 | 36 | 8 | 42 | 5 |
| 489 | Revel MP 2021[1] | CT for suspected cases | 5174 | 872 | 1274 | 3415 | 5 |
| 489 | Revel MP 2021[2] | CT for suspected cases | 3536 | 611 | 853 | 2362 | 5 |
| 489 | Revel MP 2021[3] | CT for suspected cases | 1638 | 261 | 421 | 1053 | 5 |
| 489 | Revel MP 2021[4] | CT for suspected cases | 3395 | 573 | 834 | 2529 | 5 |
| 489 | Revel MP 2021[5] | CT for suspected cases | 5174 | 872 | 1042 | 3415 | 5 |
| 489 | Revel MP 2021[6] | CT for suspected cases | 4477 | 679 | 1274 | 3415 | 5 |
| 489 | Revel MP 2021[7] | CT for suspected cases | 2550 | 87 | 342 | 162 | 5 |
| 490 | Rona G 2021 | CT for suspected cases | 23 | 11 | 25 | 48 | 3 |
| 491 | Salehi-Pourmehr H 2020 | CT for suspected cases | 111 | 63 | 90 | 304 | 7 |
| 492 | Schalekamp S 2021[1] | CT for suspected cases | 495 | 200 | 41 | 334 | 4 |
| 492 | Schalekamp S 2021[2] | CT for suspected cases | 460 | 101 | 76 | 433 | 4 |
| 492 | Schalekamp S 2021[3] | CT for suspected cases | 381 | 60 | 155 | 474 | 4 |
| 493 | Skalidis I 2021[1] | CT for suspected cases | 55 | 18 | 10 | 72 | 6 |
| 493 | Skalidis I 2021[2] | CT for suspected cases | 59 | 14 | 7 | 75 | 6 |
| 493 | Skalidis I 2021[3] | CT for suspected cases | 21 | 8 | 7 | 27 | 6 |
| 493 | Skalidis I 2021[4] | CT for suspected cases | 27 | 2 | 10 | 24 | 6 |
| 494 | Song S 2020 | CT for suspected cases | 99 | 64 | 3 | 45 | 5 |
| 495 | Teichgräber U 2021 | CT for suspected cases | 5 | 14 | 0 | 146 | 6 |
| 426 | Tekcan Sanli DE 2021 | CT for suspected cases | 173 | 58 | 159 | 509 | 3 |
| 496 | Thomas C 2021 | CT for suspected cases | 69 | 60 | 10 | 348 | 6 |
| 497 | Van Berkel B 2021[1] | CT for suspected cases | 60 | 20 | 112 | 8 | 6 |
| 497 | Van Berkel B 2021[2] | CT for suspected cases | 57 | 43 | 89 | 11 | 6 |
| 497 | Van Berkel B 2021[3] | CT for suspected cases | 52 | 24 | 108 | 16 | 6 |
| 497 | Van Berkel B 2021[4] | CT for suspected cases | 60 | 25 | 107 | 8 | 6 |
| 497 | Van Berkel B 2021[5] | CT for suspected cases | 70 | 10 | 111 | 9 | 6 |
| 497 | Van Berkel B 2021[6] | CT for suspected cases | 68 | 32 | 89 | 11 | 6 |
| 497 | Van Berkel B 2021[7] | CT for suspected cases | 62 | 14 | 107 | 17 | 6 |
| 497 | Van Berkel B 2021[8] | CT for suspected cases | 70 | 14 | 107 | 9 | 6 |
| 498 | Vishal Mehta 2022 | CT for suspected cases | 77 | 14 | 18 | 13 | 5 |
| 499 | Wang Y 2020 | CT for suspected cases | 580 | 308 | 21 | 105 | 3 |
| 500 | Wen Z 2020[1] | CT for suspected cases | 82 | 7 | 6 | 8 | 5 |
| 500 | Wen Z 2020[2] | CT for suspected cases | 44 | 3 | 4 | 5 | 5 |
| 500 | Wen Z 2020[3] | CT for suspected cases | 38 | 4 | 2 | 3 | 5 |
| 501 | Xiong Z 2020 | CT for suspected cases | 19 | 19 | 1 | 8 | 6 |
| 502 | Yang H 2020 | CT for suspected cases | 12 | 40 | 1 | 2 | 6 |

CT, Computed tomography; TP, true positive; FP, false positive; FN, false negative; TN, true negative.

**References**

1. Percivale I, Danna PSC, Falaschi Z, et al. Men and women affected by Sars-CoV-2 pneumonia: same CT features but different outcome. *Clin Radiol* 2021;76(3):235 e25-35 e34. doi: 10.1016/j.crad.2020.11.119 [published Online First: 2020/12/29]

2. Knol WG, Thuijs D, Odink AE, et al. Preoperative Chest Computed Tomography Screening for Coronavirus Disease 2019 in Asymptomatic Patients Undergoing Cardiac Surgery. *Semin Thorac Cardiovasc Surg* 2021;33(2):417-24. doi: 10.1053/j.semtcvs.2020.09.027 [published Online First: 2020/09/27]

3. Erxleben C, Adams LC, Albrecht J, et al. Improving CT accuracy in the diagnosis of COVID-19 in a hospital setting. *Clin Imaging* 2021;76:1-5. doi: 10.1016/j.clinimag.2021.01.026 [published Online First: 2021/02/06]

4. Debray MP, Tarabay H, Males L, et al. Observer agreement and clinical significance of chest CT reporting in patients suspected of COVID-19. *Eur Radiol* 2021;31(2):1081-89. doi: 10.1007/s00330-020-07126-8 [published Online First: 2020/08/31]

5. Avcı H, Karabulut B. The Relation Between Otolaryngology-Specific Symptoms and Computed Tomography Findings in Ambulatory Care COVID-19 Patients. *Ear, nose, & throat journal* 2021;100(2):79-85. doi: 10.1177/0145561320975508 [published Online First: 2020/12/05]

6. Tung-Chen Y, Martí de Gracia M, Díez-Tascón A, et al. Correlation between Chest Computed Tomography and Lung Ultrasonography in Patients with Coronavirus Disease 2019 (COVID-19). *Ultrasound Med Biol* 2020;46(11):2918-26. doi: 10.1016/j.ultrasmedbio.2020.07.003 [published Online First: 2020/08/11]

7. Rueckel J, Fink N, Kaestle S, et al. COVID-19 Pandemic and Upcoming Influenza Season-Does an Expert's Computed Tomography Assessment Differentially Identify COVID-19, Influenza and Pneumonias of Other Origin? *J Clin Med* 2020;10(1) doi: 10.3390/jcm10010084 [published Online First: 2021/01/01]

8. Chen HJ, Qiu J, Wu B, et al. Early chest CT features of patients with 2019 novel coronavirus (COVID-19) pneumonia: relationship to diagnosis and prognosis. *Eur Radiol* 2020;30(11):6178-85. doi: 10.1007/s00330-020-06978-4 [published Online First: 2020/06/11]

9. Abdolahi N, Kaheh E, Golsha R, et al. Letter to the editor: efficacy of different methods of combination regimen administrations including dexamethasone, intravenous immunoglobulin, and interferon-beta to treat critically ill COVID-19 patients: a structured summary of a study protocol for a randomized controlled trial. *Trials* 2020;21(1):549. doi: 10.1186/s13063-020-04499-5 [published Online First: 2020/06/21]

10. Alavi Darazam I, Hatami F, Rabiei MM, et al. An investigation into the beneficial effects of high-dose interferon beta 1-a, compared to low-dose interferon beta 1-a (the base therapeutic regimen) in moderate to severe COVID-19: A structured summary of a study protocol for a randomized controlled l trial. *Trials* 2020;21(1):880. doi: 10.1186/s13063-020-04812-2 [published Online First: 2020/10/28]

11. Chaccour C, Ruiz-Castillo P, Richardson MA, et al. The SARS-CoV-2 Ivermectin Navarra-ISGlobal Trial (SAINT) to Evaluate the Potential of Ivermectin to Reduce COVID-19 Transmission in low risk, non-severe COVID-19 patients in the first 48 hours after symptoms onset: A structured summary of a study protocol for a randomized control pilot trial. *Trials* 2020;21(1):498. doi: 10.1186/s13063-020-04421-z [published Online First: 2020/06/10]

12. Chowdhury FR, Hoque A, Chowdhury FUH, et al. Convalescent plasma transfusion therapy in severe COVID-19 patients- a safety, efficacy and dose response study: A structured summary of a study protocol of a phase II randomized controlled trial. *Trials* 2020;21(1):883. doi: 10.1186/s13063-020-04734-z [published Online First: 2020/10/28]

13. Cuadrado-Lavín A, Olmos JM, Cifrian JM, et al. Controlled, double-blind, randomized trial to assess the efficacy and safety of hydroxychloroquine chemoprophylaxis in SARS CoV2 infection in healthcare personnel in the hospital setting: A structured summary of a study protocol for a randomised controlled trial. *Trials* 2020;21(1):472. doi: 10.1186/s13063-020-04400-4 [published Online First: 2020/06/05]

14. Dalili N, Kashefizadeh A, Nafar M, et al. Adding Colchicine to the Antiretroviral Medication - Lopinavir/Ritonavir (Kaletra) in Hospitalized Patients with Non-Severe Covid-19 Pneumonia: A Structured Summary of a Study Protocol for a Randomized Controlled Trial. *Trials* 2020;21(1):489. doi: 10.1186/s13063-020-04455-3 [published Online First: 2020/06/07]

15. Emadi A, Chua JV, Talwani R, et al. Safety and Efficacy of Imatinib for Hospitalized Adults with COVID-19: A structured summary of a study protocol for a randomised controlled trial. *Trials* 2020;21(1):897. doi: 10.1186/s13063-020-04819-9 [published Online First: 2020/10/30]

16. Hassaniazad M, Bazram A, Hassanipour S, et al. Evaluation of the efficacy and safety of favipiravir and interferon compared to lopinavir/ritonavir and interferon in moderately ill patients with COVID-19: a structured summary of a study protocol for a randomized controlled trial. *Trials* 2020;21(1):886. doi: 10.1186/s13063-020-04747-8 [published Online First: 2020/10/29]

17. Natarajan S, Anbarasi C, Sathiyarajeswaran P, et al. The efficacy of Siddha Medicine, Kabasura Kudineer (KSK) compared to Vitamin C & Zinc (CZ) supplementation in the management of asymptomatic COVID-19 cases: A structured summary of a study protocol for a randomised controlled trial. *Trials* 2020;21(1):892. doi: 10.1186/s13063-020-04823-z [published Online First: 2020/10/29]

18. Salarifar M, Ghavami M, Poorhosseini H, et al. The impact of a dedicated coronavirus disease 2019 primary angioplasty protocol on time components related to ST‑segment elevation myocardial infarction management in a 24/7 primary percutaneous coronary intervention-capable hospital. *Kardiologia polska* 2020;78(12):1227-34. doi: 10.33963/kp.15607 [published Online First: 2020/09/22]

19. Dacrema A, Silva M, Rovero L, et al. A simple lung ultrasound protocol for the screening of COVID-19 pneumonia in the emergency department. *Internal and emergency medicine* 2021;16(5):1297-305. doi: 10.1007/s11739-020-02596-6 [published Online First: 2021/01/12]

20. Garcia PJ, Mundaca H, Ugarte-Gil C, et al. Randomized clinical trial to compare the efficacy of ivermectin versus placebo to negativize nasopharyngeal PCR in patients with early COVID-19 in Peru (SAINT-Peru): a structured summary of a study protocol for randomized controlled trial. *Trials* 2021;22(1):262. doi: 10.1186/s13063-021-05236-2 [published Online First: 2021/04/11]

21. Hosseini FS, Malektojari A, Ghazizadeh S, et al. The efficacy and safety of Ivermectin in patients with mild and moderate COVID-19: A structured summary of a study protocol for a randomized controlled trial. *Trials* 2021;22(1):4. doi: 10.1186/s13063-020-04988-7 [published Online First: 2021/01/06]

22. Miyake S, Higurashi T, Kato H, et al. Evaluation of a combination protocol of CT-first triage and active telemedicine methods by a selected team tackling COVID-19: An experimental research study. *J Infect Public Health* 2021;14(9):1212-17. doi: 10.1016/j.jiph.2021.08.016 [published Online First: 2021/08/24]

23. Muller M, Lefebvre F, Harlay ML, et al. Impact of intravenous lidocaine on clinical outcomes of patients with ARDS during COVID-19 pandemia (LidoCovid): A structured summary of a study protocol for a randomised controlled trial. *Trials* 2021;22(1):131. doi: 10.1186/s13063-021-05095-x [published Online First: 2021/02/13]

24. Payares-Herrera C, Martínez-Muñoz ME, Vallhonrat IL, et al. Double-blind, randomized, controlled, trial to assess the efficacy of allogenic mesenchymal stromal cells in patients with acute respiratory distress syndrome due to COVID-19 (COVID-AT): A structured summary of a study protocol for a randomised controlled trial. *Trials* 2021;22(1):9. doi: 10.1186/s13063-020-04964-1 [published Online First: 2021/01/08]

25. Pourdowlat G, Mousavinasab SR, Farzanegan B, et al. Evaluation of the efficacy and safety of inhaled magnesium sulphate in combination with standard treatment in patients with moderate or severe COVID-19: A structured summary of a study protocol for a randomised controlled trial. *Trials* 2021;22(1):60. doi: 10.1186/s13063-021-05032-y [published Online First: 2021/01/20]

26. Rahimi H, Allahyari A, Ataei Azimi S, et al. Effect of hydroxychloroquine on COVID-19 prevention in cancer patients undergoing treatment: study protocol for a randomized controlled trial. *Trials* 2021;22(1):349. doi: 10.1186/s13063-021-05292-8 [published Online First: 2021/05/21]

27. Rodríguez Flores SN, Rodríguez-Martínez LM, Reyes-Berrones BL, et al. Comparison Between a Standard and SalivaDirect RNA Extraction Protocol for Molecular Diagnosis of SARS-CoV-2 Using Nasopharyngeal Swab and Saliva Clinical Samples. *Frontiers in bioengineering and biotechnology* 2021;9:638902. doi: 10.3389/fbioe.2021.638902 [published Online First: 2021/04/16]

28. Srivastava A, Rengaraju M, Srivastava S, et al. A double blinded placebo controlled comparative clinical trial to evaluate the effectiveness of Siddha medicines, Kaba Sura Kudineer (KSK) & Nilavembu Kudineer (NVK) along with standard Allopathy treatment in the management of symptomatic COVID 19 patients - a structured summary of a study protocol for a randomized controlled trial. *Trials* 2021;22(1):130. doi: 10.1186/s13063-021-05041-x [published Online First: 2021/02/13]

29. Stessel B, Callebaut I, Polus F, et al. Evaluation of a comprehensive pre-procedural screening protocol for COVID-19 in times of a high SARS CoV-2 prevalence: a prospective cross-sectional study. *Ann Med* 2021;53(1):337-44. doi: 10.1080/07853890.2021.1878272 [published Online First: 2021/02/16]

30. Yadav B, Rai A, Mundada PS, et al. Safety and efficacy of Ayurvedic interventions and Yoga on long term effects of COVID-19: A structured summary of a study protocol for a randomized controlled trial. *Trials* 2021;22(1):378. doi: 10.1186/s13063-021-05326-1 [published Online First: 2021/06/05]

31. Ashraf S, Ashraf S, Ashraf M, et al. Clinical efficacy of iodine complex in SARS-CoV-2-infected patients with mild to moderate symptoms: study protocol for a randomized controlled trial. *Trials* 2022;23(1):58. doi: 10.1186/s13063-021-05848-8 [published Online First: 2022/01/21]

32. Barros FRO, Leite DCA, Guimarães LJ, et al. Performance of RT-qPCR detection of SARS-CoV-2 in unextracted nasopharyngeal samples using the Seegene Allplex(TM) 2019-nCoV protocol. *J Virol Methods* 2022;300:114429. doi: 10.1016/j.jviromet.2021.114429 [published Online First: 2021/12/18]

33. de Oliveira LPR, Cabral AD, Dos Santos Carmo AM, et al. Alternative SARS-CoV-2 detection protocol from self-collected saliva for mass diagnosis and epidemiological studies in low-incoming regions. *J Virol Methods* 2022;300:114382. doi: 10.1016/j.jviromet.2021.114382 [published Online First: 2021/11/30]

34. Gdoura M, Abouda I, Mrad M, et al. SARS-CoV2 RT-PCR assays: In vitro﻿ comparison of 4 WHO approved protocols on clinical specimens and its implications for real laboratory practice through variant emergence. *Virol J* 2022;19(1):54. doi: 10.1186/s12985-022-01784-4 [published Online First: 2022/03/30]

35. Hiemstra AM, MacDonald CE, van Rensburg IC, et al. Cascade Immune Mechanisms of Protection against Mycobacterium tuberculosis (IMPAc-TB): study protocol for the Household Contact Study in the Western Cape, South Africa. *BMC Infect Dis* 2022;22(1):381. doi: 10.1186/s12879-022-07349-8 [published Online First: 2022/04/17]

36. Khorasanchi Z, Jafazadeh Esfehani A, Sharifan P, et al. The effects of high dose vitamin D supplementation as a nutritional intervention strategy on biochemical and inflammatory factors in adults with COVID-19: Study protocol for a randomized controlled trial. *Nutr Health* 2022;28(3):311-17. doi: 10.1177/02601060221082384 [published Online First: 2022/03/25]

37. Martinez M, Nguyen PV, Su M, et al. SARS-CoV-2 Variants in Paraguay: Detection and Surveillance with an Economical and Scalable Molecular Protocol. *Viruses* 2022;14(5) doi: 10.3390/v14050873 [published Online First: 2022/05/29]

38. Park JY, Lee JH, Cha BK, et al. Analysis of the Efficacy of Universal Screening of Coronavirus Disease with Antigen-Detecting Rapid Diagnostic Tests at Point-or-Care Settings and Sharing the Experience of Admission Protocol-A Pilot Study. *J Pers Med* 2022;12(2) doi: 10.3390/jpm12020319 [published Online First: 2022/02/26]

39. Zuberi S, Mushtaq Y, Patel K, et al. COVID-19 Diagnosis in Patients With Acute Abdominal Pain Without Respiratory Symptoms: A UK Emergency General Surgical Unit Experience. *Am Surg* 2022:31348221114033. doi: 10.1177/00031348221114033 [published Online First: 2022/07/13]

40. Zamani N, Gheshlaghi F, Haghighi-Morad M, et al. Prevalence of clinical and radiologic features in methanol-poisoned patients with and without COVID-19 infection. *Acute medicine & surgery* 2021;8(1):e715. doi: 10.1002/ams2.715 [published Online First: 2021/12/21]

41. Westblade LF, Brar G, Pinheiro LC, et al. SARS-CoV-2 Viral Load Predicts Mortality in Patients with and without Cancer Who Are Hospitalized with COVID-19. *Cancer Cell* 2020;38(5):661-71.e2. doi: 10.1016/j.ccell.2020.09.007 [published Online First: 2020/10/01]

42. Tirumani SH, Rahnemai-Azar AA, Pierce JD, et al. Are asymptomatic gastrointestinal findings on imaging more common in COVID-19 infection? Study to determine frequency of abdominal findings of COVID-19 infection in patients with and without abdominal symptoms and in patients with chest-only CT scans. *Abdominal radiology (New York)* 2021;46(6):2407-14. doi: 10.1007/s00261-020-02920-w [published Online First: 2021/01/05]

43. Tekcan Sanli DE, Altundag A, Yıldırım D, et al. Comparison of Olfactory Cleft Width and Volumes in Patients with COVID-19 Anosmia and COVID-19 Cases Without Anosmia. *ORL; journal for oto-rhino-laryngology and its related specialties* 2022;84(1):1-9. doi: 10.1159/000518672 [published Online First: 2021/09/28]

44. Shi LY, Cai YP, Zhang YX, et al. [A comparison of clinical characteristics between patients with and without"recurrence"RNA positive with COVID-19]. *Zhonghua Jie He He Hu Xi Za Zhi* 2020;43(12):1066-70. doi: 10.3760/cma.j.cn112147-20200602-00668 [published Online First: 2020/12/18]

45. Sayit AT, Elmali M, Deveci A, et al. Relationship between acute phase reactants and prognosis in patients with or without COVID-19 pneumonia. *Revista do Instituto de Medicina Tropical de Sao Paulo* 2021;63:e51. doi: 10.1590/s1678-9946202163051 [published Online First: 2021/07/01]

46. Possari RY, Andrade-Gomes HJ, Mello VC, et al. Association of coronary calcification with prognosis of Covid-19 patients without known heart disease. *Braz J Med Biol Res* 2021;54(12):e11681. doi: 10.1590/1414-431X2021e11681 [published Online First: 2021/12/09]

47. Ozturk D, Gareayaghi N, Tahtasakal CA, et al. Antibody responses after two doses of CoronaVac of the participants with or without the diagnosis of COVID-19. *Ir J Med Sci* 2022:1-6. doi: 10.1007/s11845-021-02883-x [published Online First: 2022/01/11]

48. O'Hara RW, Brown B, Hughes A, et al. Evaluation of the artus® Prep&Amp UM RT-PCR for detection of SARS-CoV-2 from nasopharyngeal swabs without prior nucleic acid eluate extraction. *Journal of clinical virology plus* 2022;2(3):100098. doi: 10.1016/j.jcvp.2022.100098 [published Online First: 2022/07/26]

49. Mudenda V, Mumba C, Pieciak RC, et al. Histopathological Evaluation of Deceased Persons in Lusaka, Zambia With or Without Coronavirus Disease 2019 (COVID-19) Infection: Results Obtained From Minimally Invasive Tissue Sampling. *Clin Infect Dis* 2021;73(Suppl_5):S465-s71. doi: 10.1093/cid/ciab858 [published Online First: 2021/12/16]

50. Morais O, Alves MR, Ramos C, et al. The Matrix Effect in the RT-PCR Detection of SARS-CoV-2 Using Saliva without RNA Extraction. *Diagnostics (Basel, Switzerland)* 2022;12(7) doi: 10.3390/diagnostics12071547 [published Online First: 2022/07/28]

51. Li Z, Zeng B, Lei P, et al. Differentiating pneumonia with and without COVID-19 using chest CT images: from qualitative to quantitative. *J Xray Sci Technol* 2020;28(4):583-89. doi: 10.3233/xst-200689 [published Online First: 2020/06/23]

52. Li X, Chan JF, Li KK, et al. Detection of SARS-CoV-2 in conjunctival secretions from patients without ocular symptoms. *Infection* 2021;49(2):257-65. doi: 10.1007/s15010-020-01524-2 [published Online First: 2020/09/19]

53. Hedayat B, Hosseini K. Chest pain and high troponin level without significant respiratory symptoms in young patients with COVID-19. *Caspian J Intern Med* 2020;11(Suppl 1):561-65. doi: 10.22088/cjim.11.0.561 [published Online First: 2021/01/12]

54. Hasan MR, Mirza F, Al-Hail H, et al. Detection of SARS-CoV-2 RNA by direct RT-qPCR on nasopharyngeal specimens without extraction of viral RNA. *PLoS One* 2020;15(7):e0236564. doi: 10.1371/journal.pone.0236564 [published Online First: 2020/07/25]

55. Eser-Ozturk H, Izci Duran T, Aydog O, et al. Sarcoid-like Uveitis with or without Tubulointerstitial Nephritis during COVID-19. *Ocular immunology and inflammation* 2022:1-8. doi: 10.1080/09273948.2022.2032195 [published Online First: 2022/02/25]

56. Doglietto F, Vezzoli M, Gheza F, et al. Factors Associated With Surgical Mortality and Complications Among Patients With and Without Coronavirus Disease 2019 (COVID-19) in Italy. *JAMA Surg* 2020;155(8):691-702. doi: 10.1001/jamasurg.2020.2713 [published Online First: 2020/06/13]

57. Daval M, Corré A, Palpacuer C, et al. Efficacy of local budesonide therapy in the management of persistent hyposmia in COVID-19 patients without signs of severity: A structured summary of a study protocol for a randomised controlled trial. *Trials* 2020;21(1):666. doi: 10.1186/s13063-020-04585-8 [published Online First: 2020/07/22]

58. Dai Q, Ye M, Tang Z, et al. Comparison of severe and critical COVID-19 patients imported from Russia with and without influenza A infection in Heilongjiang Province: a retrospective study. *Annals of translational medicine* 2021;9(18):1446. doi: 10.21037/atm-21-3912 [published Online First: 2021/11/05]

59. Beltrán-Pavez C, Alonso-Palomares LA, Valiente-Echeverría F, et al. Accuracy of a RT-qPCR SARS-CoV-2 detection assay without prior RNA extraction. *J Virol Methods* 2021;287:113969. doi: 10.1016/j.jviromet.2020.113969 [published Online First: 2020/09/13]

60. Barza R, Patel P, Sabatini L, et al. Use of a simplified sample processing step without RNA extraction for direct SARS-CoV-2 RT-PCR detection. *Journal of clinical virology : the official publication of the Pan American Society for Clinical Virology* 2020;132:104587. doi: 10.1016/j.jcv.2020.104587 [published Online First: 2020/09/09]

61. Assaad S, Zrounba P, Cropet C, et al. Mortality of patients with solid and haematological cancers presenting with symptoms of COVID-19 with vs without detectable SARS-COV-2: a French nationwide prospective cohort study. *Br J Cancer* 2021;125(5):658-71. doi: 10.1038/s41416-021-01452-4 [published Online First: 2021/06/18]

62. Asahara Y, Mukai T, Suda M, et al. [The odor stick identification test for Japanese (OSIT-J) in a case of coronavirus disease 2019 (COVID-19) without pneumonia]. *Rinsho shinkeigaku = Clinical neurology* 2021;61(2):140-43. doi: 10.5692/clinicalneurol.cn-001517 [published Online First: 2021/01/29]

63. Albano D, Camoni L, Rinaldi R, et al. 18F-FDG PET/CT Metabolic Behavior of COVID-19 Pneumonia: A Series of 4 Patients With RT-PCR Confirmation. *Clin Nucl Med* 2020;45(8):e378-e80. doi: 10.1097/rlu.0000000000003150 [published Online First: 2020/06/11]

64. Boulvard Chollet XLE, Romero Robles LG, Garrastachu P, et al. 18F-FDG PET/CT in Hodgkin Lymphoma With Unsuspected COVID-19. *Clin Nucl Med* 2020;45(8):652-53. doi: 10.1097/rlu.0000000000003143 [published Online First: 2020/06/11]

65. Cosma L, Sollaku S, Frantellizzi V, et al. Early (18) F-FDG PET/CT in COVID-19. *J Med Imaging Radiat Oncol* 2020;64(5):671-73. doi: 10.1111/1754-9485.13099 [published Online First: 2020/09/19]

66. Grimaldi S, Lagarde S, Harlé JR, et al. Autoimmune Encephalitis Concomitant with SARS-CoV-2 Infection: Insight from (18)F-FDG PET Imaging and Neuronal Autoantibodies. *Journal of nuclear medicine : official publication, Society of Nuclear Medicine* 2020;61(12):1726-29. doi: 10.2967/jnumed.120.249292 [published Online First: 2020/07/28]

67. Habouzit V, Sanchez A, Dehbi S, et al. Incidental Finding of COVID-19 Lung Infection in 18F-FDG PET/CT: What Should We Do? *Clin Nucl Med* 2020;45(8):649-51. doi: 10.1097/rlu.0000000000003135 [published Online First: 2020/06/20]

68. Johnson LN, Vesselle H. COVID-19 in an asymptomatic patient undergoing FDG PET/CT. *Radiol Case Rep* 2020;15(10):1809-12. doi: 10.1016/j.radcr.2020.07.018 [published Online First: 2020/08/14]

69. Khattab MH, Sherry AD, Jessop AC, et al. Early detection of SARS-CoV-2 from staging PET-CT. *J Radiat Oncol* 2020;9(3-4):93-95. doi: 10.1007/s13566-020-00436-w [published Online First: 2020/10/06]

70. Krebs S, Petkovska I, Ho AL, et al. Laboratory-Proven Asymptomatic SARS-CoV-2 (COVID-19) Infection on 18F-FDG PET/CT. *Clin Nucl Med* 2020;45(8):654-55. doi: 10.1097/rlu.0000000000003141 [published Online First: 2020/05/21]

71. Lütje S, Marinova M, Kütting D, et al. Nuclear medicine in SARS-CoV-2 pandemia: 18F-FDG-PET/CT to visualize COVID-19. *Nuklearmedizin* 2020;59(3):276-80. doi: 10.1055/a-1152-2341 [published Online First: 2020/04/08]

72. Martí A, Morón S, González E, et al. Incidental findings of COVID-19 in F18-FDG PET/CT from asymptomatic patients with cancer in two healthcare institutions in Bogotá, Colombia. *Biomedica : revista del Instituto Nacional de Salud* 2020;40(Supl. 2):27-33. doi: 10.7705/biomedica.5833 [published Online First: 2020/11/06]

73. Milardovic R, Beslic N, Ceric S, et al. Positron Emission Tomography Scan (PET/CT) During the COVID-19 Pandemic: a Case Series. *Acta informatica medica : AIM : journal of the Society for Medical Informatics of Bosnia & Herzegovina : casopis Drustva za medicinsku informatiku BiH* 2020;28(4):292-97. doi: 10.5455/aim.2020.28.292-297 [published Online First: 2021/02/26]

74. Mo A, Brodin NP, Tomé WA, et al. COVID-19 Incidentally Detected on PET/CT During Work-up for Locally Advanced Head and Neck Cancer. *In Vivo* 2020;34(3 Suppl):1681-84. doi: 10.21873/invivo.11961 [published Online First: 2020/06/07]

75. Morón S, González E, Rojas J. 68Ga-PSMA PET/CT With Incidental Finding of COVID-19 in an Asymptomatic Patient. *Clin Nucl Med* 2020;45(12):1032-33. doi: 10.1097/rlu.0000000000003388 [published Online First: 2020/10/17]

76. Olivari L, Riccardi N, Rodari P, et al. Accidental diagnosis of COVID-19 pneumonia after 18F FDG PET/CT: a case series. *Clin Transl Imaging* 2020;8(5):393-400. doi: 10.1007/s40336-020-00388-8 [published Online First: 2020/09/30]

77. Playe M, Siavellis J, Braun T, et al. FDG PET/CT in a Patient With Mantle Cell Lymphoma and COVID-19: Typical Findings. *Clin Nucl Med* 2020;45(7):e305-e06. doi: 10.1097/rlu.0000000000003113 [published Online First: 2020/05/27]

78. Sinha P, Sinha S, Schlehr E, et al. COVID-19: Incidental Diagnosis by 18F-FDG PET/CT. *Clin Nucl Med* 2020;45(8):659-60. doi: 10.1097/rlu.0000000000003154 [published Online First: 2020/06/11]

79. Zanoni L, Mosconi C, Cervati V, et al. [18F]-FDG PET/CT for suspected lymphoma relapse in a patient with concomitant pneumococcal pneumonia during COVID-19 outbreak: unexpected SARS-Cov-2 co-infection despite double RT-PCR negativity. *Eur J Nucl Med Mol Imaging* 2020;47(8):2038-39. doi: 10.1007/s00259-020-04838-3 [published Online First: 2020/05/21]

80. Bai Y, Xu J, Chen L, et al. Inflammatory response in lungs and extrapulmonary sites detected by [(18)F] fluorodeoxyglucose PET/CT in convalescing COVID-19 patients tested negative for coronavirus. *Eur J Nucl Med Mol Imaging* 2021;48(8):2531-42. doi: 10.1007/s00259-020-05083-4 [published Online First: 2021/01/10]

81. Bello Martinez R, Ghesani M, Ghesani N, et al. Asymptomatic SARS-CoV-2 infection: Incidental findings on FDG PET/CT. *J Med Imaging Radiat Sci* 2021;52(2):179-85. doi: 10.1016/j.jmir.2021.03.002 [published Online First: 2021/04/07]

82. Brogna B, Bignardi E, Brogna C, et al. Typical CT findings of COVID-19 pneumonia in patients presenting with repetitive negative RT-PCR. *Radiography (London, England : 1995)* 2021;27(2):743-47. doi: 10.1016/j.radi.2020.09.012 [published Online First: 2020/10/02]

83. Cabrera Villegas A, Romero Robles LG, Boulvard Chollet XLE, et al. [(18)F]-FDG PET/CT in oncologic patients with unsuspected asymptomatic infection with SARS-CoV-2. *Eur J Nucl Med Mol Imaging* 2021;48(3):786-93. doi: 10.1007/s00259-020-04979-5 [published Online First: 2020/09/17]

84. Chammas A, Namer IJ, Lersy F, et al. Inferior Colliculus's Hypermetabolism: A New Finding on Brain FDG PET and Perfusion MRI in a Patient With COVID-19. *Clin Nucl Med* 2021;46(5):413-14. doi: 10.1097/rlu.0000000000003592 [published Online First: 2021/03/07]

85. Kas A, Soret M, Pyatigoskaya N, et al. The cerebral network of COVID-19-related encephalopathy: a longitudinal voxel-based 18F-FDG-PET study. *Eur J Nucl Med Mol Imaging* 2021;48(8):2543-57. doi: 10.1007/s00259-020-05178-y [published Online First: 2021/01/17]

86. Kawataki M, Ito A, Ishida T. Pneumonia Due to Human Coronavirus OC43 in an Immunocompetent Adult Detected by Multiplex Polymerase Chain Reaction. *Intern Med* 2021;60(21):3497-501. doi: 10.2169/internalmedicine.7450-21 [published Online First: 2021/08/27]

87. Morón S, González E, Rojas J. 68Ga-DOTANOC PET/CT With Lung Involvement in the Era of COVID-19 Pandemic. *Clin Nucl Med* 2021;46(2):166-67. doi: 10.1097/rlu.0000000000003473 [published Online First: 2020/11/26]

88. Piciu A, Manole S, Piciu D, et al. Asymptomatic COVID-19 cancer patients incidentally discovered during F18-FDG PET/CT monitoring. *Med Pharm Rep* 2021;94(1):58-64. doi: 10.15386/mpr-1776 [published Online First: 2021/02/26]

89. Pillenahalli Maheshwarappa R, Graham MM. Asymptomatic COVID-19 Infection Detected on 18F-FDG PET/CT Scan Done for Multiple Myeloma. *Clin Nucl Med* 2021;46(1):e57-e58. doi: 10.1097/rlu.0000000000003314 [published Online First: 2020/09/22]

90. Sajad T, Hassan A. Incidental COVID-19 pneumonia on oncologic 18F-FDG PET-CT scan in asymptomatic patient. *JPMA The Journal of the Pakistan Medical Association* 2021;71(12):2834. [published Online First: 2022/02/13]

91. Tekin D, Budak E, Yoldas B, et al. Why is it important to report early possible COVID-19 PET/CT findings in cancer patients? Explaining with a case series. *Indian J Cancer* 2021;58(2):248-58. doi: 10.4103/ijc.IJC_645_20 [published Online First: 2021/06/09]

92. Yeh R, Elsakka A, Wray R, et al. FDG PET/CT imaging features and clinical utility in COVID-19. *Clinical imaging* 2021;80:262-67. doi: 10.1016/j.clinimag.2021.08.002 [published Online First: 2021/08/22]

93. Aksu A, Bozkurt K, Yilmaz B. Tracheitis Diagnosed With 68Ga-PSMA PET/CT in a Patient With COVID-19. *Clin Nucl Med* 2022 doi: 10.1097/rlu.0000000000004288 [published Online First: 2022/05/28]

94. Kerr C, O'Neill S, Szucs A, et al. Zoster meningitis in an immunocompetent young patient post first dose of BNT162b2 mRNA COVID-19 vaccine, a case report. *IDCases* 2022;27:e01452. doi: 10.1016/j.idcr.2022.e01452 [published Online First: 2022/02/22]

95. Matsui J, Kandathil A, Peng F. FDG PET/CT findings and post-treatment changes of COVID-19 pneumonia in a patient with lymphoma: A case report. *Mol Clin Oncol* 2022;16(3):56. doi: 10.3892/mco.2021.2489 [published Online First: 2022/02/04]

96. Okudan B, Seven B, Ural B, et al. Detection of COVID-19 Incidentally in 68Ga-PSMA PET/CT for Restaging of Prostate Cancer. *Current medical imaging* 2022 doi: 10.2174/1573405618666220427134647 [published Online First: 2022/05/02]

97. Subesinghe M, Bhuva S, Dunn JT, et al. A case-control evaluation of pulmonary and extrapulmonary findings of incidental asymptomatic COVID-19 infection on FDG PET-CT. *The British journal of radiology* 2022;95(1130):20211079. doi: 10.1259/bjr.20211079 [published Online First: 2021/12/22]

98. Abbasi-Oshaghi E, Mirzaei F, Farahani F, et al. Diagnosis and treatment of coronavirus disease 2019 (COVID-19): Laboratory, PCR, and chest CT imaging findings. *International journal of surgery (London, England)* 2020;79:143-53. doi: 10.1016/j.ijsu.2020.05.018 [published Online First: 2020/05/19]

99. Addetia A, Crawford KHD, Dingens A, et al. Neutralizing Antibodies Correlate with Protection from SARS-CoV-2 in Humans during a Fishery Vessel Outbreak with a High Attack Rate. *J Clin Microbiol* 2020;58(11) doi: 10.1128/jcm.02107-20 [published Online First: 2020/08/23]

100. Anantharaj A, Das SJ, Sharanabasava P, et al. Visual Detection of SARS-CoV-2 RNA by Conventional PCR-Induced Generation of DNAzyme Sensor. *Frontiers in molecular biosciences* 2020;7:586254. doi: 10.3389/fmolb.2020.586254 [published Online First: 2021/01/12]

101. Assi T, Samra B, Dercle L, et al. Screening Strategies for COVID-19 in Patients With Hematologic Malignancies. *Front Oncol* 2020;10:1267. doi: 10.3389/fonc.2020.01267 [published Online First: 2020/07/29]

102. Babiker A, Bradley HL, Stittleburg VD, et al. Metagenomic Sequencing To Detect Respiratory Viruses in Persons under Investigation for COVID-19. *J Clin Microbiol* 2020;59(1) doi: 10.1128/jcm.02142-20 [published Online First: 2020/10/18]

103. Beigmohammadi MT, Bitarafan S, Hoseindokht A, et al. Impact of vitamins A, B, C, D, and E supplementation on improvement and mortality rate in ICU patients with coronavirus-19: a structured summary of a study protocol for a randomized controlled trial. *Trials* 2020;21(1):614. doi: 10.1186/s13063-020-04547-0 [published Online First: 2020/07/08]

104. Bitker L, Dhelft F, Chauvelot L, et al. Protracted viral shedding and viral load are associated with ICU mortality in Covid-19 patients with acute respiratory failure. *Ann Intensive Care* 2020;10(1):167. doi: 10.1186/s13613-020-00783-4 [published Online First: 2020/12/11]

105. Blagojevic NR, Bosnjakovic D, Vukomanovic V, et al. Acute pericarditis and severe acute respiratory syndrome coronavirus 2: Case report. *International journal of infectious diseases : IJID : official publication of the International Society for Infectious Diseases* 2020;101:180-82. doi: 10.1016/j.ijid.2020.09.1440 [published Online First: 2020/10/02]

106. Bordi L, Sberna G, Lalle E, et al. Frequency and Duration of SARS-CoV-2 Shedding in Oral Fluid Samples Assessed by a Modified Commercial Rapid Molecular Assay. *Viruses* 2020;12(10) doi: 10.3390/v12101184 [published Online First: 2020/10/24]

107. Brito CAA, Brito MCM, Martins THF, et al. Clinical laboratory and dispersion pattern of COVID-19 in a family cluster in the social-distancing period. *Journal of infection in developing countries* 2020;14(9):987-93. doi: 10.3855/jidc.13580 [published Online First: 2020/10/09]

108. Bullard J, Dust K, Funk D, et al. Predicting Infectious Severe Acute Respiratory Syndrome Coronavirus 2 From Diagnostic Samples. *Clin Infect Dis* 2020;71(10):2663-66. doi: 10.1093/cid/ciaa638 [published Online First: 2020/05/23]

109. Cai X, Ma Y, Li S, et al. Clinical Characteristics of 5 COVID-19 Cases With Non-respiratory Symptoms as the First Manifestation in Children. *Frontiers in pediatrics* 2020;8:258. doi: 10.3389/fped.2020.00258 [published Online First: 2020/06/24]

110. Castellvi J, Jerico C, DeMiguel A, et al. Impact on clinical practice of the preoperative screening of Covid-19 infection in surgical oncological patients. Prospective cohort study. *Int J Surg Open* 2020;26:30-35. doi: 10.1016/j.ijso.2020.08.003 [published Online First: 2020/01/01]

111. Chaimayo C, Kaewnaphan B, Tanlieng N, et al. Rapid SARS-CoV-2 antigen detection assay in comparison with real-time RT-PCR assay for laboratory diagnosis of COVID-19 in Thailand. *Virol J* 2020;17(1):177. doi: 10.1186/s12985-020-01452-5 [published Online First: 2020/11/15]

112. Chakraborty C, Sharma AR, Sharma G, et al. SARS-CoV-2 causing pneumonia-associated respiratory disorder (COVID-19): diagnostic and proposed therapeutic options. *Eur Rev Med Pharmacol Sci* 2020;24(7):4016-26. doi: 10.26355/eurrev_202004_20871 [published Online First: 2020/04/25]

113. Chao H, Fang X, Zhang J, et al. Integrative Analysis for COVID-19 Patient Outcome Prediction. *ArXiv* 2020 [published Online First: 2020/08/04]

114. Colombi D, Bodini FC, Petrini M, et al. Well-aerated Lung on Admitting Chest CT to Predict Adverse Outcome in COVID-19 Pneumonia. *Radiology* 2020;296(2):E86-e96. doi: 10.1148/radiol.2020201433 [published Online First: 2020/04/18]

115. Conrozier T, Lohse A, Balblanc JC, et al. Biomarker variation in patients successfully treated with tocilizumab for severe coronavirus disease 2019 (COVID-19): results of a multidisciplinary collaboration. *Clin Exp Rheumatol* 2020;38(4):742-47. [published Online First: 2020/06/24]

116. Eslamijouybari M, Heydari K, Maleki I, et al. Neutrophil-to-Lymphocyte and Platelet-to-Lymphocyte Ratios in COVID-19 Patients and Control Group and Relationship with Disease Prognosis. *Caspian J Intern Med* 2020;11(Suppl 1):531-35. doi: 10.22088/cjim.11.0.531 [published Online First: 2021/01/12]

117. Gheysarzadeh A, Sadeghifard N, Safari M, et al. Report of five nurses infected with severe acute respiratory syndrome coronavirus 2 during patient care: case series. *New Microbes New Infect* 2020;36:100694. doi: 10.1016/j.nmni.2020.100694 [published Online First: 2020/05/15]

118. Giannitto C, Sposta FM, Repici A, et al. Chest CT in patients with a moderate or high pretest probability of COVID-19 and negative swab. *Radiol Med* 2020;125(12):1260-70. doi: 10.1007/s11547-020-01269-w [published Online First: 2020/08/31]

119. Gökce M, Yin S, Sönmez MG, et al. How does the COVID-19 pandemic affect the preoperative evaluation and anesthesia applied for urinary stones? EULIS eCORE-IAU multicenter collaborative cohort study. *Urolithiasis* 2020;48(4):345-51. doi: 10.1007/s00240-020-01193-8 [published Online First: 2020/05/22]

120. Guneysu F, Yurumez Y, Guclu E, et al. The diagnostic process of covıd-19 in the emergency department: laboratory and ımaging methods. *Rev Assoc Med Bras (1992)* 2020;66Suppl 2(Suppl 2):58-64. doi: 10.1590/1806-9282.66.S2.58 [published Online First: 2020/09/24]

121. Guo X, Li Y, Li H, et al. An improved multivariate model that distinguishes COVID-19 from seasonal flu and other respiratory diseases. *Aging* 2020;12(20):19938-44. doi: 10.18632/aging.104132 [published Online First: 2020/10/22]

122. Haddar C, Verhoeven PO, Bourlet T, et al. Brief comparative evaluation of six open one-step RT-qPCR mastermixes for the detection of SARS-CoV-2 RNA using a Taqman probe. *Journal of clinical virology : the official publication of the Pan American Society for Clinical Virology* 2020;132:104636. doi: 10.1016/j.jcv.2020.104636 [published Online First: 2020/10/26]

123. Hirotsu Y, Mochizuki H, Omata M. Double-quencher probes improve detection sensitivity toward Severe Acute Respiratory Syndrome Coronavirus 2 (SARS-CoV-2) in a reverse-transcription polymerase chain reaction (RT-PCR) assay. *J Virol Methods* 2020;284:113926. doi: 10.1016/j.jviromet.2020.113926 [published Online First: 2020/07/11]

124. Hossain R, Lazarus MS, Roudenko A, et al. CT Scans Obtained for Nonpulmonary Indications: Associated Respiratory Findings of COVID-19. *Radiology* 2020;296(3):E173-e79. doi: 10.1148/radiol.2020201743 [published Online First: 2020/05/12]

125. Jiang X, Yin Z, Wang T, et al. COVID-19 Dynamic Computed Tomography (CT) Performance and Observation of Some Laboratory Indicators. *Medical science monitor : international medical journal of experimental and clinical research* 2020;26:e924403. doi: 10.12659/msm.924403 [published Online First: 2020/05/06]

126. Kane AD, Paterson J, Pokhrel S, et al. Peri-operative COVID-19 infection in urgent elective surgery during a pandemic surge period: a retrospective observational cohort study. *Anaesthesia* 2020;75(12):1596-604. doi: 10.1111/anae.15281 [published Online First: 2020/10/23]

127. Kannan NB, Sen S, Reddy H, et al. Preoperative COVID-19 testing for elective vitreoretinal surgeries: Experience from a major tertiary care institute in South India. *Indian journal of ophthalmology* 2020;68(11):2373-77. doi: 10.4103/ijo.IJO_2870_20 [published Online First: 2020/10/31]

128. Kim ES, Chin BS, Kang CK, et al. Clinical Course and Outcomes of Patients with Severe Acute Respiratory Syndrome Coronavirus 2 Infection: a Preliminary Report of the First 28 Patients from the Korean Cohort Study on COVID-19. *J Korean Med Sci* 2020;35(13):e142. doi: 10.3346/jkms.2020.35.e142 [published Online First: 2020/04/04]

129. Kociolek LK, Muller WJ, Yee R, et al. Comparison of Upper Respiratory Viral Load Distributions in Asymptomatic and Symptomatic Children Diagnosed with SARS-CoV-2 Infection in Pediatric Hospital Testing Programs. *J Clin Microbiol* 2020;59(1) doi: 10.1128/jcm.02593-20 [published Online First: 2020/10/24]

130. Li D, Wang D, Dong J, et al. False-Negative Results of Real-Time Reverse-Transcriptase Polymerase Chain Reaction for Severe Acute Respiratory Syndrome Coronavirus 2: Role of Deep-Learning-Based CT Diagnosis and Insights from Two Cases. *Korean journal of radiology* 2020;21(4):505-08. doi: 10.3348/kjr.2020.0146 [published Online First: 2020/03/17]

131. Li W, Cui H, Li K, et al. Chest computed tomography in children with COVID-19 respiratory infection. *Pediatric radiology* 2020;50(6):796-99. doi: 10.1007/s00247-020-04656-7 [published Online First: 2020/03/13]

132. Lippi G, Simundic AM, Plebani M. Potential preanalytical and analytical vulnerabilities in the laboratory diagnosis of coronavirus disease 2019 (COVID-19). *Clin Chem Lab Med* 2020;58(7):1070-76. doi: 10.1515/cclm-2020-0285 [published Online First: 2020/03/17]

133. Liu X, Lv J, Gan L, et al. Comparative analysis of clinical characteristics, imaging and laboratory findings of different age groups with COVID-19. *Indian J Med Microbiol* 2020;38(1):87-93. doi: 10.4103/ijmm.IJMM_20_133 [published Online First: 2020/07/29]

134. López-Úbeda P, Díaz-Galiano MC, Martín-Noguerol T, et al. COVID-19 detection in radiological text reports integrating entity recognition. *Comput Biol Med* 2020;127:104066. doi: 10.1016/j.compbiomed.2020.104066 [published Online First: 2020/11/02]

135. Lübke N, Senff T, Scherger S, et al. Extraction-free SARS-CoV-2 detection by rapid RT-qPCR universal for all primary respiratory materials. *Journal of clinical virology : the official publication of the Pan American Society for Clinical Virology* 2020;130:104579. doi: 10.1016/j.jcv.2020.104579 [published Online First: 2020/08/17]

136. Mareev VY, Orlova YA, Plisyk AG, et al. Results of Open-Label non-Randomized Comparative Clinical Trial: "BromhexIne and Spironolactone for CoronаvirUs Infection requiring hospiTalization (BISCUIT). *Kardiologiia* 2020;60(11):4-15. doi: 10.18087/cardio.2020.11.n1440 [published Online First: 2021/01/26]

137. Matsumura K, Toyoda Y, Matsumoto S, et al. Comparison of the Clinical Course of COVID-19 Pneumonia and Acute Respiratory Distress Syndrome in 2 Passengers from the Cruise Ship Diamond Princess in February 2020. *The American journal of case reports* 2020;21:e926835. doi: 10.12659/ajcr.926835 [published Online First: 2020/08/20]

138. Monfardini L, Morassi M, Botti P, et al. Pulmonary thromboembolism in hospitalised COVID-19 patients at moderate to high risk by Wells score: a report from Lombardy, Italy. *The British journal of radiology* 2020;93(1113):20200407. doi: 10.1259/bjr.20200407 [published Online First: 2020/08/01]

139. Mostafa HH, Fissel JA, Fanelli B, et al. Metagenomic Next-Generation Sequencing of Nasopharyngeal Specimens Collected from Confirmed and Suspect COVID-19 Patients. *mBio* 2020;11(6) doi: 10.1128/mBio.01969-20 [published Online First: 2020/11/22]

140. Ota I, Asada Y. The impact of preoperative screening system on head and neck cancer surgery during the COVID-19 pandemic: Recommendations from the nationwide survey in Japan. *Auris Nasus Larynx* 2020;47(4):687-91. doi: 10.1016/j.anl.2020.05.006 [published Online First: 2020/05/20]

141. Pan Y, Long L, Zhang D, et al. Potential False-Negative Nucleic Acid Testing Results for Severe Acute Respiratory Syndrome Coronavirus 2 from Thermal Inactivation of Samples with Low Viral Loads. *Clin Chem* 2020;66(6):794-801. doi: 10.1093/clinchem/hvaa091 [published Online First: 2020/04/05]

142. Pham VH, Gargiulo Isacco C, Nguyen KCD, et al. Rapid and sensitive diagnostic procedure for multiple detection of pandemic Coronaviridae family members SARS-CoV-2, SARS-CoV, MERS-CoV and HCoV: a translational research and cooperation between the Phan Chau Trinh University in Vietnam and University of Bari "Aldo Moro" in Italy. *Eur Rev Med Pharmacol Sci* 2020;24(12):7173-91. doi: 10.26355/eurrev_202006_21713 [published Online First: 2020/07/08]

143. Rahmanzade R, Rahmanzadeh R, Hashemian SM. Respiratory Distress in Postanesthesia Care Unit: First Presentation of Coronavirus Disease 2019 in a 17-Year-Old Girl: A Case Report. *A&A practice* 2020;14(7):e01227. doi: 10.1213/xaa.0000000000001227 [published Online First: 2020/05/07]

144. Sabri A, Davarpanah AH, Mahdavi A, et al. Novel coronavirus disease 2019: predicting prognosis with a computed tomography-based disease severity score and clinical laboratory data. *Pol Arch Intern Med* 2020;130(7-8):629-34. doi: 10.20452/pamw.15422 [published Online First: 2020/06/06]

145. Safa O, Hassani-Azad M, Farashahinejad M, et al. Effects of Licorice on clinical symptoms and laboratory signs in moderately ill patients with pneumonia from COVID-19: A structured summary of a study protocol for a randomized controlled trial. *Trials* 2020;21(1):790. doi: 10.1186/s13063-020-04706-3 [published Online First: 2020/09/17]

146. Sahraian MA, Azimi A, Navardi S, et al. Evaluation of the rate of COVID-19 infection, hospitalization and death among Iranian patients with multiple sclerosis. *Mult Scler Relat Disord* 2020;46:102472. doi: 10.1016/j.msard.2020.102472 [published Online First: 2020/09/06]

147. Samec MJ, Khawaja A, Patel AM, et al. 80-year-old man with dyspnoea and bilateral groundglass infiltrates: an elusive case of COVID-19. *BMJ Case Rep* 2020;13(5) doi: 10.1136/bcr-2020-236069 [published Online First: 2020/05/30]

148. Schmid B, Feuerstein D, Lang CN, et al. Lung ultrasound in the emergency department - a valuable tool in the management of patients presenting with respiratory symptoms during the SARS-CoV-2 pandemic. *BMC Emerg Med* 2020;20(1):96. doi: 10.1186/s12873-020-00389-w [published Online First: 2020/12/09]

149. Serin I, Sari ND, Dogu MH, et al. A new parameter in COVID-19 pandemic: initial lactate dehydrogenase (LDH)/Lymphocyte ratio for diagnosis and mortality. *J Infect Public Health* 2020;13(11):1664-70. doi: 10.1016/j.jiph.2020.09.009 [published Online First: 2020/10/20]

150. Shao C, Liu H, Meng L, et al. Evolution of severe acute respiratory syndrome coronavirus 2 RNA test results in a patient with fatal coronavirus disease 2019: a case report. *Human pathology* 2020;101:82-88. doi: 10.1016/j.humpath.2020.04.015 [published Online First: 2020/05/22]

151. Shen C, Tan M, Song X, et al. Comparative Analysis of Early-Stage Clinical Features Between COVID-19 and Influenza A H1N1 Virus Pneumonia. *Front Public Health* 2020;8:206. doi: 10.3389/fpubh.2020.00206 [published Online First: 2020/06/24]

152. Siddiqui O, Manchanda V, Yadav A, et al. Comparison of two real-time polymerase chain reaction assays for the detection of severe acute respiratory syndrome-CoV-2 from combined nasopharyngeal-throat swabs. *Indian J Med Microbiol* 2020;38(3 & 4):385-89. doi: 10.4103/ijmm.IJMM_20_279 [published Online First: 2020/11/07]

153. Singanayagam A, Patel M, Charlett A, et al. Duration of infectiousness and correlation with RT-PCR cycle threshold values in cases of COVID-19, England, January to May 2020. *Euro surveillance : bulletin Europeen sur les maladies transmissibles = European communicable disease bulletin* 2020;25(32) doi: 10.2807/1560-7917.Es.2020.25.32.2001483 [published Online First: 2020/08/15]

154. Singh AK, Nema RK, Joshi A, et al. Evaluation of pooled sample analysis strategy in expediting case detection in areas with emerging outbreaks of COVID-19: A pilot study. *PLoS One* 2020;15(9):e0239492. doi: 10.1371/journal.pone.0239492 [published Online First: 2020/09/23]

155. Smith K, Pace A, Ortiz S, et al. A Phase 3 Open-label, Randomized, Controlled Study to Evaluate the Efficacy and Safety of Intravenously Administered Ravulizumab Compared with Best Supportive Care in Patients with COVID-19 Severe Pneumonia, Acute Lung Injury, or Acute Respiratory Distress Syndrome: A structured summary of a study protocol for a randomised controlled trial. *Trials* 2020;21(1):639. doi: 10.1186/s13063-020-04548-z [published Online First: 2020/07/15]

156. Soler-Luna C, Reynoso-Saldana D, Burgos MI, et al. Unexpected Ground-Glass Opacities on Abdominopelvic CT of a Patient With a Negative SARS-CoV-2 Antigen Test Result and No Respiratory Symptoms Upon Admission. *Cureus* 2020;12(10):e11044. doi: 10.7759/cureus.11044 [published Online First: 2020/10/27]

157. Su JW, Wu WR, Lang GJ, et al. Erratum to: Transmission risk of patients with COVID-19 meeting discharge criteria should be interpreted with caution. *Journal of Zhejiang University Science B* 2020;21(9):755. doi: 10.1631/jzus.B20e0117 [published Online First: 2020/09/08]

158. Tang S, Ou J, Li R, et al. Changes in CT manifestations and RT-PCR testings of the coronavirus disease 2019 until recovery in patients with afferent infection vs. second-generation infection outside the original city (Wuhan): An observational study. *Radiology of infectious diseases (Beijing, China)* 2020;7(3):123-29. doi: 10.1016/j.jrid.2020.07.007 [published Online First: 2020/08/25]

159. Tian M, Liu CC, Long L, et al. Management and Reconfiguration of a Radiology Department under the Threat of Coronavirus Disease 2019: Experience from Wuhan. *Curr Med Sci* 2020;40(4):608-13. doi: 10.1007/s11596-020-2223-z [published Online First: 2020/08/09]

160. Uechi T, Nakamura S, Takeshita R, et al. Persistence of positive severe acute respiratory syndrome coronavirus-2 reverse transcription-polymerase chain reaction test result for 24 days in a hospitalized asymptomatic carrier. *Acute medicine & surgery* 2020;7(1):e525. doi: 10.1002/ams2.525 [published Online First: 2020/06/13]

161. Walpole SC, McHugh R, Samuel J, et al. COVID-19 presenting as severe, persistent abdominal pain and causing late respiratory compromise in a 33-year-old man. *BMJ Case Rep* 2020;13(6) doi: 10.1136/bcr-2020-236030 [published Online First: 2020/06/18]

162. Wang R, He H, Liao C, et al. Clinical outcomes of hemodialysis patients infected with severe acute respiratory syndrome coronavirus 2 and impact of proactive chest computed tomography scans. *Clinical kidney journal* 2020;13(3):328-33. doi: 10.1093/ckj/sfaa086 [published Online First: 2020/07/23]

163. Wang Y, He Y, Tong J, et al. Characterization of an Asymptomatic Cohort of Severe Acute Respiratory Syndrome Coronavirus 2 (SARS-CoV-2) Infected Individuals Outside of Wuhan, China. *Clin Infect Dis* 2020;71(16):2132-38. doi: 10.1093/cid/ciaa629 [published Online First: 2020/05/23]

164. Xiao Y, Shi X, She Q, et al. Exploration of turn-positive RT-PCR results and factors related to treatment outcome in COVID-19: A retrospective cohort study. *Virulence* 2020;11(1):1250-56. doi: 10.1080/21505594.2020.1816076 [published Online First: 2020/09/15]

165. Chen R, Chen J, Meng Q-T. Chest computed tomography images of early coronavirus disease (COVID-19). *Canadian journal of anaesthesia = Journal canadien d'anesthesie* 2020 doi: 10.1007/s12630-020-01625-4

166. Chen L, Yuxiao D, Bin X, et al. Asymptomatic novel coronavirus pneumonia patient outside Wuhan: The value of CT images in the course of the disease. %J Clinical imaging. 2020;63

167. Qu J, Yang R, Song L, et al. Atypical lung feature on chest CT in a lung adenocarcinoma cancer patient infected with COVID-19. *Ann Oncol* 2020 doi: 10.1016/j.annonc.2020.03.001

168. Varble N, Blain M, Kassin M, et al. CT and clinical assessment in asymptomatic and pre-symptomatic patients with early SARS-CoV-2 in outbreak settings. *Eur Radiol* 2021;31(5):3165-76. doi: 10.1007/s00330-020-07401-8 [published Online First: 2020/11/05]

169. Özel M, Aslan A, Araç S. Use of the COVID-19 Reporting and Data System (CO-RADS) classification and chest computed tomography involvement score (CT-IS) in COVID-19 pneumonia. *Radiol Med* 2021;126(5):679-87. doi: 10.1007/s11547-021-01335-x [published Online First: 2021/02/14]

170. Mirahmadizadeh A, Pourmontaseri Z, Afrashteh S, et al. Sensitivity and specificity of chest computed tomography scan based on RT-PCR in COVID-19 diagnosis. *Pol J Radiol* 2021;86:e74-e77. doi: 10.5114/pjr.2021.103858 [published Online First: 2021/03/13]

171. Fonseca E, Assuncao Junior AN, Araujo-Filho JAB, et al. Lung Lesion Burden found on Chest CT as a Prognostic Marker in Hospitalized Patients with High Clinical Suspicion of COVID-19 Pneumonia: a Brazilian experience. *Clinics (Sao Paulo)* 2021;76:e3503. doi: 10.6061/clinics/2021/e3503 [published Online First: 2021/12/09]

172. Dai M, Ouyang L, Yang F, et al. Chest CT Imaging Features of Typical Covert COVID-19 Cases. *International journal of medical sciences* 2021;18(10):2128-36. doi: 10.7150/ijms.48614 [published Online First: 2021/04/17]

173. Xie S, Lei Z, Chen X, et al. Chest CT-based differential diagnosis of 28 patients with suspected corona virus disease 2019 (COVID-19). *The British journal of radiology* 2020;93(1112):20200243. doi: 10.1259/bjr.20200243 [published Online First: 2020/05/27]

174. Wang K, Kang S, Tian R, et al. Imaging manifestations and diagnostic value of chest CT of coronavirus disease 2019 (COVID-19) in the Xiaogan area. *Clin Radiol* 2020;75(5):341-47. doi: 10.1016/j.crad.2020.03.004 [published Online First: 2020/03/29]

175. Shah AS, Walkoff LA, Kuzo RS, et al. The utility of chest computed tomography (CT) and RT-PCR screening of asymptomatic patients for SARS-CoV-2 prior to semiurgent or urgent hospital procedures. *Infection control and hospital epidemiology* 2020;41(12):1375-77. doi: 10.1017/ice.2020.331 [published Online First: 2020/07/17]

176. de Jaegere TMH, Krdzalic J, Fasen B, et al. Radiological Society of North America Chest CT Classification System for Reporting COVID-19 Pneumonia: Interobserver Variability and Correlation with Reverse-Transcription Polymerase Chain Reaction. *Radiol Cardiothorac Imaging* 2020;2(3):e200213. doi: 10.1148/ryct.2020200213 [published Online First: 2021/03/30]

177. Baicry F, Le Borgne P, Fabacher T, et al. Patients with Initial Negative RT-PCR and Typical Imaging of COVID-19: Clinical Implications. *Journal of clinical medicine* 2020;9(9) doi: 10.3390/jcm9093014 [published Online First: 2020/09/24]

178. Borillo GA, Kagan RM, Baumann RE, et al. Pooling of Upper Respiratory Specimens Using a SARS-CoV-2 Real-time RT-PCR Assay Authorized for Emergency Use in Low-Prevalence Populations for High-Throughput Testing. *Open Forum Infect Dis* 2020;7(11):ofaa466. doi: 10.1093/ofid/ofaa466 [published Online First: 2020/11/19]

179. Chen LD, Li H, Ye YM, et al. A COVID-19 patient with multiple negative results for PCR assays outside Wuhan, China: a case report. *BMC Infect Dis* 2020;20(1):517. doi: 10.1186/s12879-020-05245-7 [published Online First: 2020/07/18]

180. Cho H, Jung YH, Cho HB, et al. Positive control synthesis method for COVID-19 diagnosis by one-step real-time RT-PCR. *Clin Chim Acta* 2020;511:149-53. doi: 10.1016/j.cca.2020.10.001 [published Online First: 2020/10/16]

181. Choudhuri J, Carter J, Nelson R, et al. SARS-CoV-2 PCR cycle threshold at hospital admission associated with patient mortality. *PLoS One* 2020;15(12):e0244777. doi: 10.1371/journal.pone.0244777 [published Online First: 2021/01/01]

182. de Salazar A, Aguilera A, Trastoy R, et al. Sample pooling for SARS-CoV-2 RT-PCR screening. *Clinical microbiology and infection : the official publication of the European Society of Clinical Microbiology and Infectious Diseases* 2020;26(12):1687.e1-87.e5. doi: 10.1016/j.cmi.2020.09.008 [published Online First: 2020/09/13]

183. Dorlass EG, Monteiro CO, Viana AO, et al. Lower cost alternatives for molecular diagnosis of COVID-19: conventional RT-PCR and SYBR Green-based RT-qPCR. *Braz J Microbiol* 2020;51(3):1117-23. doi: 10.1007/s42770-020-00347-5 [published Online First: 2020/08/09]

184. Dubbke-Laule A, Gnauck M, Straub R, et al. [Quadruple negative SARS-CoV-2-PCR: still COVID-19 pneumonia!]. *Deutsche medizinische Wochenschrift (1946)* 2020;145(20):1498-502. doi: 10.1055/a-1202-3936 [published Online First: 2020/09/02]

185. Dworzańska A, Tudrujek-Zdunek M, Mosiewicz J, et al. A 56-year-old man with RT-PCR negative nasopharyngeal swabs with Coronavirus Disease 2019 (COVID-19) Pneumonia. *Annals of agricultural and environmental medicine : AAEM* 2020;27(2):317-18. doi: 10.26444/aaem/123543 [published Online First: 2020/06/27]

186. Fan J, Yu F, Wang X, et al. Hock-a-loogie saliva as a diagnostic specimen for SARS-CoV-2 by a PCR-based assay: A diagnostic validity study. *Clin Chim Acta* 2020;511:177-80. doi: 10.1016/j.cca.2020.10.004 [published Online First: 2020/10/18]

187. Görgülü Ö, Duyan M. rRT-PCR Results of a Covid-19 Diagnosed Geriatric Patient. *SN Compr Clin Med* 2020;2(11):2423-26. doi: 10.1007/s42399-020-00590-9 [published Online First: 2020/10/27]

188. Hase R, Kurita T, Muranaka E, et al. A case of imported COVID-19 diagnosed by PCR-positive lower respiratory specimen but with PCR-negative throat swabs. *Infectious diseases (London, England)* 2020;52(6):423-26. doi: 10.1080/23744235.2020.1744711 [published Online First: 2020/04/03]

189. Hornuss D, Laubner K, Monasterio C, et al. [COVID-19 associated pneumonia despite repeatedly negative PCR-analysis from oropharyngeal swabs]. *Pneumologie* 2020;74(9):615-20. doi: 10.1055/a-1178-7275 [published Online First: 2020/09/12]

190. Hornuss D, Laubner K, Monasterio C, et al. [COVID-19 associated pneumonia despite repeatedly negative PCR-analysis from oropharyngeal swabs]. *Deutsche medizinische Wochenschrift (1946)* 2020;145(12):844-49. doi: 10.1055/a-1170-6061 [published Online First: 2020/05/14]

191. Hudowenz O, Klemm P, Lange U, et al. Case report of severe PCR-confirmed COVID-19 myocarditis in a European patient manifesting in mid January 2020. *European heart journal Case reports* 2020;4(6):1-6. doi: 10.1093/ehjcr/ytaa286 [published Online First: 2021/01/14]

192. Isikbay M, Henry TS, Frank JA, et al. When to rule out COVID-19: How many negative RT-PCR tests are needed? *Respir Med Case Rep* 2020;31:101192. doi: 10.1016/j.rmcr.2020.101192 [published Online First: 2020/08/25]

193. Khodamoradi Z, Hosseini SA, Gholampoor Saadi MH, et al. COVID-19 meningitis without pulmonary involvement with positive cerebrospinal fluid PCR. *Eur J Neurol* 2020;27(12):2668-69. doi: 10.1111/ene.14536 [published Online First: 2020/09/15]

194. Li Y, Yao L, Li J, et al. Stability issues of RT-PCR testing of SARS-CoV-2 for hospitalized patients clinically diagnosed with COVID-19. *J Med Virol* 2020;92(7):903-08. doi: 10.1002/jmv.25786 [published Online First: 2020/03/29]

195. Lv DF, Ying QM, Weng YS, et al. Dynamic change process of target genes by RT-PCR testing of SARS-Cov-2 during the course of a Coronavirus Disease 2019 patient. *Clin Chim Acta* 2020;506:172-75. doi: 10.1016/j.cca.2020.03.032 [published Online First: 2020/04/02]

196. Mohammed KS, de Laurent ZR, Omuoyo DO, et al. An optimization of four SARS-CoV-2 qRT-PCR assays in a Kenyan laboratory to support the national COVID-19 rapid response teams. *Wellcome open research* 2020;5:162. doi: 10.12688/wellcomeopenres.16063.2 [published Online First: 2022/03/29]

197. Nörz D, Frontzek A, Eigner U, et al. Pushing beyond specifications: Evaluation of linearity and clinical performance of the cobas 6800/8800 SARS-CoV-2 RT-PCR assay for reliable quantification in blood and other materials outside recommendations. *Journal of clinical virology : the official publication of the Pan American Society for Clinical Virology* 2020;132:104650. doi: 10.1016/j.jcv.2020.104650 [published Online First: 2020/10/05]

198. Opota O, Brouillet R, Greub G, et al. Comparison of SARS-CoV-2 RT-PCR on a high-throughput molecular diagnostic platform and the cobas SARS-CoV-2 test for the diagnostic of COVID-19 on various clinical samples. *Pathog Dis* 2020;78(8) doi: 10.1093/femspd/ftaa061 [published Online First: 2020/10/09]

199. Praharaj I, Jain A, Singh M, et al. Pooled testing for COVID-19 diagnosis by real-time RT-PCR: A multi-site comparative evaluation of 5- & 10-sample pooling. *Indian J Med Res* 2020;152(1 & 2):88-94. doi: 10.4103/ijmr.IJMR_2304_20 [published Online First: 2020/09/08]

200. Sahajpal NS, Mondal AK, Njau A, et al. Proposal of RT-PCR-Based Mass Population Screening for Severe Acute Respiratory Syndrome Coronavirus 2 (Coronavirus Disease 2019). *J Mol Diagn* 2020;22(10):1294-99. doi: 10.1016/j.jmoldx.2020.07.001 [published Online First: 2020/08/02]

201. Sakamaki I, Morinaga Y, Tani H, et al. Monitoring of viral load by RT-PCR caused decision making to continue ECMO therapy for a patient with COVID-19. *Journal of infection and chemotherapy : official journal of the Japan Society of Chemotherapy* 2020;26(12):1324-27. doi: 10.1016/j.jiac.2020.08.014 [published Online First: 2020/09/10]

202. Sarıgül F, Doluca O, Akhan S, et al. Investigation of compatibility of severe acute respiratory syndrome coronavirus 2 reverse transcriptase-PCR kits containing different gene targets during coronavirus disease 2019 pandemic. *Future Virol* 2020;15(8):515-24. doi: 10.2217/fvl-2020-0169 [published Online First: 2020/10/03]

203. Şık N, Özlü C, Karaoğlu Asrak H, et al. [Evaluation of SARS-CoV-2 PCR Positive Cases in the Pediatric Emergency Department]. *Mikrobiyol Bul* 2020;54(4):629-37. doi: 10.5578/mb.70086 [published Online First: 2020/10/28]

204. Szymczak WA, Goldstein DY, Orner EP, et al. Utility of Stool PCR for the Diagnosis of COVID-19: Comparison of Two Commercial Platforms. *J Clin Microbiol* 2020;58(9) doi: 10.1128/jcm.01369-20 [published Online First: 2020/07/03]

205. Ade C, Pum J, Abele I, et al. Analysis of cycle threshold values in SARS-CoV-2-PCR in a long-term study. *Journal of clinical virology : the official publication of the Pan American Society for Clinical Virology* 2021;138:104791. doi: 10.1016/j.jcv.2021.104791 [published Online First: 2021/03/17]

206. Al Qooz F, Behzad K, Louri N, et al. Efficiency of National Taskforce for Combating the Coronavirus (COVID-19) Protocol using real-time PCR testing in health facilities over a period of 8-weeks. *J Infect Public Health* 2021;14(8):1045-50. doi: 10.1016/j.jiph.2021.06.009 [published Online First: 2021/06/27]

207. Alcoba-Florez J, Gil-Campesino H, García-Martínez de Artola D, et al. Increasing SARS-CoV-2 RT-qPCR testing capacity by sample pooling. *International journal of infectious diseases : IJID : official publication of the International Society for Infectious Diseases* 2021;103:19-22. doi: 10.1016/j.ijid.2020.11.155 [published Online First: 2020/11/22]

208. Alouani DJ, Rajapaksha RRP, Jani M, et al. Specificity of SARS-CoV-2 Real-Time PCR Improved by Deep Learning Analysis. *J Clin Microbiol* 2021;59(6) doi: 10.1128/jcm.02959-20 [published Online First: 2021/03/19]

209. Aranha C, Patel V, Bhor V, et al. Cycle threshold values in RT-PCR to determine dynamics of SARS-CoV-2 viral load: An approach to reduce the isolation period for COVID-19 patients. *J Med Virol* 2021;93(12):6794-97. doi: 10.1002/jmv.27206 [published Online First: 2021/07/16]

210. Bakir A, Hosbul T, Cuce F, et al. Investigation of Viral Load Cycle Threshold Values in Patients with SARS-CoV-2 Associated Pneumonia with Real-Time PCR Method. *Journal of infection in developing countries* 2021;15(10):1408-14. doi: 10.3855/jidc.14281 [published Online First: 2021/11/16]

211. Brinkmann A, Ulm SL, Uddin S, et al. AmpliCoV: Rapid Whole-Genome Sequencing Using Multiplex PCR Amplification and Real-Time Oxford Nanopore MinION Sequencing Enables Rapid Variant Identification of SARS-CoV-2. *Frontiers in microbiology* 2021;12:651151. doi: 10.3389/fmicb.2021.651151 [published Online First: 2021/07/20]

212. Carroll A, McNamara E. Comparison and correlation of commercial SARS-CoV-2 real-time-PCR assays, Ireland, June 2020. *Euro surveillance : bulletin Europeen sur les maladies transmissibles = European communicable disease bulletin* 2021;26(6) doi: 10.2807/1560-7917.Es.2021.26.6.2002079 [published Online First: 2021/02/13]

213. Castineiras T, Nascimento É RDS, Faffe DS, et al. Performance of an alternative RT-PCR procedure using residual sample from the Panbio™ Ag COVID-19 test. *The Brazilian journal of infectious diseases : an official publication of the Brazilian Society of Infectious Diseases* 2021;25(5):101630. doi: 10.1016/j.bjid.2021.101630 [published Online First: 2021/10/15]

214. Claas ECJ, Smit PW, van Bussel M, et al. A two minute liquid based sample preparation for rapid SARS-CoV2 real-time PCR screening: A multicentre evaluation. *Journal of clinical virology : the official publication of the Pan American Society for Clinical Virology* 2021;135:104720. doi: 10.1016/j.jcv.2020.104720 [published Online First: 2021/01/09]

215. Cosgun Y, Altas AB, Kuzucu EA, et al. Role of rapid antibody and ELISA tests in the evaluation of serological response in patients with SARS-CoV-2 PCR positivity. *Folia Microbiol (Praha)* 2021;66(4):579-86. doi: 10.1007/s12223-021-00861-5 [published Online First: 2021/04/08]

216. Costa MS, Sato HI, Rocha RP, et al. Adjusting the Cut-Off and Maximum Pool Size in RT-qPCR Pool Testing for SARS-CoV-2. *Viruses* 2021;13(4) doi: 10.3390/v13040557 [published Online First: 2021/04/04]

217. Dash GC, Parai D, Choudhary HR, et al. SARS-CoV-2 IgG antibody responses in rt-PCR-positive cases: first report from India. *Access microbiology* 2021;3(10):000267. doi: 10.1099/acmi.0.000267 [published Online First: 2021/11/25]

218. Dierks S, Bader O, Schwanbeck J, et al. Diagnosing SARS-CoV-2 with Antigen Testing, Transcription-Mediated Amplification and Real-Time PCR. *Journal of clinical medicine* 2021;10(11) doi: 10.3390/jcm10112404 [published Online First: 2021/06/03]

219. Engelmann I, Alidjinou EK, Ogiez J, et al. Preanalytical Issues and Cycle Threshold Values in SARS-CoV-2 Real-Time RT-PCR Testing: Should Test Results Include These? *ACS Omega* 2021;6(10):6528-36. doi: 10.1021/acsomega.1c00166 [published Online First: 2021/03/23]

220. Fenaux H, Ghelfenstein-Ferreira T, Salmona M, et al. Interpretation of single target positivity among SARS-CoV-2 RT-PCR result tests. *Journal of clinical virology plus* 2021;1(1):100021. doi: 10.1016/j.jcvp.2021.100021 [published Online First: 2022/03/10]

221. Fitoussi F, Dupont R, Tonen-Wolyec S, et al. Performances of the VitaPCR™ SARS-CoV-2 Assay during the second wave of the COVID-19 epidemic in France. *J Med Virol* 2021;93(7):4351-57. doi: 10.1002/jmv.26950 [published Online First: 2021/03/20]

222. Fonseca E, Ferreira LC, Loureiro BMC, et al. Chest computed tomography in the diagnosis of COVID-19 in patients with false negative RT-PCR. *Einstein (Sao Paulo)* 2021;19:eAO6363. doi: 10.31744/einstein_journal/2021AO6363 [published Online First: 2021/11/11]

223. Garg A, Ghoshal U, Patel SS, et al. Evaluation of seven commercial RT-PCR kits for COVID-19 testing in pooled clinical specimens. *J Med Virol* 2021;93(4):2281-86. doi: 10.1002/jmv.26691 [published Online First: 2020/11/25]

224. Gentilini F, Turba ME, Taddei F, et al. Modelling RT-qPCR cycle-threshold using digital PCR data for implementing SARS-CoV-2 viral load studies. *PLoS One* 2021;16(12):e0260884. doi: 10.1371/journal.pone.0260884 [published Online First: 2021/12/21]

225. Gonçalves CCA, Barroso SPC, Herlinger AL, et al. COVID-19 diagnosis by RT-qPCR in alternative specimens. *Mem Inst Oswaldo Cruz* 2021;116:e210085. doi: 10.1590/0074-02760210085 [published Online First: 2021/08/19]

226. Gülbudak H, Karvar Ş, Soydan G, et al. [Comparison of Real Time PCR Cycle Threshold Values in Symptomatic and Asymptomatic COVID-19 Patients]. *Mikrobiyol Bul* 2021;55(3):435-44. doi: 10.5578/mb.20219812 [published Online First: 2021/08/22]

227. Handous I, Hannachi N, Marzouk M, et al. Pooling Nasopharyngeal Swab Specimens to Improve Testing Capacity for SARS-CoV-2 by Real-Time RT-PCR. *Biol Proced Online* 2021;23(1):19. doi: 10.1186/s12575-021-00156-6 [published Online First: 2021/10/02]

228. Ibrahim F, Natasha A, Saharman YR, et al. Consideration of the Cycle Threshold Values from Real-Time RT-PCR SARS-CoV-2 Interpretation for the Clinicians: Analysis of 339 Positive Cases from a Referral Laboratory in Jakarta, Indonesia. *Acta Med Indones* 2021;53(1):13-17. [published Online First: 2021/04/06]

229. Karimi F, Vaezi AA, Qorbani M, et al. Clinical and laboratory findings in COVID-19 adult hospitalized patients from Alborz province / Iran: comparison of rRT-PCR positive and negative. *BMC Infect Dis* 2021;21(1):256. doi: 10.1186/s12879-021-05948-5 [published Online First: 2021/03/13]

230. Kheiroddin P, Schöberl P, Althammer M, et al. Results of WICOVIR Gargle Pool PCR Testing in German Schools Based on the First 100,000 Tests. *Frontiers in pediatrics* 2021;9:721518. doi: 10.3389/fped.2021.721518 [published Online First: 2021/11/16]

231. Leber W, Lammel O, Siebenhofer A, et al. Comparing the diagnostic accuracy of point-of-care lateral flow antigen testing for SARS-CoV-2 with RT-PCR in primary care (REAP-2). *EClinicalMedicine* 2021;38:101011. doi: 10.1016/j.eclinm.2021.101011 [published Online First: 2021/07/20]

232. Mahanama A, Wilson-Davies E. Insight into PCR testing for surgeons. *Surgery (Oxford, Oxfordshire)* 2021;39(11):759-68. doi: 10.1016/j.mpsur.2021.09.016 [published Online First: 2021/11/02]

233. McNaughton CD, Adams NM, Hirschie Johnson C, et al. Diurnal Variation in SARS-CoV-2 PCR Test Results: Test Accuracy May Vary by Time of Day. *J Biol Rhythms* 2021;36(6):595-601. doi: 10.1177/07487304211051841 [published Online First: 2021/10/27]

234. More S, Narayanan S, Patil G, et al. Pooling of Nasopharyngeal Swab Samples To Overcome a Global Shortage of Real-Time Reverse Transcription-PCR COVID-19 Test Kits. *J Clin Microbiol* 2021;59(4) doi: 10.1128/jcm.01295-20 [published Online First: 2021/01/28]

235. Onwuamah CK, Okwuraiwe AP, Salu OB, et al. Comparative performance of SARS-CoV-2 real-time PCR diagnostic assays on samples from Lagos, Nigeria. *PLoS One* 2021;16(2):e0246637. doi: 10.1371/journal.pone.0246637 [published Online First: 2021/02/05]

236. Ota S, Sugawa S, Suematsu E, et al. Possibility of underestimation of COVID-19 prevalence by PCR and serological tests. *J Microbiol Immunol Infect* 2021 doi: 10.1016/j.jmii.2021.09.005 [published Online First: 2021/10/14]

237. Ozcan E, Yavuzer S, Borku Uysal B, et al. The relationship between positivity for COVID-19 RT-PCR and symptoms, clinical findings, and mortality in Turkey. *Expert Rev Mol Diagn* 2021;21(2):245-50. doi: 10.1080/14737159.2021.1882305 [published Online First: 2021/01/27]

238. Ozyurtlu F, Cetin N, Yavuz V. Drug-induced QTc interval prolongation in PCR-positive non-ICU COVID-19 patients with diverse findings on chest computed tomography. *Int J Clin Pract* 2021;75(10):e14583. doi: 10.1111/ijcp.14583 [published Online First: 2021/06/30]

239. Palacio Rua K, García Correa JF, Aguilar-Jiménez W, et al. Validation of a duplex PCR technique using the gen E and RNase P for the diagnosis of SARS-CoV-2. *Enfermedades infecciosas y microbiologia clinica (English ed)* 2021 doi: 10.1016/j.eimc.2020.12.014 [published Online First: 2021/02/24]

240. Park C, Lee J, Hassan ZU, et al. Comparison of Digital PCR and Quantitative PCR with Various SARS-CoV-2 Primer-Probe Sets. *J Microbiol Biotechnol* 2021;31(3):358-67. doi: 10.4014/jmb.2009.09006 [published Online First: 2021/01/06]

241. Peña M, Ampuero M, Garcés C, et al. Performance of SARS-CoV-2 rapid antigen test compared with real-time RT-PCR in asymptomatic individuals. *International journal of infectious diseases : IJID : official publication of the International Society for Infectious Diseases* 2021;107:201-04. doi: 10.1016/j.ijid.2021.04.087 [published Online First: 2021/05/05]

242. Potter RF, Abro B, Eby CS, et al. Evaluation of PCR cycle threshold values by patient population with the quidel lyra SARS-CoV-2 assay. *Diagn Microbiol Infect Dis* 2021;101(2):115387. doi: 10.1016/j.diagmicrobio.2021.115387 [published Online First: 2021/07/05]

243. Rahbari R, Moradi N, Abdi M. rRT-PCR for SARS-CoV-2: Analytical considerations. *Clin Chim Acta* 2021;516:1-7. doi: 10.1016/j.cca.2021.01.011 [published Online First: 2021/01/25]

244. Romero-Alvarez D, Garzon-Chavez D, Espinosa F, et al. Cycle Threshold Values in the Context of Multiple RT-PCR Testing for SARS-CoV-2. *Risk Manag Healthc Policy* 2021;14:1311-17. doi: 10.2147/rmhp.S282962 [published Online First: 2021/04/08]

245. Roquebert B, Haim-Boukobza S, Trombert-Paolantoni S, et al. SARS-CoV-2 variants of concern are associated with lower RT-PCR amplification cycles between January and March 2021 in France. *International journal of infectious diseases : IJID : official publication of the International Society for Infectious Diseases* 2021;113:12-14. doi: 10.1016/j.ijid.2021.09.076 [published Online First: 2021/10/04]

246. Silva Júnior JVJ, Merchioratto I, de Oliveira PSB, et al. End-point RT-PCR: A potential alternative for diagnosing coronavirus disease 2019 (COVID-19). *J Virol Methods* 2021;288:114007. doi: 10.1016/j.jviromet.2020.114007 [published Online First: 2020/11/02]

247. Singh J, Yadav AK, Pakhare A, et al. Comparative analysis of the diagnostic performance of five commercial COVID-19 qRT PCR kits used in India. *Scientific reports* 2021;11(1):22013. doi: 10.1038/s41598-021-00852-z [published Online First: 2021/11/12]

248. Soedarsono S, Febriani A, Hasan H, et al. Management of severe COVID-19 patient with negative RT-PCR for SARS-CoV-2: Role of clinical, radiological, and serological diagnosis. *Radiol Case Rep* 2021;16(6):1405-09. doi: 10.1016/j.radcr.2021.03.049 [published Online First: 2021/04/06]

249. Wagenhäuser I, Knies K, Rauschenberger V, et al. Clinical performance evaluation of SARS-CoV-2 rapid antigen testing in point of care usage in comparison to RT-qPCR. *EBioMedicine* 2021;69:103455. doi: 10.1016/j.ebiom.2021.103455 [published Online First: 2021/06/30]

250. Wang Z, Feng Z, Tang S, et al. Resurgence of Positive qRT-PCR Test Results in Patients Recovered from COVID-19: Case Reports. *The American journal of the medical sciences* 2021;361(5):650-54. doi: 10.1016/j.amjms.2021.01.019 [published Online First: 2021/05/25]

251. Waudby-West R, Parcell BJ, Palmer CNA, et al. The association between SARS-CoV-2 RT-PCR cycle threshold and mortality in a community cohort. *Eur Respir J* 2021;58(1) doi: 10.1183/13993003.00360-2021 [published Online First: 2021/06/27]

252. Yin N, Dellicour S, Daubie V, et al. Leveraging of SARS-CoV-2 PCR Cycle Thresholds Values to Forecast COVID-19 Trends. *Front Med (Lausanne)* 2021;8:743988. doi: 10.3389/fmed.2021.743988 [published Online First: 2021/11/19]

253. Yip CCY, Leung KH, Ng ACK, et al. Comparative evaluation of a dual-target real-time RT-PCR assay for COVID-19 diagnosis and assessment of performance in pooled saliva and nasopharyngeal swab samples. *Expert Rev Mol Diagn* 2021;21(7):741-47. doi: 10.1080/14737159.2021.1933445 [published Online First: 2021/05/21]

254. Yokota I, Hattori T, Shane PY, et al. Equivalent SARS-CoV-2 viral loads by PCR between nasopharyngeal swab and saliva in symptomatic patients. *Scientific reports* 2021;11(1):4500. doi: 10.1038/s41598-021-84059-2 [published Online First: 2021/02/26]

255. Zali A, Sohrabi MR, Mahdavi A, et al. Correlation Between Low-Dose Chest Computed Tomography and RT-PCR Results for the Diagnosis of COVID-19: A Report of 27,824 Cases in Tehran, Iran. *Acad Radiol* 2021;28(12):1654-61. doi: 10.1016/j.acra.2020.09.003 [published Online First: 2020/10/07]

256. Aijaz J, Naseer F, Dojki M, et al. Duration of respiratory sample stability at -80ºC for SARS-CoV-2 PCR. *Pak J Med Sci* 2022;38(2):393-98. doi: 10.12669/pjms.38.ICON-2022.5777 [published Online First: 2022/03/22]

257. Ayaz A, Demir AGO, Ozturk G, et al. A pooled RT-PCR testing strategy for more efficient COVID-19 pandemic management. *International journal of infectious diseases : IJID : official publication of the International Society for Infectious Diseases* 2022;116:1-6. doi: 10.1016/j.ijid.2021.12.328 [published Online First: 2021/12/19]

258. Baek YH, Park MY, Lim HJ, et al. Evaluation of Alternative Transport Media for RT-qPCR-Based SARS-CoV-2 Testing. *International journal of analytical chemistry* 2022;2022:5020255. doi: 10.1155/2022/5020255 [published Online First: 2022/08/23]

259. Beránek M, Koblížek V, Dulíček P, et al. Whole blood samples for faster real-time PCR analysis of thrombophilic mutations in SARS-CoV-2 virus positive patients. *Physiol Res* 2022;71(3):439-45. doi: 10.33549/physiolres.934883 [published Online First: 2022/05/27]

260. Buchta C, Camp JV, Jovanovic J, et al. A look at the precision, sensitivity and specificity of SARS-CoV-2 RT-PCR assays through a dedicated external quality assessment round. *Clin Chem Lab Med* 2022;60(2):e34-e37. doi: 10.1515/cclm-2021-1004 [published Online First: 2021/10/21]

261. Cuypers L, Bode J, Beuselinck K, et al. Nationwide Harmonization Effort for Semi-Quantitative Reporting of SARS-CoV-2 PCR Test Results in Belgium. *Viruses* 2022;14(6) doi: 10.3390/v14061294 [published Online First: 2022/06/25]

262. Ersoy L, Tezcan Ülger S, Gülbudak H, et al. [Longitudinal Monitoring of Seroconversion Status in SARS-CoV-2 RT-PCR Positive Healthcare Workers]. *Mikrobiyol Bul* 2022;56(1):114-23. doi: 10.5578/mb.20229910 [published Online First: 2022/01/29]

263. He Y, Xie T, Tu Q, et al. Importance of sample input volume for accurate SARS-CoV-2 qPCR testing. *Anal Chim Acta* 2022;1199:339585. doi: 10.1016/j.aca.2022.339585 [published Online First: 2022/03/02]

264. Javadi A, Dabiri S, Meymandi MS, et al. Changes of Routine Hematological Parameters in COVID-19 Patients: Correlation with Imaging Findings, RT-PCR and Outcome. *Iranian journal of pathology* 2022;17(1):37-47. doi: 10.30699/ijp.2021.533645.2675 [published Online First: 2022/02/01]

265. Keskin M, Polat SB, Ates İ, et al. Is There Any Correlation between Baseline Serum Cortisol Levels and Disease Severity in PCR-Positive COVID-19 Patients with and without Diabetes Mellitus? *Vaccines (Basel)* 2022;10(8) doi: 10.3390/vaccines10081361 [published Online First: 2022/08/27]

266. Kogoj R, Korva M, Knap N, et al. Comparative Evaluation of Six SARS-CoV-2 Real-Time RT-PCR Diagnostic Approaches Shows Substantial Genomic Variant-Dependent Intra- and Inter-Test Variability, Poor Interchangeability of Cycle Threshold and Complementary Turn-Around Times. *Pathogens* 2022;11(4) doi: 10.3390/pathogens11040462 [published Online First: 2022/04/24]

267. Leventopoulos M, Michou V, Papadimitropoulos M, et al. Evaluation of the Boson rapid Ag test vs RT-PCR for use as a self-testing platform. *Diagn Microbiol Infect Dis* 2022;104(3):115786. doi: 10.1016/j.diagmicrobio.2022.115786 [published Online First: 2022/08/24]

268. Li RH, Wang QY. A localized small-scale external quality assessment (EQA) for PCR testing of severe acute respiratory syndrome coronavirus 2 (SARS-CoV-2) in the molecular laboratories. *J Virol Methods* 2022;301:114441. doi: 10.1016/j.jviromet.2021.114441 [published Online First: 2021/12/27]

269. Martínez MJ, Basile L, Sisó-Almirall A, et al. Lack of Prognostic Value of SARS-CoV2 RT-PCR Cycle Threshold in the Community. *Infect Dis Ther* 2022;11(1):587-93. doi: 10.1007/s40121-021-00561-0 [published Online First: 2021/11/12]

270. Oba J, Taniguchi H, Sato M, et al. SARS-CoV-2 RT-qPCR testing of pooled saliva samples: A case study of 824 asymptomatic individuals and a questionnaire survey in Japan. *PLoS One* 2022;17(5):e0263700. doi: 10.1371/journal.pone.0263700 [published Online First: 2022/05/14]

271. Özkarafakılı MA, Özkurt H, Bardakçı M, et al. Comparison of chest computed tomography findings of RT-PCR negative and RT-PCR positive cases in COVID-19 patients. *Clinical imaging* 2022;82:7-12. doi: 10.1016/j.clinimag.2021.10.013 [published Online First: 2021/11/13]

272. Palacio Rua K, García Correa JF, Aguilar-Jiménez W, et al. Validation of a duplex PCR technique using the gen E and RNase P for the diagnosis of SARS-CoV-2. *Enfermedades infecciosas y microbiologia clinica (English ed)* 2022 doi: 10.1016/j.eimce.2022.05.005 [published Online First: 2022/06/02]

273. Park K, Sung H, Chun S, et al. Evaluation of Intra- and Interlaboratory Variations in SARS-CoV-2 Real-Time RT-PCR Through Nationwide Proficiency Testing. *Lab Med* 2022 doi: 10.1093/labmed/lmac052 [published Online First: 2022/06/15]

274. Popping S, Molenkamp R, Weigel JD, et al. Diminished amplification of SARS-CoV-2 ORF1ab in a commercial dual-target qRT-PCR diagnostic assay. *J Virol Methods* 2022;300:114397. doi: 10.1016/j.jviromet.2021.114397 [published Online First: 2021/12/06]

275. Quiroz-Ruiz HR, Chimoy-Effio PJ, Vértiz-Osores JJ, et al. [Correlation between real-time PCR cycle threshold and clinical classification of COVID-19]. *Rev Chilena Infectol* 2022;39(1):35-44. doi: 10.4067/s0716-10182022000100035 [published Online First: 2022/06/24]

276. Ratcliff J, Al-Beidh F, Bibi S, et al. Highly Sensitive Lineage Discrimination of SARS-CoV-2 Variants through Allele-Specific Probe PCR. *J Clin Microbiol* 2022;60(4):e0228321. doi: 10.1128/jcm.02283-21 [published Online First: 2022/03/25]

277. Sahoo MK, Huang C, Sibai M, et al. Harmonization of SARS-CoV-2 reverse transcription quantitative PCR tests to the first WHO international standard for SARS-CoV-2 RNA. *Journal of clinical virology : the official publication of the Pan American Society for Clinical Virology* 2022;154:105242. doi: 10.1016/j.jcv.2022.105242 [published Online First: 2022/08/10]

278. Seetha D, Ravikumar A, Nair RR. Comparative performance of CDC-modified SARS-CoV-2 real-time PCR assay with four different commercial assays: laboratory-based study. *Comparative clinical pathology* 2022;31(3):355-63. doi: 10.1007/s00580-022-03356-y [published Online First: 2022/06/01]

279. Sung A, Bailey AL, Stewart HB, et al. Isolation of SARS-CoV-2 in Viral Cell Culture in Immunocompromised Patients With Persistently Positive RT-PCR Results. *Frontiers in cellular and infection microbiology* 2022;12:804175. doi: 10.3389/fcimb.2022.804175 [published Online First: 2022/02/22]

280. Tré-Hardy M, Piteüs S, Beukinga I, et al. Clinical evaluation of the GSD NovaPrime® SARS-CoV-2 RTq-PCR assay. *Diagn Microbiol Infect Dis* 2022;103(3):115718. doi: 10.1016/j.diagmicrobio.2022.115718 [published Online First: 2022/06/01]

281. Vierbaum L, Wojtalewicz N, Grunert HP, et al. RNA reference materials with defined viral RNA loads of SARS-CoV-2-A useful tool towards a better PCR assay harmonization. *PLoS One* 2022;17(1):e0262656. doi: 10.1371/journal.pone.0262656 [published Online First: 2022/01/21]

282. Whale AS, von der Heide EK, Kohlenberg M, et al. Digital PCR can augment the interpretation of RT-qPCR Cq values for SARS-CoV-2 diagnostics. *Methods (San Diego, Calif)* 2022;201:5-14. doi: 10.1016/j.ymeth.2021.08.006 [published Online First: 2021/08/29]

283. Lee EYP, Ng MY, Khong PL. COVID-19 pneumonia: what has CT taught us? *Lancet Infect Dis* 2020 doi: 10.1016/s1473-3099(20)30134-1 [published Online First: 2020/02/28]

284. Li W, Cui H, Li K, et al. Chest computed tomography in children with COVID-19 respiratory infection. *Pediatric radiology* 2020 doi: 10.1007/s00247-020-04656-7

285. Yang D, Xu Z, Li W, et al. Federated semi-supervised learning for COVID region segmentation in chest CT using multi-national data from China, Italy, Japan. *Med Image Anal* 2021;70:101992. doi: 10.1016/j.media.2021.101992 [published Online First: 2021/02/19]

286. Schuster P, Crombé A, Nivet H, et al. Practical clinical and radiological models to diagnose COVID-19 based on a multicentric teleradiological emergency chest CT cohort. *Scientific reports* 2021;11(1):8994. doi: 10.1038/s41598-021-88053-6 [published Online First: 2021/04/28]

287. Salem Salamh AB, Salamah AA, Akyüz HI. A Study of a New Technique of the CT Scan View and Disease Classification Protocol Based on Level Challenges in Cases of Coronavirus Disease. *Radiol Res Pract* 2021;2021:5554408. doi: 10.1155/2021/5554408 [published Online First: 2021/04/02]

288. Perumal V, Narayanan V, Rajasekar SJS. Prediction of COVID-19 with Computed Tomography Images using Hybrid Learning Techniques. *Dis Markers* 2021;2021:5522729. doi: 10.1155/2021/5522729 [published Online First: 2021/05/11]

289. Mishra S. Deep Transfer Learning-Based Framework for COVID-19 Diagnosis Using Chest CT Scans and Clinical Information. *SN Comput Sci* 2021;2(5):390. doi: 10.1007/s42979-021-00785-4 [published Online First: 2021/08/03]

290. Lu W, Wei J, Xu T, et al. Quantitative CT for detecting COVID19 pneumonia in suspected cases. *BMC Infect Dis* 2021;21(1):836. doi: 10.1186/s12879-021-06556-z [published Online First: 2021/08/21]

291. Liu B, Liu P, Dai L, et al. Assisting scalable diagnosis automatically via CT images in the combat against COVID-19. *Scientific Reports* 2021;11(1) doi: 10.1038/s41598-021-83424-5

292. Lacerda P, Barros B, Albuquerque C, et al. Hyperparameter Optimization for COVID-19 Pneumonia Diagnosis Based on Chest CT. *Sensors (Basel, Switzerland)* 2021;21(6) doi: 10.3390/s21062174 [published Online First: 2021/04/04]

293. Kumar Singh V, Abdel-Nasser M, Pandey N, et al. LungINFseg: Segmenting COVID-19 Infected Regions in Lung CT Images Based on a Receptive-Field-Aware Deep Learning Framework. *Diagnostics (Basel)* 2021;11(2) doi: 10.3390/diagnostics11020158 [published Online First: 2021/01/28]

294. Heidarian S, Afshar P, Enshaei N, et al. COVID-FACT: A Fully-Automated Capsule Network-Based Framework for Identification of COVID-19 Cases from Chest CT Scans. *Front Artif Intell* 2021;4:598932. doi: 10.3389/frai.2021.598932 [published Online First: 2021/06/12]

295. Han CH, Kim M, Kwak JT. Semi-supervised learning for an improved diagnosis of COVID-19 in CT images. *PLoS One* 2021;16(4):e0249450. doi: 10.1371/journal.pone.0249450 [published Online First: 2021/04/02]

296. Chen X, Yao L, Zhou T, et al. Momentum contrastive learning for few-shot COVID-19 diagnosis from chest CT images. *Pattern Recognit* 2021;113:107826. doi: 10.1016/j.patcog.2021.107826 [published Online First: 2021/02/02]

297. Yan T, Wong PK, Ren H, et al. Automatic distinction between COVID-19 and common pneumonia using multi-scale convolutional neural network on chest CT scans. *Chaos Solitons Fractals* 2020;140:110153. doi: 10.1016/j.chaos.2020.110153 [published Online First: 2020/08/25]

298. Warman A, Warman P, Sharma A, et al. Interpretable Artificial Intelligence for COVID-19 Diagnosis from Chest CT Reveals Specificity of Ground-Glass Opacities. *medRxiv* 2020

299. Steuwe A, Rademacher C, Valentin B, et al. Dose-optimised chest computed tomography for diagnosis of Coronavirus Disease 2019 (COVID-19) - Evaluation of image quality and diagnostic impact. *J Radiol Prot* 2020;40(3):877-91. doi: 10.1088/1361-6498/aba16a [published Online First: 2020/07/01]

300. Singh D, Kumar V, Vaishali, et al. Classification of COVID-19 patients from chest CT images using multi-objective differential evolution-based convolutional neural networks. *Eur J Clin Microbiol Infect Dis* 2020;39(7):1379-89. doi: 10.1007/s10096-020-03901-z [published Online First: 2020/04/28]

301. Panwar H, Gupta PK, Siddiqui MK, et al. A deep learning and grad-CAM based color visualization approach for fast detection of COVID-19 cases using chest X-ray and CT-Scan images. *Chaos Solitons Fractals* 2020;140:110190. doi: 10.1016/j.chaos.2020.110190 [published Online First: 2020/08/25]

302. Mishra AK, Das SK, Roy P, et al. Identifying COVID19 from Chest CT Images: A Deep Convolutional Neural Networks Based Approach. *J Healthc Eng* 2020;2020:8843664. doi: 10.1155/2020/8843664 [published Online First: 2020/08/25]

303. Guiot J, Vaidyanathan A, Deprez L, et al. Development and Validation of an Automated Radiomic CT Signature for Detecting COVID-19. *Diagnostics (Basel)* 2020;11(1) doi: 10.3390/diagnostics11010041 [published Online First: 2021/01/06]

304. Gezer NS, Ergan B, Baris MM, et al. COVID-19 S: A new proposal for diagnosis and structured reporting of COVID-19 on computed tomography imaging. *Diagn Interv Radiol* 2020;26(4):315-22. doi: 10.5152/dir.2020.20351 [published Online First: 2020/06/20]

305. Cai W, Liu T, Xue X, et al. CT Quantification and Machine-learning Models for Assessment of Disease Severity and Prognosis of COVID-19 Patients. *Acad Radiol* 2020;27(12):1665-78. doi: 10.1016/j.acra.2020.09.004 [published Online First: 2020/10/14]

306. Attallah O, Ragab DA, Sharkas M. MULTI-DEEP: A novel CAD system for coronavirus (COVID-19) diagnosis from CT images using multiple convolution neural networks. *PeerJ* 2020;8:e10086. doi: 10.7717/peerj.10086 [published Online First: 2020/10/17]

307. Sarkodie BD, Mensah YB. CT scan chest findings in symptomatic COVID-19 patients: a reliable alternative for diagnosis. *Ghana Med J* 2020;54(4 Suppl):97-99. doi: 10.4314/gmj.v54i4s.14 [published Online First: 2021/05/13]

308. Zeng QQ, Zheng KI, Chen J, et al. Radiomics-based model for accurately distinguishing between severe acute respiratory syndrome associated coronavirus 2 (SARS-CoV-2) and influenza A infected pneumonia. *MedComm* 2020;1(2):240-48. doi: 10.1002/mco2.14 [published Online First: 2020/08/25]

309. Zhang B, Zhang J, Chen H, et al. Novel coronavirus disease 2019 (COVID-19): relationship between chest CT scores and laboratory parameters. *Eur J Nucl Med Mol Imaging* 2020;47(9):2083-89. doi: 10.1007/s00259-020-04854-3 [published Online First: 2020/05/14]

310. Zhao D, Yao F, Wang L, et al. A Comparative Study on the Clinical Features of Coronavirus 2019 (COVID-19) Pneumonia With Other Pneumonias. *Clin Infect Dis* 2020;71(15):756-61. doi: 10.1093/cid/ciaa247 [published Online First: 2020/03/13]

311. Zhong Q, Liu YY, Luo Q, et al. Spinal anaesthesia for patients with coronavirus disease 2019 and possible transmission rates in anaesthetists: retrospective, single-centre, observational cohort study. *British journal of anaesthesia* 2020;124(6):670-75. doi: 10.1016/j.bja.2020.03.007 [published Online First: 2020/04/03]

312. Zhou L, Li Z, Zhou J, et al. A Rapid, Accurate and Machine-Agnostic Segmentation and Quantification Method for CT-Based COVID-19 Diagnosis. *IEEE Trans Med Imaging* 2020;39(8):2638-52. doi: 10.1109/tmi.2020.3001810 [published Online First: 2020/07/31]

313. Ziaei A, Davoodian P, Dadvand H, et al. Evaluation of the efficacy and safety of Melatonin in moderately ill patients with COVID-19: A structured summary of a study protocol for a randomized controlled trial. *Trials* 2020;21(1):882. doi: 10.1186/s13063-020-04737-w [published Online First: 2020/10/28]

314. Zieleskiewicz L, Markarian T, Lopez A, et al. Comparative study of lung ultrasound and chest computed tomography scan in the assessment of severity of confirmed COVID-19 pneumonia. *Intensive Care Med* 2020;46(9):1707-13. doi: 10.1007/s00134-020-06186-0 [published Online First: 2020/07/31]

315. Abdel-Hamid HM, Rizk HI, Magdy S. Occurrence of pulmonary residuals as one of the sequelae of COVID-19 and it's predictors among moderate and severe cases. *The Indian journal of tuberculosis* 2021;68(4):450-56. doi: 10.1016/j.ijtb.2021.01.006 [published Online First: 2021/11/10]

316. Akpinar G, Demir MC, Sultanoglu H, et al. Comparison of Clinical, Laboratory and Demographic Characteristics of Patients Diagnosed with COVID-19 as Symptomatic and Atypical Symptoms. *Clin Lab* 2021;67(5) doi: 10.7754/Clin.Lab.2020.200929 [published Online First: 2021/05/13]

317. Akyüz A, Özçağlayan Tİ K, Şahin GG, et al. Some inflammatory markers and chest computerized tomography in patients with severe acute respiratory syndrome coronavirus infection. *Rev Assoc Med Bras (1992)* 2021;67(4):522-28. doi: 10.1590/1806-9282.20200938 [published Online First: 2021/09/09]

318. Al-Kindi N, Al-Shukri I, Al-Rashdi A, et al. Validation of GeneXpert testing for SARS-CoV-2 RNA in eight hospital laboratories in Oman. *Pan Afr Med J* 2021;40:2. doi: 10.11604/pamj.2021.40.2.27224 [published Online First: 2021/10/16]

319. Arnaout R, Lee RA, Lee GR, et al. The Limit of Detection Matters: The Case for Benchmarking Severe Acute Respiratory Syndrome Coronavirus 2 Testing. *Clin Infect Dis* 2021;73(9):e3042-e46. doi: 10.1093/cid/ciaa1382 [published Online First: 2021/02/04]

320. Arora R, Goel R, Kumar S, et al. Evaluation of SARS-CoV-2 in Tears of Patients with Moderate to Severe COVID-19. *Ophthalmology* 2021;128(4):494-503. doi: 10.1016/j.ophtha.2020.08.029 [published Online First: 2020/09/04]

321. Arora R, Goel R, Saxena S, et al. Comparative Evaluation of Tears and Nasopharyngeal Swab for SARS-CoV-2 in COVID-19 Dedicated Intensive Care Unit Patients. *Ocular immunology and inflammation* 2021;29(4):690-96. doi: 10.1080/09273948.2021.1903938 [published Online First: 2021/05/12]

322. Asano Y, Koshi T, Sano A, et al. A patient with mild respiratory COVID-19 infection who developed bilateral non-hemorrhagic adrenal infarction. *Nagoya J Med Sci* 2021;83(4):883-91. doi: 10.18999/nagjms.83.4.883 [published Online First: 2021/12/18]

323. Ashraf S, Ashraf S, Farooq I, et al. Anti-COVID property of subcutaneous ivermectin in synergy with zinc among midlife moderately symptomatic patients: a structured summary of a study protocol for a randomised controlled trial. *Trials* 2021;22(1):591. doi: 10.1186/s13063-021-05487-z [published Online First: 2021/09/08]

324. da Costa CM, de Souza ZS, Real Salgues AC, et al. COVID-19 in a patient with advanced Merkel cell carcinoma receiving immunotherapy. *Immunotherapy* 2020;12(15):1133-38. doi: 10.2217/imt-2020-0193 [published Online First: 2020/09/10]

325. d'Ettorre G, Recchia G, Ridolfi M, et al. Analysis of type I IFN response and T cell activation in severe COVID-19/HIV-1 coinfection: A case report. *Medicine (Baltimore)* 2020;99(36):e21803. doi: 10.1097/md.0000000000021803 [published Online First: 2020/09/10]

326. Han M, Xu M, Zhang Y, et al. Assessing SARS-CoV-2 RNA levels and lymphocyte/T cell counts in COVID-19 patients revealed initial immune status as a major determinant of disease severity. *Med Microbiol Immunol* 2020;209(6):657-68. doi: 10.1007/s00430-020-00693-z [published Online First: 2020/08/30]

327. Hosoba R, Makita S, Shiotsuka M, et al. COVID-19 pneumonia in a patient with adult T-cell leukemia-lymphoma. *J Clin Exp Hematop* 2020;60(4):174-78. doi: 10.3960/jslrt.20030 [published Online First: 2020/09/04]

328. La Scola B, Le Bideau M, Andreani J, et al. Viral RNA load as determined by cell culture as a management tool for discharge of SARS-CoV-2 patients from infectious disease wards. *European journal of clinical microbiology & infectious diseases : official publication of the European Society of Clinical Microbiology* 2020;39(6):1059-61. doi: 10.1007/s10096-020-03913-9 [published Online First: 2020/04/29]

329. Lancman G, Mascarenhas J, Bar-Natan M. Severe COVID-19 virus reactivation following treatment for B cell acute lymphoblastic leukemia. *Journal of hematology & oncology* 2020;13(1):131. doi: 10.1186/s13045-020-00968-1 [published Online First: 2020/10/04]

330. Liang B, Chen J, Li T, et al. Clinical remission of a critically ill COVID-19 patient treated by human umbilical cord mesenchymal stem cells: A case report. *Medicine (Baltimore)* 2020;99(31):e21429. doi: 10.1097/md.0000000000021429 [published Online First: 2020/08/07]

331. Nyayanit DA, Sarkale P, Baradkar S, et al. Transcriptome & viral growth analysis of SARS-CoV-2-infected Vero CCL-81 cells. *Indian J Med Res* 2020;152(1 & 2):70-76. doi: 10.4103/ijmr.IJMR_2257_20 [published Online First: 2020/08/11]

332. Osman J, Lambert J, Templé M, et al. Rapid screening of COVID-19 patients using white blood cell scattergrams, a study on 381 patients. *Br J Haematol* 2020;190(5):718-22. doi: 10.1111/bjh.16943 [published Online First: 2020/06/17]

333. Daher A, Müller T, Spiesshoefer J, et al. Successful treatment of prolonged COVID-19 with Bamlanivimab in a patient with severe B-Cell aplasia due to treatment with an anti-CD20 monoclonal antibody: A case report. *Respir Med Case Rep* 2021;34:101560. doi: 10.1016/j.rmcr.2021.101560 [published Online First: 2021/12/07]

334. Han A, Rodriguez TE, Beck ET, et al. Persistent SARS-CoV-2 infectivity greater than 50 days in a case series of allogeneic peripheral blood stem cell transplant recipients. *Current problems in cancer Case reports* 2021;3:100057. doi: 10.1016/j.cpccr.2021.100057 [published Online First: 2021/07/27]

335. Hashemian SM, Shafigh N, Afzal G, et al. Plasmapheresis reduces cytokine and immune cell levels in COVID-19 patients with acute respiratory distress syndrome (ARDS). *Pulmonology* 2021;27(6):486-92. doi: 10.1016/j.pulmoe.2020.10.017 [published Online First: 2020/12/29]

336. Kaparou M, Rudzki Z, Giles H, et al. Management of Allogeneic Stem Cell Transplantation for High-Risk AML following SARS-CoV-2 Associated Pancytopenia with Marked Bone Marrow Biopsy Alterations. *Case Rep Hematol* 2021;2021:8843063. doi: 10.1155/2021/8843063 [published Online First: 2021/01/30]

337. Kriegova E, Fillerova R, Raska M, et al. Excellent option for mass testing during the SARS-CoV-2 pandemic: painless self-collection and direct RT-qPCR. *Virol J* 2021;18(1):95. doi: 10.1186/s12985-021-01567-3 [published Online First: 2021/05/06]

338. Steinlin-Schopfer J, Barbani MT, Kamgang R, et al. Evaluation of the Roche antigen rapid test and a cell culture-based assay compared to rRT- PCR for the detection of SARS-CoV-2: A contribution to the discussion about SARS-CoV-2 diagnostic tests and contagiousness. *Journal of clinical virology plus* 2021;1(1):100020. doi: 10.1016/j.jcvp.2021.100020 [published Online First: 2022/03/10]

339. Vasse M, Ballester MC, Ayaka D, et al. Interest of the cellular population data analysis as an aid in the early diagnosis of SARS-CoV-2 infection. *Int J Lab Hematol* 2021;43(1):116-22. doi: 10.1111/ijlh.13312 [published Online First: 2020/08/20]

340. Xiao H, Luo Y, Shi J, et al. How Do We Manage Hematopoietic Cell Transplant during the SARS-CoV-2 Pandemic? *Acta Haematol* 2021;144(5):500-07. doi: 10.1159/000513036 [published Online First: 2021/03/11]

341. Yamada S, Fukushi S, Kinoshita H, et al. Assessment of SARS-CoV-2 infectivity of upper respiratory specimens from COVID-19 patients by virus isolation using VeroE6/TMPRSS2 cells. *BMJ Open Respir Res* 2021;8(1) doi: 10.1136/bmjresp-2020-000830 [published Online First: 2021/02/26]

342. Yilmaz F, Yasar S, Tuncali MC, et al. Complete response in a frail patient with high-grade B-cell lymphoma to only one cycle of R-CHOP or to prolonged COVID-19? *Semin Oncol* 2021;48(4-6):279-82. doi: 10.1053/j.seminoncol.2021.11.001 [published Online First: 2021/12/14]

343. Zahran AM, Zahran ZAM, Mady YH, et al. Differential alterations in peripheral lymphocyte subsets in COVID-19 patients: upregulation of double-positive and double-negative T cells. *Multidiscip Respir Med* 2021;16(2):758. doi: 10.4081/mrm.2021.758 [published Online First: 2021/07/06]

344. Auerswald H, Low DHW, Siegers JY, et al. A Look inside the Replication Dynamics of SARS-CoV-2 in Blyth's Horseshoe Bat (Rhinolophus lepidus) Kidney Cells. *Microbiol Spectr* 2022;10(3):e0044922. doi: 10.1128/spectrum.00449-22 [published Online First: 2022/06/01]

345. Gabr H, Bastawy S, Abdel Aal AA, et al. Changes in peripheral blood cellular morphology as diagnostic markers for COVID-19 infection. *Int J Lab Hematol* 2022;44(3):454-60. doi: 10.1111/ijlh.13799 [published Online First: 2022/01/21]

346. Göhler F, Corman VM, Bleicker T, et al. Contamination of CT scanner surfaces with SARS-CoV-2 and infective potential after examination of invasively ventilated, non-invasively ventilated and non-ventilated patients with positive throat swabs: prospective investigation using real-time reverse-transcription PCR and viral cell culture. *Insights Imaging* 2022;13(1):61. doi: 10.1186/s13244-022-01202-x [published Online First: 2022/03/30]

347. Gül Ö, Binay UD, Barkay O, et al. [The Importance of Differential Diagnosis During Pandemic: A Case Report with Coexistence of COVID-19, Brucellosis and Crimean-Congo Hemorrhagic Fever]. *Mikrobiyol Bul* 2022;56(2):365-70. doi: 10.5578/mb.20229815 [published Online First: 2022/04/29]

348. Jungnick S, Hobmaier B, Paravinja N, et al. Analysis of seven SARS-CoV-2 rapid antigen tests in detecting omicron (B.1.1.529) versus delta (B.1.617.2) using cell culture supernatants and clinical specimens. *Infection* 2022:1-7. doi: 10.1007/s15010-022-01844-5 [published Online First: 2022/05/21]

349. Moradians V, Shateri Amiri B, Bahadorizadeh L, et al. Concurrent COVID-19 and pneumocystis carinii pneumonia in a patient subsequently found to have underlying hairy cell leukemia. *Radiol Case Rep* 2022;17(9):3238-42. doi: 10.1016/j.radcr.2022.06.026 [published Online First: 2022/07/08]

350. Payandeh M, Habibi R, Norooznezhad AH, et al. Human placenta-derived mesenchymal stromal cells transfusion in a critically Ill infant diagnosed with Coronavirus Disease 2019 (COVID-19): A case report. *Transfusion and apheresis science : official journal of the World Apheresis Association : official journal of the European Society for Haemapheresis* 2022:103454. doi: 10.1016/j.transci.2022.103454 [published Online First: 2022/05/27]

351. Sartaj Sohrab S, Aly El-Kafrawy S, Ibraheem Azhar E. In silico prediction and experimental evaluation of potential siRNAs against SARS-CoV-2 inhibition in Vero E6 cells. *Journal of King Saud University Science* 2022;34(4):102049. doi: 10.1016/j.jksus.2022.102049 [published Online First: 2022/05/03]

352. Shirazi Tehrani A, Tabatabaei Mirakabad FS, Abdollahifar MA, et al. Severe Acute Respiratory Syndrome Coronavirus 2 Induces Hepatocyte Cell Death, Active Autophagosome Formation and Caspase 3 Up-Regulation in Postmortem Cases: Stereological and Molecular Study. *Tohoku J Exp Med* 2022;256(4):309-19. doi: 10.1620/tjem.2022.J007 [published Online First: 2022/03/25]

353. Sohrab SS, El-Kafrawy SA, Azhar EI. Effect of insilico predicted and designed potential siRNAs on inhibition of SARS-CoV-2 in HEK-293 cells. *Journal of King Saud University Science* 2022;34(4):101965. doi: 10.1016/j.jksus.2022.101965 [published Online First: 2022/03/23]

354. Totschnig D, Doberer D, Haberl R, et al. Treatment of persistent COVID-19 in two B-cell-depleted patients with the monoclonal antibody Sotrovimab. *IDCases* 2022;29:e01528. doi: 10.1016/j.idcr.2022.e01528 [published Online First: 2022/06/14]

355. Funt SA, Cohen SL, Wang JJ, et al. Abdominal pelvic CT findings compared between COVID-19 positive and COVID-19 negative patients in the emergency department setting. *Abdominal radiology (New York)* 2021;46(4):1498-505. doi: 10.1007/s00261-020-02796-w [published Online First: 2020/10/13]

356. Goldberg-Stein S, Fink A, Paroder V, et al. Abdominopelvic CT findings in patients with novel coronavirus disease 2019 (COVID-19). *Abdominal radiology (New York)* 2020;45(9):2613-23. doi: 10.1007/s00261-020-02669-2 [published Online First: 2020/08/08]

357. Guler E, Unal NG, Cinkooglu A, et al. Correlation of liver-to-spleen ratio, lung CT scores, clinical, and laboratory findings of COVID-19 patients with two consecutive CT scans. *Abdominal radiology (New York)* 2021;46(4):1543-51. doi: 10.1007/s00261-020-02805-y [published Online First: 2020/10/15]

358. Udugama B, Kadhiresan P, Kozlowski HN, et al. Diagnosing COVID-19: The Disease and Tools for Detection. *ACS nano* 2020;14(4):3822-35. doi: 10.1021/acsnano.0c02624 [published Online First: 2020/04/01]

359. Shammus R, Mahbub S, Rauf MA, et al. The role of imaging and other diagnostic approaches in COVID-19. *Acta bio-medica : Atenei Parmensis* 2020;91(3):e2020019. doi: 10.23750/abm.v91i3.9822 [published Online First: 2020/09/15]

360. Milas S, Poncelet A, Buttafuoco F, et al. Antibiotic use in patients with Coronavirus disease 2019 (COVID-19): outcomes and associated factors. *Acta Clin Belg* 2022;77(3):579-87. doi: 10.1080/17843286.2021.1916300 [published Online First: 2021/04/27]

361. Dixon RV, Skaria E, Lau WM, et al. Microneedle-based devices for point-of-care infectious disease diagnostics. *Acta pharmaceutica Sinica B* 2021;11(8):2344-61. doi: 10.1016/j.apsb.2021.02.010 [published Online First: 2021/06/22]

362. Dhillon PS, Pointon K, Lenthall R, et al. Regional Mechanical Thrombectomy Imaging Protocol in Patients Presenting with Acute Ischemic Stroke during the COVID-19 Pandemic. *AJNR American journal of neuroradiology* 2020;41(10):1849-55. doi: 10.3174/ajnr.A6754 [published Online First: 2020/08/21]

363. Katz JM, Libman RB, Wang JJ, et al. COVID-19 Severity and Stroke: Correlation of Imaging and Laboratory Markers. *AJNR American journal of neuroradiology* 2021;42(2):257-61. doi: 10.3174/ajnr.A6920 [published Online First: 2020/10/31]

364. Liu D, Li L, Wu X, et al. Pregnancy and Perinatal Outcomes of Women With Coronavirus Disease (COVID-19) Pneumonia: A Preliminary Analysis. *AJR Am J Roentgenol* 2020;215(1):127-32. doi: 10.2214/ajr.20.23072 [published Online First: 2020/03/19]

365. Waller JV, Kaur P, Tucker A, et al. Diagnostic Tools for Coronavirus Disease (COVID-19): Comparing CT and RT-PCR Viral Nucleic Acid Testing. *AJR Am J Roentgenol* 2020;215(4):834-38. doi: 10.2214/ajr.20.23418 [published Online First: 2020/05/16]

366. Dagher GA, Ghanem AA, Haidar S, et al. The prognostic value of biomarker levels and chest imaging in patients with COVID-19 presenting to the emergency department. *The American journal of emergency medicine* 2022;59:15-23. doi: 10.1016/j.ajem.2022.06.043 [published Online First: 2022/07/01]

367. Karaali R, Topal F. Evaluating the effect of SARS-Cov-2 infection on prognosis and mortality in patients with acute pancreatitis. *The American journal of emergency medicine* 2021;49:378-84. doi: 10.1016/j.ajem.2021.06.045 [published Online First: 2021/07/12]

368. Gaston DC, Malinis M, Osborn R, et al. Clinical implications of SARS-CoV-2 cycle threshold values in solid organ transplant recipients. *American journal of transplantation : official journal of the American Society of Transplantation and the American Society of Transplant Surgeons* 2021;21(3):1304-11. doi: 10.1111/ajt.16357 [published Online First: 2020/10/13]

369. Orsi MA, Oliva G, Toluian T, et al. Feasibility, Reproducibility, and Clinical Validity of a Quantitative Chest X-Ray Assessment for COVID-19. *Am J Trop Med Hyg* 2020;103(2):822-27. doi: 10.4269/ajtmh.20-0535 [published Online First: 2020/07/04]

370. Rasmi Y, Li X, Khan J, et al. Emerging point-of-care biosensors for rapid diagnosis of COVID-19: current progress, challenges, and future prospects. *Anal Bioanal Chem* 2021;413(16):4137-59. doi: 10.1007/s00216-021-03377-6 [published Online First: 2021/05/20]

371. Lim I, Gautheret-Dejean A. [Rapid SARS-CoV-2 antigenic test: definition, legislation of use, technological principles, analytical and clinical performance comparison]. *Annales de biologie clinique* 2021;79(2):123-42. doi: 10.1684/abc.2021.1635 [published Online First: 2021/04/22]

372. Zhou H, Xu K, Shen Y, et al. Coronavirus disease 2019 (COVID-19): chest CT characteristics benefit to early disease recognition and patient classification-a single center experience. *Annals of translational medicine* 2020;8(11):679. doi: 10.21037/atm-20-2119a [published Online First: 2020/07/04]

373. Kovoor JG, Tivey DR, Williamson P, et al. Screening and testing for COVID-19 before surgery. *ANZ J Surg* 2020;90(10):1845-56. doi: 10.1111/ans.16260 [published Online First: 2020/08/10]

374. Nachon-Acosta A, Martinez-Mier G, Flores-Gamboa V, et al. Surgical Outcomes During COVID-19 Pandemic. *Arch Med Res* 2021;52(4):434-42. doi: 10.1016/j.arcmed.2021.01.003 [published Online First: 2021/02/24]

375. Roden AC, Boland JM, Johnson TF, et al. Late Complications of COVID-19. *Archives of pathology & laboratory medicine* 2022;146(7):791-804. doi: 10.5858/arpa.2021-0519-SA [published Online First: 2022/03/24]

376. Yuan H, Cao X, Ji X, et al. An Updated Understanding of the Current Emerging Respiratory Infection: COVID-19. *Biomed Res Int* 2020;2020:6870512. doi: 10.1155/2020/6870512 [published Online First: 2020/11/03]

377. Cui F, Zhou HS. Diagnostic methods and potential portable biosensors for coronavirus disease 2019. *Biosensors & bioelectronics* 2020;165:112349. doi: 10.1016/j.bios.2020.112349 [published Online First: 2020/06/09]

378. Li Y, Xia L. Coronavirus Disease 2019 (COVID-19): Role of Chest CT in Diagnosis and Management. *AJR Am J Roentgenol* 2020:1-7. doi: 10.2214/ajr.20.22954

379. Wang P, Anderson N, Pan Y, et al. The SARS-CoV-2 Outbreak: Diagnosis, Infection Prevention, and Public Perception. *Clinical chemistry* 2020 doi: 10.1093/clinchem/hvaa080

380. Annweiler C, Beaudenon M, Gautier J, et al. COvid-19 and high-dose VITamin D supplementation TRIAL in high-risk older patients (COVIT-TRIAL): study protocol for a randomized controlled trial. *Trials* 2020;21(1):1031. doi: 10.1186/s13063-020-04928-5

381. Cuadrado-Lavin A, Olmos JM, Cifrian JM, et al. Controlled, double-blind, randomized trial to assess the efficacy and safety of hydroxychloroquine chemoprophylaxis in SARS CoV2 infection in healthcare personnel in the hospital setting: a structured summary of a study protocol for a randomised controlled trial. *Trials* 2020;21(1):472‐. doi: 10.1186/s13063-020-04400-4

382. Daval M, Corre A, Palpacuer C, et al. Efficacy of local budesonide therapy in the management of persistent hyposmia in COVID-19 patients without signs of severity: A structured summary of a study protocol for a randomised controlled trial. *Trials* 2020;21(1) doi: 10.1186/s13063-020-04585-8

383. Daval M, Corré A, Palpacuer C, et al. Efficacy of local budesonide therapy in the management of persistent hyposmia in COVID-19 patients without signs of severity: A structured summary of a study protocol for a randomised controlled trial. *Trials* 2020;21(1) doi: 10.1186/s13063-020-04585-8

384. Dhillon PS, Pointon K, Lenthall R, et al. Regional mechanical thrombectomy imaging protocol in patients presenting with acute ischemic stroke during the COVID-19 pandemic. *American Journal of Neuroradiology* 2020;41(10):1849-55. doi: 10.3174/ajnr.A6754

385. Dushianthan A, Clark H, Madsen J, et al. Nebulised surfactant for the treatment of severe COVID-19 in adults (COV-Surf): a structured summary of a study protocol for a randomized controlled trial. *Trials* 2020;21(1):1014. doi: 10.1186/s13063-020-04944-5

386. Emadi A, Chua JV, Talwani R, et al. Safety and Efficacy of Imatinib for Hospitalized Adults with COVID-19: a structured summary of a study protocol for a randomised controlled trial. *Trials* 2020;21(1) doi: 10.1186/s13063-020-04819-9

387. Gopel S, Bethge W, Martus P, et al. Test and treat COVID 65 plus-Hydroxychloroquine versus placebo in early ambulatory diagnosis and treatment of older patients with COVID19: a structured summary of a study protocol for a randomised controlled trial. *Trials* 2020;21(1) doi: 10.1186/s13063-020-04556-z

388. Abdel-Tawab M, Basha MAA, Mohamed IAI, et al. Comparison of the CO-RADS and the RSNA chest CT classification system concerning sensitivity and reliability for the diagnosis of COVID-19 pneumonia. *Insights Imaging* 2021;12(1):55. doi: 10.1186/s13244-021-00998-4 [published Online First: 2021/04/30]

389. Bayramoglu Z, Canıpek E, Comert RG, et al. Imaging Features of Pediatric COVID-19 on Chest Radiography and Chest CT: A Retrospective, Single-Center Study. *Acad Radiol* 2021;28(1):18-27. doi: 10.1016/j.acra.2020.10.002 [published Online First: 2020/10/18]

390. Bernheim AA-O, Mei XA-O, Huang M, et al. Chest CT Findings in Coronavirus Disease-19 (COVID-19): Relationship to Duration of Infection. 2020(1527-1315 (Electronic))

391. Cao M, Zhang D, Wang Y, et al. Clinical Features of Patients Infected with the 2019 Novel Coronavirus (COVID-19) in Shanghai, China. *medRxiv* 2020 doi: 10.1101/2020.03.04.20030395 [published Online First: 2020/06/09]

392. Chate RC, Fonseca E, Passos RBD, et al. Presentation of pulmonary infection on CT in COVID-19: initial experience in Brazil. *Jornal brasileiro de pneumologia : publicacao oficial da Sociedade Brasileira de Pneumologia e Tisilogia* 2020;46(2):e20200121. doi: 10.36416/1806-3756/e20200121 [published Online First: 2020/04/16]

393. Chen Z, Fan H, Cai J, et al. High-resolution computed tomography manifestations of COVID-19 infections in patients of different ages. *European journal of radiology* 2020;126:108972. doi: 10.1016/j.ejrad.2020.108972 [published Online First: 2020/04/03]

394. Chung M, Bernheim A, Mei X, et al. CT Imaging Features of 2019 Novel Coronavirus (2019-nCoV). *Radiology* 2020;295(1):202-07. doi: 10.1148/radiol.2020200230 [published Online First: 2020/02/06]

395. Fang Y, Zhang H, Xie J, et al. Sensitivity of Chest CT for COVID-19: Comparison to RT-PCR. *Radiology* 2020;296(2):E115-E17.

396. Fu H, Xu H, Zhang N, et al. Association between Clinical, Laboratory and CT Characteristics and RT-PCR Results in the Follow-up of COVID-19 patients. *medRxiv* 2020:2020.03.19.20038315. doi: 10.1101/2020.03.19.20038315

397. Guan CS, Lv ZB, Yan S, et al. Imaging Features of Coronavirus disease 2019 (COVID-19): Evaluation on Thin-Section CT. *Acad Radiol* 2020;27(5):609-13. doi: 10.1016/j.acra.2020.03.002 [published Online First: 2020/03/25]

398. Guan WJ, Ni ZY, Hu Y, et al. Clinical Characteristics of Coronavirus Disease 2019 in China. *N Engl J Med* 2020;382(18):1708-20. doi: 10.1056/NEJMoa2002032 [published Online First: 2020/02/29]

399. Hafiz M, Icksan AG, Harlivasari AD, et al. Association between clinical, laboratory findings and chest CT in COVID-19 in a secondary hospital in Jakarta, Indonesia. *Germs* 2021;11(1):32-38.

400. Han R, Huang L, Jiang H, et al. Early Clinical and CT Manifestations of Coronavirus Disease 2019 (COVID-19) Pneumonia. *AJR Am J Roentgenol* 2020;215(2):338-43. doi: 10.2214/ajr.20.22961 [published Online First: 2020/03/18]

401. Han X, Cao Y, Jiang N, et al. Novel Coronavirus Disease 2019 (COVID-19) Pneumonia Progression Course in 17 Discharged Patients: Comparison of Clinical and Thin-Section Computed Tomography Features During Recovery. *Clin Infect Dis* 2020;71(15):723-31.

402. Himoto Y, Sakata A, Kirita M, et al. Diagnostic performance of chest CT to differentiate COVID-19 pneumonia in non-high-epidemic area in Japan. *Japanese journal of radiology* 2020;38(5):400-06. doi: 10.1007/s11604-020-00958-w [published Online First: 2020/04/02]

403. Huang Y, Yang R, Xu Y, et al. Clinical characteristics of 36 non-survivors with COVID-19 in Wuhan, China: medRxiv, 2020.

404. Kant A, Kostakoglu U, Atalar S, et al. The relationship between diagnostic value of chest computed tomography imaging and symptom duration in COVID infection. *Ann Thorac Med* 2020;15(3):151-54. doi: 10.4103/atm.ATM_165_20 [published Online First: 2020/08/25]

405. Kassem MNE, Masallat DT. Clinical Application of Chest Computed Tomography (CT) in Detection and Characterization of Coronavirus (Covid-19) Pneumonia in Adults. *J Digit Imaging* 2021;34(2):273-83. doi: 10.1007/s10278-021-00426-5 [published Online First: 2021/02/11]

406. Korkmaz I, Keles F. COVID-19-Related Lung Involvement at Different Time Intervals: Evaluation of Computed Tomography Images With Semiquantitative Scoring System and COVID-19 Reporting and Data System Scoring. *Cureus* 2021;13(10):e18554. doi: 10.7759/cureus.18554 [published Online First: 2021/11/13]

407. Lei P, Huang Z, Liu G, et al. Clinical and computed tomographic (CT) images characteristics in the patients with COVID-19 infection: What should radiologists need to know? *J Xray Sci Technol* 2020;28(3):369-81. doi: 10.3233/xst-200670 [published Online First: 2020/04/14]

408. Leonard-Lorant I, Severac F, Bilbault P, et al. Normal chest CT in 1091 symptomatic patients with confirmed Covid-19: frequency, characteristics and outcome. *Eur Radiol* 2021;31(7):5172-77. doi: 10.1007/s00330-020-07593-z [published Online First: 2021/01/14]

409. Li K, Fang Y, Li W, et al. CT image visual quantitative evaluation and clinical classification of coronavirus disease (COVID-19). *Eur Radiol* 2020;30(8):4407-16. doi: 10.1007/s00330-020-06817-6 [published Online First: 2020/03/28]

410. Li X, Zeng W, Li X, et al. CT imaging changes of corona virus disease 2019(COVID-19): a multi-center study in Southwest China. *J Transl Med* 2020;18(1):154. doi: 10.1186/s12967-020-02324-w [published Online First: 2020/04/08]

411. Li Y, Xia L. Coronavirus Disease 2019 (COVID-19): Role of Chest CT in Diagnosis and Management. *AJR Am J Roentgenol* 2020;214(6):1280-86. doi: 10.2214/AJR.20.22954 [published Online First: 2020/03/05]

412. Liang Y, Liang J-j, Zhou Q, et al. Prevalence and clinical features of 2019 novel coronavirus disease (COVID-19) in the Fever Clinic of a teaching hospital in Beijing: a single-center, retrospective study. *medRxiv* 2020

413. Liao J, Fan S, Chen J, et al. Epidemiological and Clinical Characteristics of COVID-19 in Adolescents and Young Adults. *Innovation (Camb)* 2020;1(1):100001. doi: 10.1016/j.xinn.2020.04.001 [published Online First: 2021/02/09]

414. Lin Y, Lv S, Wang J, et al. Ultra-High-Resolution CT Follow-Up in Patients with Imported Early-Stage Coronavirus Disease 2019 (COVID-19) Related Pneumonia: medRxiv, 2020.

415. Ling Z, Xu X, Gan Q, et al. Asymptomatic SARS-CoV-2 infected patients with persistent negative CT findings. *European journal of radiology* 2020;126:108956. doi: 10.1016/j.ejrad.2020.108956 [published Online First: 2020/03/22]

416. 刘荣荣, 朱怡, 吴敏昱, et al. 33例新型冠状病毒感染患者的肺部ct影像学分析. *中华医学杂志* 2020(13):1007-08-09-10-11.

417. Liu W, Tao ZW, Wang L, et al. Analysis of factors associated with disease outcomes in hospitalized patients with 2019 novel coronavirus disease. *Chin Med J (Engl)* 2020;133(9):1032-38. doi: 10.1097/CM9.0000000000000775 [published Online First: 2020/03/03]

418. Lomoro P, Verde F, Zerboni F, et al. COVID-19 pneumonia manifestations at the admission on chest ultrasound, radiographs, and CT: single-center study and comprehensive radiologic literature review. *Eur J Radiol Open* 2020;7:100231. doi: 10.1016/j.ejro.2020.100231 [published Online First: 2020/04/15]

419. Long C, Xu H, Shen Q, et al. Diagnosis of the Coronavirus disease (COVID-19): rRT-PCR or CT? *European journal of radiology* 2020;126:108961. doi: 10.1016/j.ejrad.2020.108961 [published Online First: 2020/04/02]

420. Pan F, Ye T, Sun P, et al. Time Course of Lung Changes at Chest CT during Recovery from Coronavirus Disease 2019 (COVID-19). *Radiology* 2020;295(3):715-21.

421. Ravikanth R. Diagnostic accuracy of chest computed tomography in improving the false negative rate as compared to reverse transcriptase polymerase chain reaction in coronavirus disease 2019 pneumonia: A cross sectional analysis of 348 cases from India. *Lung India* 2021;38(Supplement):S11-s21. doi: 10.4103/lungindia.lungindia_410_20 [published Online First: 2021/03/10]

422. Shi H, Han X, Jiang N, et al. Radiological findings from 81 patients with COVID-19 pneumonia in Wuhan, China: a descriptive study. *The Lancet Infectious Diseases* 2020;20(4):425-34. doi: 10.1016/s1473-3099(20)30086-4

423. Shu L, Wang X, Li M, et al. Clinical characteristics of moderate COVID-19 patients aggravation in Wuhan Stadium Cabin Hospital: A 571 cases of retrospective cohort study. *J Med Virol* 2021;93(2):1133-40. doi: 10.1002/jmv.26414 [published Online First: 2020/08/12]

424. 孙潺, 张轩斌, 代岩, et al. 河南省南阳市150例新型冠状病毒感染患者临床特征分析. *中华结核和呼吸杂志* 2020(06):503-08.

425. Tan YP, Tan BY, Pan J, et al. Epidemiologic and clinical characteristics of 10 children with coronavirus disease 2019 in Changsha, China. *Journal of clinical virology : the official publication of the Pan American Society for Clinical Virology* 2020;127:104353. doi: 10.1016/j.jcv.2020.104353 [published Online First: 2020/04/18]

426. Tekcan Sanli DE, Yildirim D, Sanli AN, et al. Predictive value of CT imaging findings in COVID-19 pneumonia at the time of first-screen regarding the need for hospitalization or intensive care unit. *Diagn Interv Radiol* 2021;27(5):599-606. doi: 10.5152/dir.2020.20421 [published Online First: 2020/12/09]

427. Tian S, Chang Z, Wang Y, et al. Clinical Characteristics and Reasons for Differences in Duration From Symptom Onset to Release From Quarantine Among Patients With COVID-19 in Liaocheng, China. *Front Med (Lausanne)* 2020;7:210. doi: 10.3389/fmed.2020.00210 [published Online First: 2020/06/24]

428. Tian S, Wu M, Chang Z, et al. Epidemiological investigation and intergenerational clinical characteristics of 24 coronavirus disease patients associated with a supermarket cluster: a retrospective study. *BMC Public Health* 2021;21(1):647.

429. Wan S, Xiang Y, Fang W, et al. Clinical features and treatment of COVID-19 patients in northeast Chongqing. *J Med Virol* 2020;92(7):797-806. doi: 10.1002/jmv.25783 [published Online First: 2020/03/22]

430. 王端, 鞠秀丽, 谢峰, et al. 中国北方六省(自治区)儿童2019新型冠状病毒感染31例临床分析. *中华儿科杂志* 2020(04):269-70-71-72-73-74.

431. 王锦程, 刘锦鹏, 王园园, et al. 2019冠状病毒病(Covid-19)患者胸部ct影像学动态变化. *浙江大学学报(医学版)* 2020(02 vo 49):191-97.

432. Wang L, Duan Y, Zhang W, et al. Epidemiologic and Clinical Characteristics of 26 Cases of COVID-19 Arising from Patient-to-Patient Transmission in Liaocheng, China. *Clin Epidemiol* 2020;12:387-91. doi: 10.2147/CLEP.S249903 [published Online First: 2020/04/21]

433. Epidemical and Clinical Characteristics of 165 Patients Infected with SARS-CoV-2 in Fujian Province, China; 2020.

434. Wu J, Wu X, Zeng W, et al. Chest CT Findings in Patients With Coronavirus Disease 2019 and Its Relationship With Clinical Features. *Investigative radiology* 2020;55(5):257-61. doi: 10.1097/RLI.0000000000000670 [published Online First: 2020/02/25]

435. Xia W, Shao J, Guo Y, et al. Clinical and CT features in pediatric patients with COVID-19 infection: Different points from adults. *Pediatr Pulmonol* 2020;55(5):1169-74. doi: 10.1002/ppul.24718 [published Online First: 2020/03/07]

436. Xiong Y, Sun D, Liu Y, et al. Clinical and High-Resolution CT Features of the COVID-19 Infection: Comparison of the Initial and Follow-up Changes. *Investigative radiology* 2020;55(6):332-39. doi: 10.1097/RLI.0000000000000674 [published Online First: 2020/03/07]

437. Xu YH, Dong JH, An WM, et al. Clinical and computed tomographic imaging features of novel coronavirus pneumonia caused by SARS-CoV-2. *The Journal of infection* 2020;80(4):394-400. doi: 10.1016/j.jinf.2020.02.017 [published Online First: 2020/02/29]

438. Yang W, Cao Q, Qin L, et al. Clinical characteristics and imaging manifestations of the 2019 novel coronavirus disease (COVID-19):A multi-center study in Wenzhou city, Zhejiang, China. *The Journal of infection* 2020;80(4):388-93. doi: 10.1016/j.jinf.2020.02.016 [published Online First: 2020/03/01]

439. Zeng J, Peng S, Lei Y, et al. Clinical and Imaging features of COVID-19 Patients: Analysis of Data from High-Altitude Areas. *The Journal of infection* 2020;80(6):e34-e36. doi: 10.1016/j.jinf.2020.03.026 [published Online First: 2020/04/11]

440. Zhang X, Cai H, Hu J, et al. Epidemiological, clinical characteristics of cases of SARS-CoV-2 infection with abnormal imaging findings. *International journal of infectious diseases : IJID : official publication of the International Society for Infectious Diseases* 2020;94:81-87. doi: 10.1016/j.ijid.2020.03.040 [published Online First: 2020/03/25]

441. Adibi A, Kazemi K, Hajiahmadi S, et al. The value of thoracic computed tomography scan comparing to reverse transcription-polymerase chain reaction for the diagnosis of COVID-19. *J Res Med Sci* 2021;26:117. doi: 10.4103/jrms.JRMS_1187_20 [published Online First: 2022/02/08]

442. Ai J, Gong J, Xing L, et al. Analysis of factors associated early diagnosis in coronavirus disease 2019 (COVID-19). *medRxiv* 2020:2020.04.09.20059352. doi: 10.1101/2020.04.09.20059352

443. Ai T, Yang Z, Hou H, et al. Correlation of Chest CT and RT-PCR Testing for Coronavirus Disease 2019 (COVID-19) in China: A Report of 1014 Cases. *Radiology* 2020;296(2):E32-E40. doi: 10.1148/radiol.2020200642 [published Online First: 2020/02/27]

444. Aslan S, Bekci T, Cakir IM, et al. Diagnostic performance of low-dose chest CT to detect COVID-19: A Turkish population study. *Diagn Interv Radiol* 2021;27(2):181-87. doi: 10.5152/dir.2020.20350 [published Online First: 2020/09/03]

445. Bai HX, Hsieh B, Xiong Z, et al. Performance of Radiologists in Differentiating COVID-19 from Non-COVID-19 Viral Pneumonia at Chest CT. *Radiology* 2020;296(2):E46-E54.

446. Barbosa PNVP, Bitencourt AGV, Miranda GDd, et al. Chest CT accuracy in the diagnosis of SARS-CoV-2 infection: initial experience in a cancer center. *Radiologia Brasileira* 2020;53(4):211-15. doi: 10.1590/0100-3984.2020.0040

447. Baysal B, Dogan MB, Gulbay M, et al. Predictive performance of CT for adverse outcomes among COVID-19 suspected patients: a two-center retrospective study. *Bosnian journal of basic medical sciences* 2021;21(6):739-45. doi: 10.17305/bjbms.2020.5466 [published Online First: 2021/02/13]

448. Besutti G, Giorgi Rossi P, Iotti V, et al. Accuracy of CT in a cohort of symptomatic patients with suspected COVID-19 pneumonia during the outbreak peak in Italy. *Eur Radiol* 2020;30(12):6818-27. doi: 10.1007/s00330-020-07050-x [published Online First: 2020/07/16]

449. Bollineni VR, Nieboer KH, Doring S, et al. The role of CT imaging for management of COVID-19 in epidemic area: early experience from a University Hospital. *Insights Imaging* 2021;12(1):10. doi: 10.1186/s13244-020-00957-5 [published Online First: 2021/01/30]

450. Borakati A, Perera A, Johnson J, et al. Diagnostic accuracy of X-ray versus CT in COVID-19: a propensity-matched database study. *BMJ Open* 2020;10(11):e042946. doi: 10.1136/bmjopen-2020-042946 [published Online First: 2020/11/08]

451. Borges da Silva Teles G, Kaiser Ururahy Nunes Fonseca E, Yokoo P, et al. Performance of Chest Computed Tomography in Differentiating Coronavirus Disease 2019 From Other Viral Infections Using a Standardized Classification. *Journal of thoracic imaging* 2021;36(1):31-36. doi: 10.1097/rti.0000000000000563 [published Online First: 2020/10/02]

452. Boussouar S, Wagner M, Donciu V, et al. Diagnostic performance of chest computed tomography during the epidemic wave of COVID-19 varied as a function of time since the beginning of the confinement in France. *PLoS One* 2020;15(11):e0242840. doi: 10.1371/journal.pone.0242840 [published Online First: 2020/11/24]

453. Brun AL, Gence-Breney A, Trichereau J, et al. COVID-19 pneumonia: high diagnostic accuracy of chest CT in patients with intermediate clinical probability. *Eur Radiol* 2021;31(4):1969-77. doi: 10.1007/s00330-020-07346-y [published Online First: 2020/10/05]

454. Caruso D, Zerunian M, Polici M, et al. Chest CT Features of COVID-19 in Rome, Italy. *Radiology* 2020;296(2):E79-E85.

455. Ciccarese F, Coppola F, Spinelli D, et al. Diagnostic Accuracy of North America Expert Consensus Statement on Reporting CT Findings in Patients Suspected of Having COVID-19 Infection: An Italian Single-Center Experience. *Radiol Cardiothorac Imaging* 2020;2(4):e200312. doi: 10.1148/ryct.2020200312 [published Online First: 2021/03/30]

456. Dangis A, Gieraerts C, De Bruecker Y, et al. Accuracy and Reproducibility of Low-Dose Submillisievert Chest CT for the Diagnosis of COVID-19. *Radiol Cardiothorac Imaging* 2020;2(2):e200196. doi: 10.1148/ryct.2020200196 [published Online First: 2021/03/30]

457. De Smet K, De Smet D, Ryckaert T, et al. Diagnostic Performance of Chest CT for SARS-CoV-2 Infection in Individuals with or without COVID-19 Symptoms. *Radiology* 2021;298(1):E30-e37. doi: 10.1148/radiol.2020202708 [published Online First: 2020/08/11]

458. Dong JJ, Wu L, Jin Q, et al. Chest CT Scan of Hospitalized Patients with COVID-19: A Case-Control Study. *medRxiv* 2020

459. Ducray V, Vlachomitrou AS, Bouscambert-Duchamp M, et al. Chest CT for rapid triage of patients in multiple emergency departments during COVID-19 epidemic: experience report from a large French university hospital. *Eur Radiol* 2021;31(2):795-803. doi: 10.1007/s00330-020-07154-4 [published Online First: 2020/08/20]

460. Falaschi Z, Danna PSC, Arioli R, et al. Chest CT accuracy in diagnosing COVID-19 during the peak of the Italian epidemic: A retrospective correlation with RT-PCR testing and analysis of discordant cases. *Eur J Radiol* 2020;130:109192. doi: 10.1016/j.ejrad.2020.109192 [published Online First: 2020/08/02]

461. Farahani RH, Mosallaei M, Hazrati E, et al. Diagnostic Performance of Chest CT-Scan and First RT-PCR Testing for COVID-19 in Iranian Population. *Iran J Public Health* 2021;50(8):1740-42. doi: 10.18502/ijph.v50i8.6842 [published Online First: 2021/12/18]

462. Fujioka T, Takahashi M, Mori M, et al. Evaluation of the Usefulness of CO-RADS for Chest CT in Patients Suspected of Having COVID-19. *Diagnostics (Basel, Switzerland)* 2020;10(9) doi: 10.3390/diagnostics10090608 [published Online First: 2020/08/23]

463. Gaia C, Maria Chiara C, Silvia L, et al. Chest CT for early detection and management of coronavirus disease (COVID-19): a report of 314 patients admitted to Emergency Department with suspected pneumonia. *Radiol Med* 2020;125(10):931-42. doi: 10.1007/s11547-020-01256-1 [published Online First: 2020/07/31]

464. Gietema HA, Zelis N, Nobel JM, et al. CT in relation to RT-PCR in diagnosing COVID-19 in The Netherlands: A prospective study. *PLoS One* 2020;15(7):e0235844. doi: 10.1371/journal.pone.0235844 [published Online First: 2020/07/10]

465. Gross A, Heine G, Schwarz M, et al. Structured reporting of chest CT provides high sensitivity and specificity for early diagnosis of COVID-19 in a clinical routine setting. *The British journal of radiology* 2021;94(1117):20200574. doi: 10.1259/bjr.20200574 [published Online First: 2020/11/28]

466. Guillo E, Bedmar Gomez I, Dangeard S, et al. COVID-19 pneumonia: Diagnostic and prognostic role of CT based on a retrospective analysis of 214 consecutive patients from Paris, France. *European journal of radiology* 2020;131:109209. doi: 10.1016/j.ejrad.2020.109209 [published Online First: 2020/08/19]

467. He JL, Luo L, Luo ZD, et al. Diagnostic performance between CT and initial real-time RT-PCR for clinically suspected 2019 coronavirus disease (COVID-19) patients outside Wuhan, China. *Respir Med* 2020;168:105980. doi: 10.1016/j.rmed.2020.105980 [published Online First: 2020/05/05]

468. Hermans JJR, Groen J, Zwets E, et al. Chest CT for triage during COVID-19 on the emergency department: myth or truth? *Emerg Radiol* 2020;27(6):641-51. doi: 10.1007/s10140-020-01821-1 [published Online First: 2020/07/22]

469. Herpe G, Lederlin M, Naudin M, et al. Efficacy of Chest CT for COVID-19 Pneumonia Diagnosis in France. *Radiology* 2021;298(2):E81-e87. doi: 10.1148/radiol.2020202568 [published Online First: 2020/09/02]

470. Inui S, Kurokawa R, Nakai Y, et al. Comparison of Chest CT Grading Systems in Coronavirus Disease 2019 (COVID-19) Pneumonia. *Radiol Cardiothorac Imaging* 2020;2(6):e200492. doi: 10.1148/ryct.2020200492 [published Online First: 2021/03/30]

471. Zhifeng J, Feng A, Li T. Consistency analysis of COVID-19 nucleic acid tests and the changes of lung CT. *J Clin Virol* 2020;127:104359. doi: 10.1016/j.jcv.2020.104359 [published Online First: 2020/04/18]

472. Kızıloglu I, Sener A, Siliv N. Comparison of rapid antibody test and thorax computed tomography results in patients who underwent RT-PCR with the pre-diagnosis of COVID-19. *Int J Clin Pract* 2021;75(10):e14524. doi: 10.1111/ijcp.14524 [published Online First: 2021/06/14]

473. Kurokawa R, Inui S, Gonoi W, et al. Standardized reporting systems of chest computed tomography in a population with low coronavirus disease 2019 prevalence: A retrospective comparative study. *Heliyon* 2021;7(8):e07743. doi: 10.1016/j.heliyon.2021.e07743 [published Online First: 2021/08/17]

474. Kuzan TY, Murzoglu Altintoprak K, Ciftci HO, et al. A comparison of clinical, laboratory and chest CT findings of laboratory-confirmed and clinically diagnosed COVID-19 patients at first admission. *Diagn Interv Radiol* 2021;27(3):336-43. doi: 10.5152/dir.2020.20270 [published Online First: 2020/09/03]

475. Lieveld AWE, Kok B, Schuit FH, et al. Diagnosing COVID-19 pneumonia in a pandemic setting: Lung Ultrasound versus CT (LUVCT) - a multicentre, prospective, observational study. *ERJ Open Res* 2020;6(4) doi: 10.1183/23120541.00539-2020 [published Online First: 2021/01/15]

476. Lieveld AWE, Azijli K, Teunissen BP, et al. Chest CT in COVID-19 at the ED: Validation of the COVID-19 Reporting and Data System (CO-RADS) and CT Severity Score: A Prospective, Multicenter, Observational Study. *Chest* 2021;159(3):1126-35. doi: 10.1016/j.chest.2020.11.026 [published Online First: 2020/12/04]

477. Majeed T, Ali RS, Solomon J, et al. The Role of the Computed Tomography (CT) Thorax in the Diagnosis of COVID-19 for Patients Presenting with Acute Surgical Emergencies. A Single Institute Experience. *Indian J Surg* 2020;82(6):1005-10. doi: 10.1007/s12262-020-02626-9 [published Online First: 2020/10/27]

478. Miao C, Jin M, Miao L, et al. Early chest computed tomography to diagnose COVID-19 from suspected patients: A multicenter retrospective study. *The American journal of emergency medicine* 2021;44:346-51. doi: 10.1016/j.ajem.2020.04.051 [published Online First: 2020/04/25]

479. Miranda Magalhães Santos JM, Paula Alves Fonseca A, Pinheiro Zarattini Anastacio E, et al. Initial Results of the Use of a Standardized Diagnostic Criteria for Chest Computed Tomography Findings in Coronavirus Disease 2019. *J Comput Assist Tomogr* 2020;44(5):647-51. doi: 10.1097/rct.0000000000001054 [published Online First: 2020/07/11]

480. Ozkarafakili MA, Ozkurt H, Bardakci MI, et al. Comparison of chest computed tomography findings of RT-PCR negative and RT-PCR positive cases in COVID-19 patients. *Clin Imaging* 2022;82:7-12. doi: 10.1016/j.clinimag.2021.10.013 [published Online First: 2021/11/13]

481. SB ON, Byrne D, Muller NL, et al. Radiological Society of North America (RSNA) Expert Consensus Statement Related to Chest CT Findings in COVID-19 Versus CO-RADS: Comparison of Reporting System Performance Among Chest Radiologists and End-User Preference. *Can Assoc Radiol J* 2021;72(4):806-13. doi: 10.1177/0846537120968919 [published Online First: 2020/11/04]

482. Ohana M, Muller J, Severac F, et al. Temporal variations in the diagnostic performance of chest CT for Covid-19 depending on disease prevalence: Experience from North-Eastern France. *European journal of radiology* 2021;134:109425. doi: 10.1016/j.ejrad.2020.109425 [published Online First: 2020/12/01]

483. Ooi MWX, Liong SY, Baguley N, et al. Role of complementary Ct chest in patients presenting with acute abdominal symptoms during covid-19 pandemic: a UK experience. *Clinical imaging* 2021;69:289-92. doi: 10.1016/j.clinimag.2020.09.009 [published Online First: 2020/10/11]

484. Ozer H, Kilincer A, Uysal E, et al. Diagnostic performance of Radiological Society of North America structured reporting language for chest computed tomography findings in patients with COVID-19. *Japanese journal of radiology* 2021;39(9):877-88. doi: 10.1007/s11604-021-01128-2

485. Palmisano A, Scotti GM, Ippolito D, et al. Chest CT in the emergency department for suspected COVID-19 pneumonia. *Radiol Med* 2021;126(3):498-502. doi: 10.1007/s11547-020-01302-y [published Online First: 2020/11/10]

486. Peng D, Zhang J, Xu Y-c, et al. The Role of Procalcitonin in Early Differential Diagnosis of Suspected Children with COVID-19. *Biomedical Journal of Scientific & Technical Research* 2020

487. Ravikanth R. Diagnostic accuracy and false-positive rate of chest CT as compared to RT-PCR in coronavirus disease 2019 (COVID-19) pneumonia: A prospective cohort of 612 cases from India and review of literature. *The Indian journal of radiology & imaging* 2021;31(Suppl 1):S161-s69. doi: 10.4103/ijri.IJRI_377_20 [published Online First: 2021/04/06]

488. Reginelli A, Grassi R, Feragalli B, et al. Coronavirus Disease 2019 (COVID-19) in Italy: Double Reading of Chest CT Examination. *Biology (Basel)* 2021;10(2) doi: 10.3390/biology10020089 [published Online First: 2021/01/29]

489. Revel MP, Boussouar S, de Margerie-Mellon C, et al. Study of Thoracic CT in COVID-19: The STOIC Project. *Radiology* 2021;301(1):E361-e70. doi: 10.1148/radiol.2021210384 [published Online First: 2021/06/30]

490. Rona G, Voyvoda N, Arifoglu M, et al. The Efficacy of Chest Computed Tomography in Pediatric Patients With Suspected COVID-19. *J Comput Assist Tomogr* 2021;45(2):337-41. doi: 10.1097/RCT.0000000000001127 [published Online First: 2020/11/14]

491. Salehi-Pourmehr H, Pourfathi H, Tarzamni MK, et al. Diagnostic value of chest CT in Iranian patients with suspected COVID-19. *Caspian J Intern Med* 2020;11(Suppl 1):527-30. doi: 10.22088/cjim.11.0.527 [published Online First: 2021/01/12]

492. Schalekamp S, Bleeker-Rovers CP, Beenen LFM, et al. Chest CT in the Emergency Department for Diagnosis of COVID-19 Pneumonia: Dutch Experience. *Radiology* 2021;298(2):E98-e106. doi: 10.1148/radiol.2020203465 [published Online First: 2020/11/18]

493. Skalidis I, Nguyen VK, Bothorel H, et al. Unenhanced computed tomography (CT) utility for triage at the emergency department during COVID-19 pandemic. *The American journal of emergency medicine* 2021;46:260-65. doi: 10.1016/j.ajem.2020.07.058 [published Online First: 2020/10/14]

494. Song S, Wu F, Liu Y, et al. Correlation Between Chest CT Findings and Clinical Features of 211 COVID-19 Suspected Patients in Wuhan, China. *Open Forum Infect Dis* 2020;7(6):ofaa171. doi: 10.1093/ofid/ofaa171 [published Online First: 2020/06/11]

495. Teichgräber U, Malouhi A, Ingwersen M, et al. Ruling out COVID-19 by chest CT at emergency admission when prevalence is low: the prospective, observational SCOUT study. *Respir Res* 2021;22(1):13. doi: 10.1186/s12931-020-01611-w [published Online First: 2021/01/14]

496. Thomas C, Naudin M, Tasu JP, et al. Efficacy of chest CT scan for COVID-19 diagnosis in a low prevalence and incidence region. *Eur Radiol* 2021;31(11):8141-46. doi: 10.1007/s00330-021-07863-4 [published Online First: 2021/04/20]

497. Van Berkel B, Vandevenne J, Coursier K, et al. Chest CT Diagnosis of COVID-19: Accuracy using CO-RADS and CT-Involvement Scoring. *J Belg Soc Radiol* 2021;105(1):17. doi: 10.5334/jbsr.2342 [published Online First: 2021/04/20]

498. Mehta V, Jyoti D, Guria RT, et al. Correlation between chest CT and RT-PCR testing in India's second COVID-19 wave: a retrospective cohort study. *BMJ Evid Based Med* 2022 doi: 10.1136/bmjebm-2021-111801 [published Online First: 2022/01/22]

499. Wang Y, Hou H, Wang W, et al. Combination of CT and RT-PCR in the screening or diagnosis of COVID-19. *J Glob Health* 2020;10(1):010347. doi: 10.7189/jogh.10.010347 [published Online First: 2020/05/07]

500. Wen Z, Chi Y, Zhang L, et al. Coronavirus Disease 2019: Initial Detection on Chest CT in a Retrospective Multicenter Study of 103 Chinese Patients. *Radiol Cardiothorac Imaging* 2020;2(2):e200092. doi: 10.1148/ryct.2020200092 [published Online First: 2021/03/30]

501. 熊曾, 傅蕾, 周晖, et al. 新型冠状病毒肺炎诊断流程的构建及评价. *中华医学杂志* 2020(16):1223-24-25-26-27-28-29.

502. Yang H, Sun G, Tang F, et al. Clinical features and outcomes of pregnant women suspected of coronavirus disease 2019. *The Journal of infection* 2020;81(1):e40-e44. doi: 10.1016/j.jinf.2020.04.003 [published Online First: 2020/04/16]

**Appendix 3.** Forest plot of likelihood ratios for positive test results of CT in confirmed cases for predicting COVID-19 diagnosis


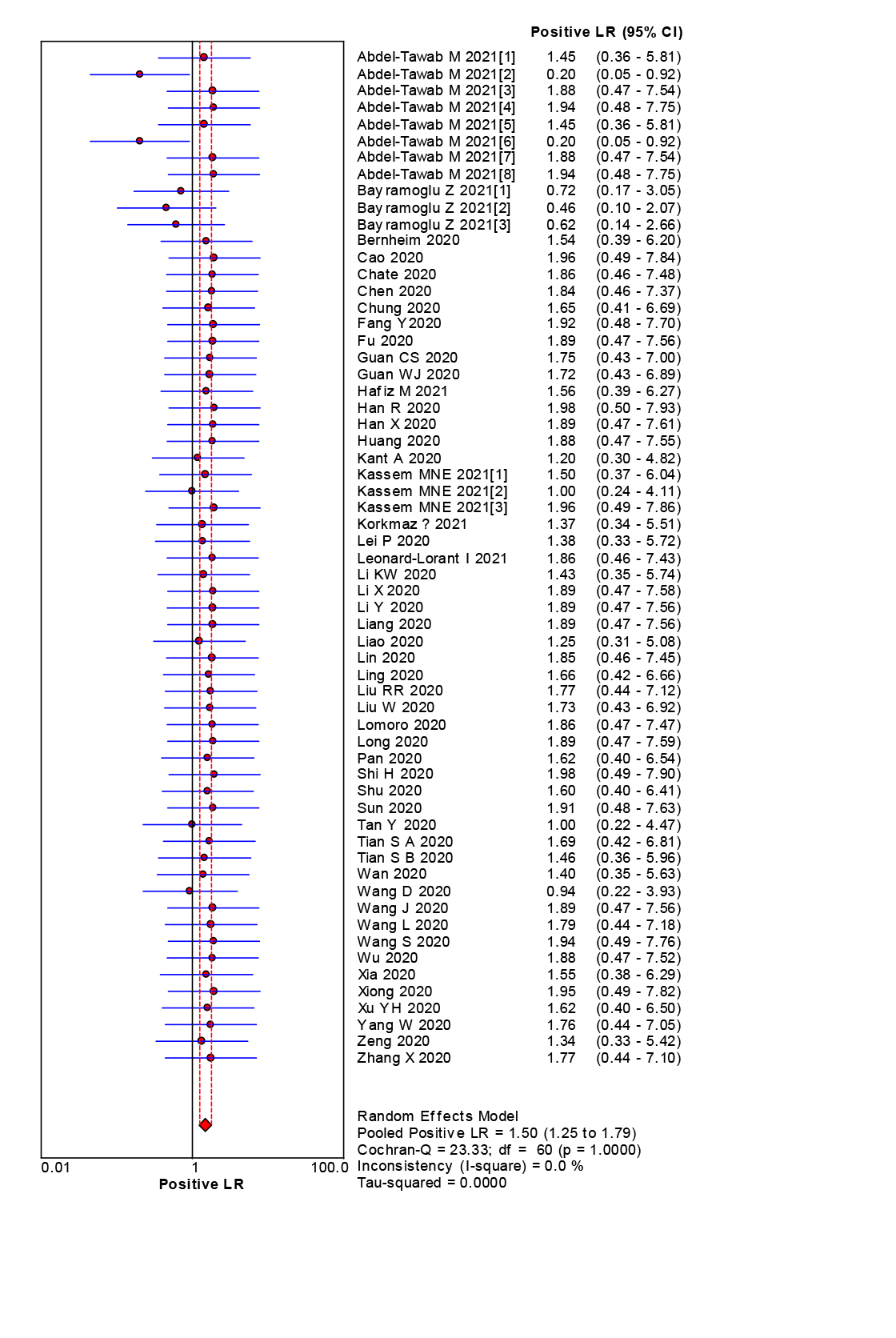


**Appendix 4.** Forest plot of likelihood ratios for negative test results of CT in confirmed cases for predicting COVID-19 diagnosis


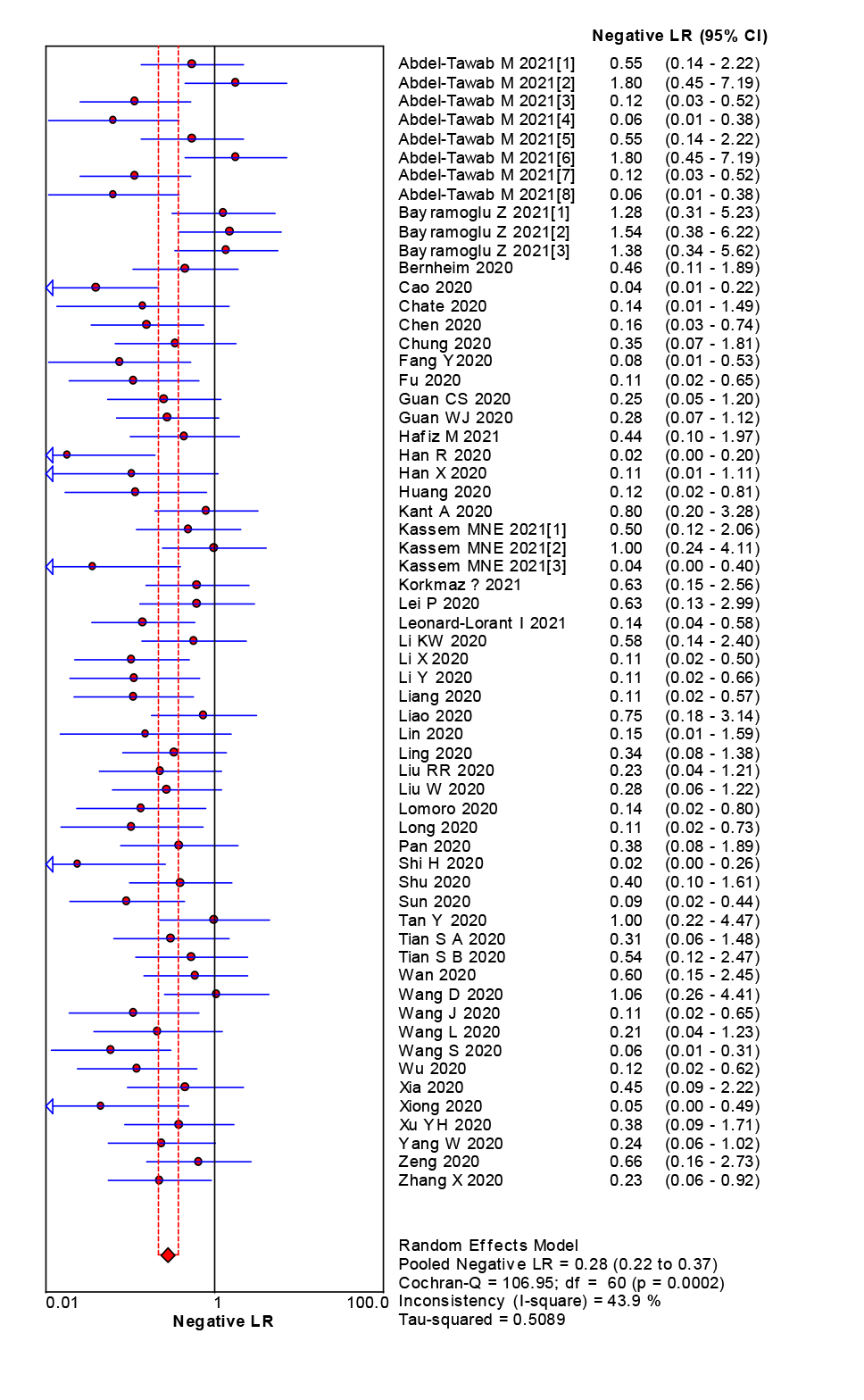


**Appendix 5.** Forest plot of likelihood ratios for positive test results of CT in suspected cases for predicting COVID-19 diagnosis


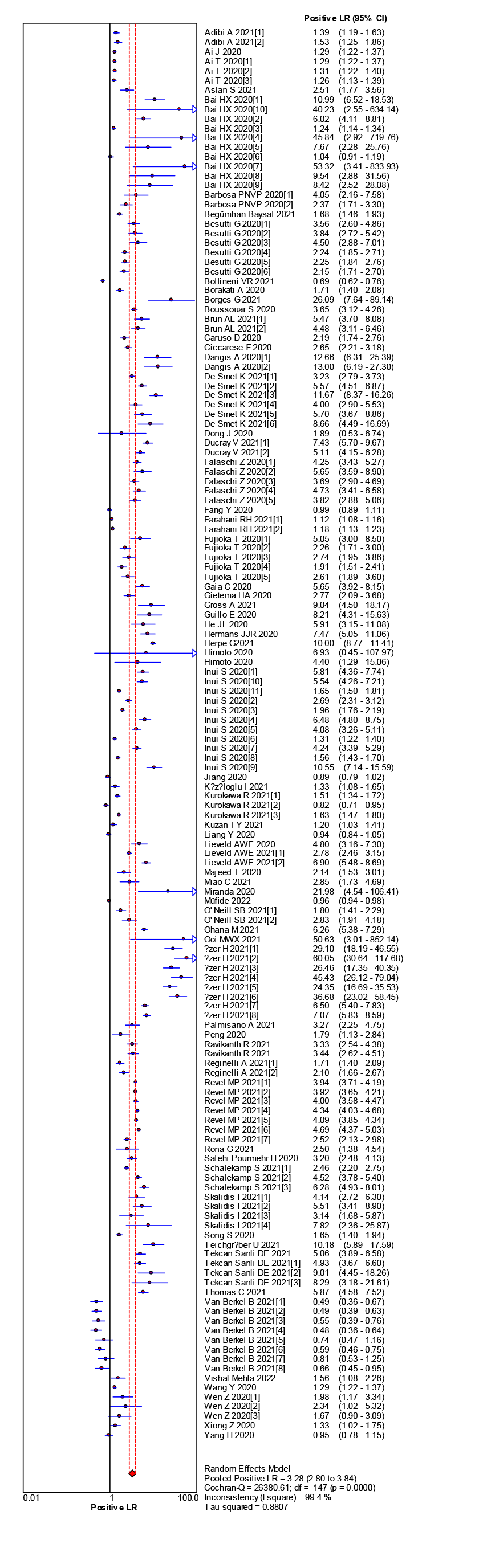


**Appendix 6.** Forest plot of likelihood ratios for negative test results of CT in suspected cases for predicting COVID-19 diagnosis


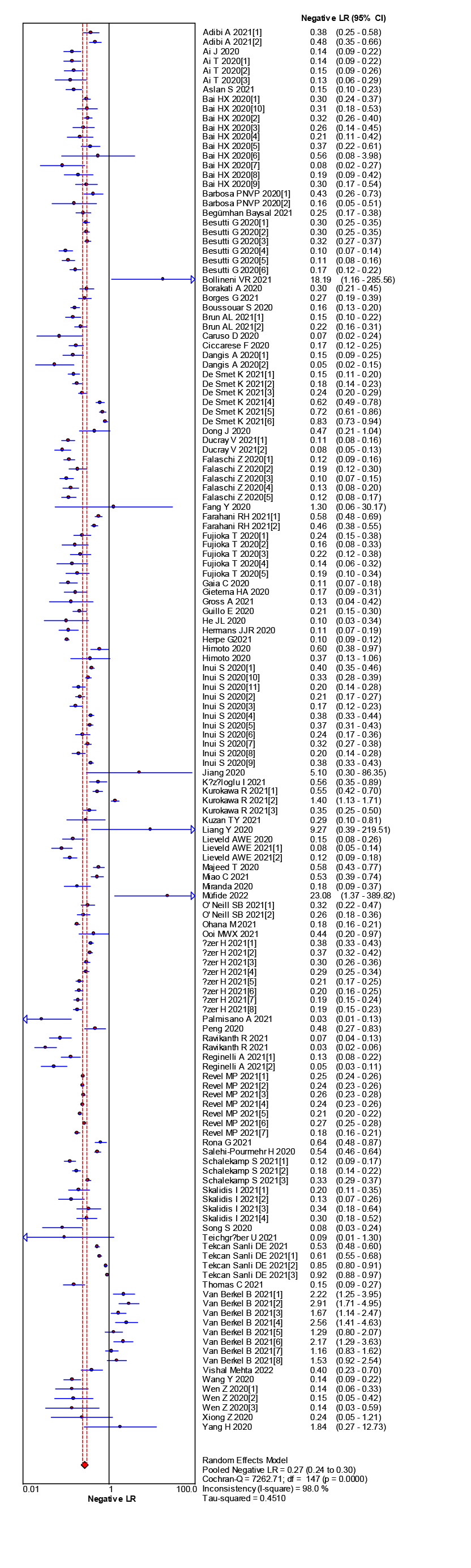

Supplement: Supplementary file 1 [file Table_1.DOC]
